# Heterochromatome wide analyses reveal MBD2 as a phase separation scaffold for heterochromatin compartmentalization and composition

**DOI:** 10.1093/nar/gkaf1380

**Published:** 2025-12-19

**Authors:** Hui Zhang, Enes Ugur, Christian Hake, Hector Romero, Maria Arroyo, Marah Mahmoud, Frederik Lermyte, Heinrich Leonhardt, M Cristina Cardoso

**Affiliations:** Department of Biology, Technical University of Darmstadt, 64287 Darmstadt, Germany; Faculty of Biology and Center for Molecular Biosystems (BioSysM), Human Biology and BioImaging, Ludwig-Maximilians-Universität München, 81377 Munich, Germany; Department of Proteomics and Signal Transduction, Max-Planck Institute of Biochemistry, 82152 Martinsried, Germany; Clemens-Schöpf Institute of Organic Chemistry and Biochemistry, Department of Chemistry, Technical University of Darmstadt, 64287 Darmstadt, Germany; Department of Biology, Technical University of Darmstadt, 64287 Darmstadt, Germany; Department of Biology, Technical University of Darmstadt, 64287 Darmstadt, Germany; Department of Biology, Technical University of Darmstadt, 64287 Darmstadt, Germany; Clemens-Schöpf Institute of Organic Chemistry and Biochemistry, Department of Chemistry, Technical University of Darmstadt, 64287 Darmstadt, Germany; Faculty of Biology and Center for Molecular Biosystems (BioSysM), Human Biology and BioImaging, Ludwig-Maximilians-Universität München, 81377 Munich, Germany; Department of Biology, Technical University of Darmstadt, 64287 Darmstadt, Germany

## Abstract

Heterochromatin is essential for nuclear integrity, genome stability, and gene regulation. However, the mechanisms governing heterochromatin compartmentalization remain poorly understood. Recent studies suggest that phase separation underlies the organization of heterochromatin. Here, we integrated quantitative spatial proteomics, phase separation assays, and phase separation prediction tools to identify and characterize candidate phase separation scaffold proteins involved in heterochromatin compartmentalization. We *in vitro* reconstituted phase-separated heterochromatin condensates using heterochromatin fractions isolated from mouse brain. Mass spectrometric analysis yielded around 1000 proteins from which 250 were predicted to have scaffold phase separation properties using machine learning-based phase separation protein prediction tools. From these, 20 proteins, including methyl-CpG binding domain protein 2 (MBD2), were localized to pericentric heterochromatin compartments using gene ontology annotation analysis. We demonstrated that MBD2 undergoes liquid–liquid phase separation via C-terminus-mediated homo-oligomerization, forming liquid-like condensates that regulate heterochromatin compartmentalization. Moreover, we found that MBD2a and MBD2b exclude histone acetyltransferases (e.g. Kat7) and recruit histone deacetylases (e.g. HDAC11, GATAD2b) at pericentric heterochromatin, resulting in subsequent deacetylation of histone H3 K27 and K9 within heterochromatin. This study advances our understanding of heterochromatin compartmentalization and highlights the role of MBD2 in heterochromatin dynamics and composition functionally regulating chromatin states.

## Graphical Abstract

**Graphical Abstract:**
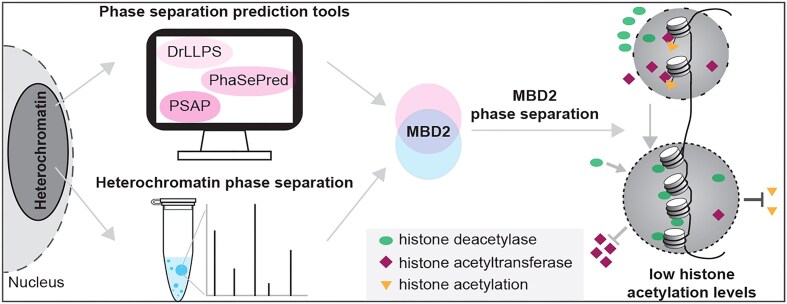

## Introduction

Heterochromatin is a fundamental component of eukaryotic genomes, characterized by its dense compaction and high DNA methylation at cytosines (mC) [1, 2]. Heterochromatin is primarily located at the nuclear periphery and around centromeric and telomeric regions and plays essential roles in ensuring genome stability and regulating gene expression [3]. The constitutive pericentric heterochromatin is particularly important for proper chromatin segregation during mitosis as well as spatial chromosome organization in interphase [3] yet the precise mechanisms governing its organization and compartmentalization remain incompletely understood. Recent advances in spatial proteomics (nuclei fractionation coupled mass spectrometry) enabled the identification of proteins enriched in specific nuclear fractions, including global chromatin, heterochromatin and pericentric heterochromatin [4–6]. Yet, how these proteins collaborate within specific subnuclear structures to regulate their dynamics remains to be explored. Additionally, nonhistone scaffold proteins, which bind to DNA and recruit specific proteins involved in similar/relevant cellular activities, are pivotal in understanding heterochromatin organization. Yet, a proper way to identify the scaffold proteins involved in heterochromatin organization and genome silencing is still lacking. We hypothesized that integrating advanced nuclei fractionation-coupled mass spectrometry with prediction tools for scaffold proteins could significantly enhance the identification of nonhistone scaffold proteins critical for heterochromatin compartmentalization and function.

Phase separation has recently been recognized as a possible alternative mechanism driving the organization of various membraneless subnuclear compartments, including pericentric heterochromatin. Phase separation is a protein demixing process forming liquid-like condensates with distinct biochemical properties, allowing them to enrich specific molecules via protein-protein interactions while excluding others [7]. While several well-known proteins like heterochromatin protein 1 (HP1) [8–10] and methyl-CpG binding protein 2 (MeCP2) [11–13] have been identified to regulate pericentric heterochromatin compartmentalization via phase separation, the full spectrum of pericentric heterochromatin phase separation-related proteins remains largely unexplored. Also unknown is whether and to what extent single proteins influence the composition and organization of heterochromatin via phase separation. Current research focusing on uncovering the molecular principles underlying phase separation has clarified that phase separation is generally mediated by multivalent weak interactions between intrinsically disordered region (IDR) regions with distinct amino acid (aa) features such as prion-like domains, RGG motifs, and RG and RY repeats, which together are proposed to support phase separation via electrostatic, hydrophobic, and (cation) Pi–Pi interactions [4, 14–15]. These indicate that the phase separation of proteins is potentially encoded in its aa sequence, which has fuelled the development of several machine learning-based prediction tools designed to identify candidate phase separation scaffold proteins [16–18].

In this study, we employed an integrated approach combining spatial proteomics as well as native mass spectrometry, *in vitro* phase separation assays, and advanced phase separation prediction tools (PS predictors) [16–18] to systematically identify and characterize candidate scaffold proteins involved in heterochromatin compartmentalization. Given the link between aberrant protein phase separation and neurological diseases, such as MeCP2-related Rett syndrome [13], the mouse brain was taken as the experiment model. By isolating heterochromatin fractions from mouse brain, followed by *in vitro* phase separation and quantitative mass spectrometry, we identified a broad range of proteins associated with heterochromatin phase separation. However, not all of them directly contribute to the heterochromatin organization via phase separation. Recent research has classified phase separation proteins into four categories: scaffolds (proteins that drive phase separation), regulators (proteins that are required for the formation of liquid condensates through, for example, placing post-translational modifications (PTMs) on scaffold proteins), clients (proteins that are recruited to liquid condensates via the scaffolds), and others (proteins that are not present in either of these categories) [17]. To refine our findings, potential phase separation scaffold proteins across the mouse proteome were identified by a combination of several phase separation predictors and integrated with our heterochromatin phase separation proteomic results to pinpoint key scaffold proteins in pericentric heterochromatin. Among the candidates, methyl-CpG binding domain protein 2 (MBD2) emerged as a potential key phase separation scaffold protein in pericentric heterochromatin compartmentalization. MBD2, a member of the NuRD (nucleosome remodeling and deacetylase) complex, is involved in various cellular processes, including transcriptional repression, DNA methylation, and chromatin remodelling (reviewed in [19]. Through a combination of *in vitro* and *in vivo* experiments, coupled with protein structure predictions (AlphaFold Server), we explored the phase separation properties of MBD2 and its role in organizing pericentric heterochromatin. Furthermore, using a combination of quantitative mass spectrometry and phase separation, we established and validated the role of MBD2 in recruiting as well as excluding other heterochromatin components. Our findings provide insights into the mechanisms of heterochromatin compartmentalization and underscore the potential of MBD2 phase separation as a regulator of pericentric heterochromatin dynamics.

## Materials and methods

### Plasmids

For protein purification from bacteria, the pTYB1-MeCP2wt (pc1294) plasmid [20] was modified to generate various constructs. MBD2 and MBD2∆N coding sequences were amplified by polymerase chain reaction (PCR) from peMBD2G (pc2399) using primer pairs containing NdeI restriction enzyme (NEB, R0111S) for the forward primer and EcoRI restriction enzyme (NEB, R0101S) for the reverse primer (Supplementary Table S2). MBD3 coding sequence was amplified by PCR from pGFP-MBD3 (pc1193) using primer pairs containing NdeI (NEB, R0111S) and EcoRI (NEB, R0101S) cutting sizes for the forward primer and the reverse primer, respectively (Supplementary Table S2). Then, the amplified coding sequences were digested with NdeI/EcoRI and ligated into the NdeI/EcoRI digested pTYB1-MeCP2 vector to generate the pTYB1-MBD2 and MBD3 constructs: MBD2, MBD2∆N, and MBD3. The Q5 site-directed mutagenesis strategy was then adopted following the manufacturer’s protocol (NEB, cat. no.: E0554S) using the primer pairs listed in Supplementary Table S2 to generate the truncated versions of MBD2: MBD2-C, MBD2∆C, MBD2-N, MBD2∆CC, MBD2∆GR, MBD2∆N∆CC, MBD2-C∆CC, MBD2-MBDTRD, MBD2∆C, MBD2∆C∆GR, MBD2-N, MBD2-N∆GR, respectively, followed by ligation and transformation of *Escherichia coli* Top 10 cells (Supplementary Table S3).

For MBD2 overexpression in mammalian cells (C2C12 and ES J1), the MBD2∆C coding sequence was amplified by PCR from peMBD2G (pc2399) using primer pairs containing HindIII (NEB, cat. no.: R3104S) and SalI (NEB, cat. no.: R3138S) restriction enzyme cutting sites for the forward primer and the reverse primer, respectively. Then, the amplified coding sequences were digested with HindIII/SalI and ligated into the HindIII/SalI digested pEGFP-N3 (pc714) vector to generate the pmMBD2∆C-G construct (Supplementary Fig. S1 and Supplementary Table S1). Q5 site-directed mutagenesis strategy was then adopted following the manufacturer’s protocol (NEB, cat. no.: E0554S) using the primer pairs listed in Supplementary Table S2 to generate the GR (glycine/arginine rich region) or CC (coiled coil region) depleted versions of MBD2: MBD2∆N∆CC, MBD2-C∆CC, MBD2∆C∆GR, and MBD2-N∆GR, followed by ligation and transformation of *E. coli* Top 10 cells (Supplementary Table S3). All plasmids used in this study were validated by DNA sequencing and the source references are listed in Supplementary Fig. S1 and Supplementary Table S1.

For mammalian expression of Hdac11 and Kat7, corresponding coding sequences were amplified from cDNA derived from ES cells. The coding sequences of Kat7 and Hdac11 were amplified and cloned into the pmRFP-C1 using the NEBuilder^®^ HiFi DNA Assembly kit (NEB, cat. no.: E2621S). For assembly, the pmRFP-C1 backbone was PCR-amplified with primers containing overhangs complementary to the Kat7 and Hdac11 inserts.

### Protein purification, labelling and analysis

MBD3 and MBD2 truncations fused with the carboxyl-terminal intein–chitin-binding domain (CBD) were expressed in BL21(DE3) *E. coli* cells (Supplementary Table S3) by incubating in Luira Bertani (LB) medium containing 0.5 mM isopropyl β-D-1-thiogalactopyranoside (IPTG) (Sigma–Aldrich, I6758-10G) at room temperature overnight incubation with shaking. Subsequently, the cell lysates were prepared by pelleting and resuspending the bacteria in lysis buffer (20 mM Tris–HCl, pH 8.5, 500 mM NaCl, 0.25% Triton X-100 and protease inhibitors phenylmethylsulfonyl fluoride (PMSF), AEBSF, E64, and pepstatin A), followed by sonication on ice and centrifugation at 15 000 rpm for 30 min. The cleared lysates were incubated with 2 ml chitin beads (NEB, S6651S) at 4°C with rotation for 3 h to allow CBD-chitin binding. Then beads were washed and treated in benzonase buffer (20 mM Tris–HCl, pH 8.5, 2 mM MgCl_2_, 20 mM NaCl, 0.1 mM PMSF) with benzonase (Merck, 70746-3, 1:2000 dilution) at 37°C for 90 min, followed by washing and treatment in DNase buffer (20 mM Tris–HCl, pH 8.5, 50 mM KCl, 2 mM MgCl_2_) with DNase I (4 µg/ml), RNase A (0.2 µg/µl) at 37°C for 30 min to remove DNA and RNA contaminants. Finally, proteins were eluted by cleavage at 4°C for two days in cleavage buffer (20 mM Tris–HCl, pH 8.5, 500 mM NaCl) with 50 mM dithiothreitol (DTT; Sigma–Aldrich, D9779-5G). The eluted fraction was concentrated using amicon^®^ ultra centrifugal filters with 10 kDa pore size and 4 ml sample volume (Merck, cat. no.: UFC8010), aliquoted, flash frozen, and stored at −80°C in storage buffer [20 mM Tris–HCl (Carl Roth, art. no.: 9090.5), pH 8.5, 300 mM NaCl (Carl Roth, art. no.: 0601.2)].

The concentrations of purified protein were determined using Pierce™ 660 nm protein assay kit (Thermo Fisher Scientific, cat. no.: 22660) following the manufacturer’s instruction with three replicates for both standard curve and samples. In brief, 10 µl of bovine serum albumin (BSA) standard (Thermo Fisher Scientific, cat. no.: 23208) proteins and storage buffer (blank) were mixed with 150 µl protein assay reagent in the 96-well micro test plate (Sarstedt, cat. no.: 82.1581.001) and incubated at room temperature for 5 min. The absorbance at 660 nm was measured using a plate reader Infinite 200 (Tecan). The blank-corrected absorbance was calculated by subtracting the average absorbance of the blank. The standard curve was generated by plotting the average blank-corrected absorbance for each BSA standard versus the relative concentrations. The protein concentrations were calculated according to the standard curve using the blank-corrected measurements.

Purified MBD2 proteins were fluorescently labelled using Alexa Fluor™ 546 NHS Ester (succinimidyl ester) (Invitrogen, cat. no.: A10237) following the manufacturer’s instructions with minor modifications. Briefly, in a 20 µl reaction solution, 100 µg of purified MBD2 protein was incubated with 1 mg unit size of Alexa Fluor™ 546 NHS Ester in a buffer containing 0.1 M sodium bicarbonate (pH 8.3) for 1 h at room temperature. The solution was then diluted into 500 µl with storage buffer [20 mM Tris–HCl (Carl Roth, art. no.: 9090.5), pH 8.5, 300 mM NaCl (Carl Roth, art. no.: 0601.2)], followed by dialysis and concentration using amicon^®^ ultra centrifugal filters (10 kDa pore size and 0.5 ml sample volume; Merck, cat. no.: UFC5010). Protein concentration was determined using the Pierce™ 660 nm protein assay kit (Thermo Fisher Scientific, cat. no.: 22660) following the manufacturer’s instructions. Aliquots were subsequently stored at −80°C under the same buffer conditions.

The purity of the proteins was analyzed by loading 2 µg and 10 µg purified protein separately onto a sodium dodecyl sulphate–polyacrylamide (SDS–PA) gel and 15% Tris–borate ethylenediaminetetraacetic acid (EDTA) polyacrylamide gel. The gels were stained with coomassie (to detect the proteins) and ethidium bromide (EtBr; to detect the potential contamination with nucleic acids) separately after electrophoresis. The SDS–PA gels after coomassie staining were captured by trans-illumination imaging using the Amersham Imager 600 (Supplementary Table S4) equipped with white light trans-illumination following the manufacturer’s instruction. The Tris–borate EDTA polyacrylamide gels after EtBr staining were imaged using the VWR genosmart (Supplementary Table S4) ultraviolet trans-illumination system.

### Synthesis of DNA templates for phase separation assay

The DNA used for the phase separation assay was synthesized by PCR (polymerase chain reaction) using Q5 polymerase (NEB, M0491S) as described before [21]. In brief, pUC18-MINX plasmid (Supplementary Table S1) was used as a template to amplify DNA using the primers listed in Supplementary Table S2. The 800 bp DNA with cytosine methylation was generated by replacing the dCTP with dmCTP, followed by Q5 polymerase-directed PCR [dATP (Invitrogen, cat. no.: 10216018), dTCP (Invitrogen, cat. no.: 18255018), dGTP (Invitrogen, cat. no.: 10218014), dmCTP (Jena Bioscience, cat. no.: NU-1125S)].

### Mouse brain nuclei isolation and fractionation

Mouse brains were collected from 3-month-old C57BL/6 mice (Charles River Laboratories, Inc., Sulzfeld, Germany) according to the animal care and use regulations (Government of Hessen, Darmstadt, Germany) and frozen in liquid nitrogen [4]. Five frozen mouse brains were crushed to powder and homogenized in 15 ml 0.25 M sucrose (cat. no.: 4661.2, Carl Roth) solution in buffer A [20 mM triethanolamine–HCl (pH 7.6) (cat. no.: T1377, Sigma–Aldrich Chemie GmbH (Merck), Munich, Germany], 30 mM KCl [cat. no.: H1758, Sigma–Aldrich Chemie GmbH (Merck)], 10 mM MgCl_2_ [cat. no.: M0250, Sigma–Aldrich Chemie GmbH (Merck)], 1 mM DTT [cat. no.: 04010, Sigma–Aldrich Chemie GmbH (Merck)], 1 mM PMSF (cat. no.: 6367.1, Carl Roth), followed by centrifugation at 1000 × *g* for 10 min. The pellet (containing the nuclei fraction) was resuspended in 30 ml 2.5 M sucrose buffer (2.5 M sucrose in buffer A). The raw nuclei fraction was pelleted by centrifugation for 30 min at 50 000 × *g* using an SW28 rotor (swinging bucket rotor SW28, Beckman Coulter, Brea, CA, USA). The pellet was resuspended in 20 ml 0.25 M sucrose buffer (0.25 M sucrose in buffer A) and centrifuged at 1000 × *g* for 10 min. The isolated nuclei were resuspended in phosphate-buffered saline (PBS) (137 mM NaCl, 2.7 mM KCl, 1 mM Na_2_HPO_4_ × 7H_2_O, 1 mM KH_2_PO_4_, pH 7.4), counted, and aliquoted into 1.5 ml eppendorf tubes. The nuclei were finally pelleted by centrifugation and the aliquoted nuclei pellets were stored at −20°C.

The chromatin was salt fractionated following a protocol from Teves and Henikoff [22] with modifications. All buffers during fractionation were precooled and supplemented with protease inhibitors [PMSF (cat. no.: 6367.1, Carl Roth, 1:100), PepA (P5318, Merck, 1:1000), E64 (AG-CP3-7006-M025, biomol GmbH, 1:100)], and phosphatase inhibitors [ethanolamine (398136, Merck, 1:1000)] before use and all steps were done on ice. A total of 10^8^ nuclei were thawed and resuspended in 10 ml buffer A (15 mM Tris, pH 8.0, 15 mM NaCl, 60 mM KCl, 1 mM EDTA, pH 8.0, 0.5 mM ethylene glycol bis(β-aminoethylether) tetraacetic acid (EGTA), pH 8) and pelleted at 400 × *g* for 5 min. After 5 min incubation, the supernateant containing residual cytoplasm (CP) was transferred to new tubes. Nucleoplasm (NP) fraction was extracted by resuspending the nuclei pellet in 10 ml isotonic buffer (10 mM Tris, pH 8.0, 15 mM NaCl, 60 mM KCl, 1.5 mM EDTA, pH 8.0) and incubating at 4°C for 20 min, followed by centrifugation at 500 × *g* for 5 min. The euchromatin (EC) fraction was extracted by resuspending the pellets in 10 ml euchromatin extraction buffer (10 mM, Tris, pH 8.0, 250 mM NaCl, 1 mM EDTA, pH 8.0) and incubating at 4°C for 20 min followed by centrifugation at 1000 × *g* for 5 min. Finally, the heterochromatin (HC) fraction was extracted by resuspending the pellets in 10 ml heterochromatin extraction buffer (10 mM Tris, pH 8.0, 600 mM NaCl, 1 mM EDTA, pH 8.0) and incubating at 4°C for 20 min. The insoluble fraction (P) was pelleted by centrifugation at 20 000 × *g* for 10 min and dissolved in 10 ml 2% sodium dodecyl sulphate (SDS) followed by sonication (75% power, 30 s sonication with 1.5 min incubation for three cycles). The CP, NP, EC, and HC were concentrated using Amicon Ultra Centrifugal Filter 10 kDa and quantified using Pierce™ 660 nm protein assay kit following the aforementioned procedure.

### *In vitro* phase separation and detection

For *in vitro* phase separation, proteins were firstly thawed on ice and centrifuged at 14 000 rpm, 4°C for 10 min to remove all aggregates. Then, phase separation in solutions (20 mM Tris–HCl, pH 8.5) with various concentrations of salt, protein, crowding agents, and DNA was achieved by incubating for 45 min at room temperature.

To check the droplet morphology, phase separation samples were loaded onto chambers made of double-sided tapes and sealed with coverslips. Fluorescence and differential interference contrast (DIC) images were taken using a Nikon Eclipse TiE2 microscope equipped with a Plan Apo λ 40× air objective. All images were processed and analyzed using FIJI (https://imagej.net/software/fiji/). To measure the protein distributions after *in vitro* phase separation, the phase separation samples were sedimented by centrifugation at 14 000 rpm for 15 min at room temperature. The supernatants and pellets were separated and applied to a 12% SDS–PA gel, which was stained with Coomassie for 1–2 h after electrophoresis and subsequently washed with destaining buffer (100 ml acetic acid, 100 ml ethanol, and 500 ml H_2_O) overnight. The coomassie signal was detected using an Amersham Imager system (Supplementary Table S4). The protein fractions in pellets were quantitatively analyzed using FIJI, and plotted using GraphPad Prism (https://www.graphpad.com/scientific-software/prism/).

The turbidity assay was also applied to check the phase separation properties of MBD2 proteins. In brief, 20 µl phase separation solutions with various conditions were prepared and incubated at room temperature for 45 min in a 384-well plate with an optically clear bottom (PerkinElmer, 6007550), followed by absorbance measurement at 340 nm at room temperature using a plate reader Infinite 200 system (Tecan).

### Sample preparation for mass spectrometry analysis

Mass spectrometry sample preparation was done as described before [6]. Following heterochromatin isolation, *in vitro* phase separation and sedimentation, protein samples were prepared, and protein aggregation capture was employed to remove detergents and salts from the samples. In this procedure, 1000 µg of Sera-Mag™ beads (1 mg, GE24152105050250, Sigma) were used for each 100 µg of chromatin fraction and washed three times with 70% acetonitrile (100030, Merck). After the last wash, 300 µl of the chromatin solution, corresponding to 100 µg, was mixed with the beads, and 700 µl of 100% acetonitrile was added to each sample. The chromatin-bead mixtures were vortexed, incubated at room temperature for 10 min, vortexed again, and allowed to settle. The samples were then placed in a magnetic rack and sequentially washed with 700 µl of 100% acetonitrile, 1 ml of 95% acetonitrile, and 1 ml of 70% ethanol. After evaporating the remaining ethanol, the beads were resuspended in 400 µl of 50 mM HEPES (pH 8.5) containing freshly added 5 mM tris(2-carboxyethyl)phosphin hydrochlorid (C4706, Sigma–Aldrich) and 5.5 mM 2-chloroacetamide (CAA, C4706, Sigma–Aldrich). The samples were incubated at room temperature for 30 min, after which LysC (1:200, 125-05061, FUJIFILM Wako) and trypsin (1:100, 90057, Thermo) were added. The proteins were digested overnight at 37°C. To halt protease activity, 1% (v/v) trifluoroacetic acid (TFA, T6508, Sigma–Aldrich) was added the following day, and the samples were loaded onto custom-made StageTips containing three layers of polystyrene-divinylbenzene, reversed-phase sulfonate (SDB-RPS) matrix (66886-U, Empore) pre-equilibrated with 0.1% (v/v) TFA. After loading, two washes with 0.1% (v/v) TFA were carried out, and peptides were eluted using 80% acetonitrile and 2% ammonium hydroxide (105428, Supelco). Once the eluates were evaporated in a SpeedVac (Eppendorf) centrifuge, the samples were reconstituted in 20 µl of 0.1% TFA and 2% acetonitrile. After solubilizing the peptides by shaking continuously for 10 min at 2000 rpm, peptide concentrations were estimated using a NanoDrop™ 2000 spectrophotometer (Thermo Fisher Scientific) at 280 nm.

### Nanoflow liquid chromatography-mass spectrometry/mass spectrometry (LC-MS/MS) measurements for different nuclei fractions

Peptides were separated via liquid chromatography on an Easy-nLC 1200 (Thermo Fisher Scientific) (Supplementary Table S6) using in-house packed 50 cm columns of ReproSil-Pur C18-AQ 1.9 µm resin (Dr Maisch GmbH). A binary buffer system (buffer A: 0.1% formic acid; buffer B: 0.1% formic acid and 80% acetonitrile) was used, with a progressively increasing buffer B percentage (starting at 5% and ending at 95%) to elute peptides over 120 min at a constant flow rate of 300 nl/min. The peptides were then injected into an Orbitrap Exploris™ 480 mass spectrometer (Thermo Fisher Scientific) (Supplementary Table S6) through a nanoelectrospray source. The samples were run in duplicates, followed by a washing step, while maintaining a constant column temperature of 60°C. Real-time monitoring of operational parameters was achieved using SprayQc (a real-time LC-MS/MS quality monitoring system). Measurements were performed in data-independent acquisition (DIA) mode. The orbitrap resolution for full scans was set at 120 000 within a 350–1400 m/z range, with a maximum injection time (IT) of 45 ms. For DIA scans, the mass range was set to 361–1033, with isolation windows of 22.4 m/z and a default window overlap of 1 m/z. The orbitrap resolution for DIA scans was set at 30 000, the normalized automatic gain control target at 1000%, and the maximum IT at 54 ms.

### Data-independent acquisition mass spectrometry data quantification

DIA raw data processing was performed by the software DIA-NN 1.7.17 beta 12 (Supplementary Table S6) in ‘high accuracy’ mode. Instead of a previously measured precursor library, spectra, and retention times (RTs) were predicted by a deep learning-based algorithm and spectral libraries were generated from FASTA files. Cross-run normalization was established in an RT-dependent manner. Missed cleavages were set to 1. N-terminal methionine excision was activated and cysteine carbamidomethylation was set as a fixed modification. Proteins were grouped with the additional command ‘–relaxed- prot-inf’. Match-between runs was enabled and the precursor false discovery rate (FDR) was set to 1%.

Downstream analysis of DIA raw data output was performed with Perseus (version 1.6.0.9) [23]. Proteins identified in less than three samples were filtered out. The filtered data was then processed [log_2_() transformation, normalization against the median intensity of each measurement] before dot plot and venn diagram generation, which were done using GraphPad Prism. The GO and subcellular localization information was added by merging the data with mouse proteomic data from UniProt.

Fold changes of NuRD members were calculated after raw data processing [log_2_() transformation, imputation, and normalization against the median intensity of each measurement] in the pellet proteome with or without additional MBD2 condensates. Unpaired *t*-tests were performed to obtain significantly influenced NuRD members in heterochromatin pellets by additional MBD2 condensates using GraphPad Prism. *P*-values below 0.05 indicate significant changes.

### Datasets of IDR and phase separation scaffold proteins

Key resources are listed in Supplementary Table S7.

Known intrinsically disordered proteins (IDRs) in *Mus musculus* and *Homo sapiens* were collected from DisProt (https://www.disprot.org/) [24]. Published phase separation scaffold proteins in mice (324) were collected from PhaSepDB [25], LLPSDB [26], and PhaSePro [27]. The mouse proteome containing 17 224 reviewed proteins was downloaded from Uniprot including gene and protein names, amino acid sequences, subcellular localization, and gene ontology (GO) (https://www.uniprot.org/) [28].

The prediction of phase separation proteins in the mouse proteome was done using DrLLPS [18], PSAP [17], and PhaSePred [16]. For DrLLPS, the candidate phase separation proteins in mice were downloaded directly [18]. For the PSAP, the phase separation scores of the whole mouse proteome were predicted using the scripts from [17]. The top 1000 proteins were considered. For the PhaSePred datasets, the phase separation score of the whole mouse proteome was downloaded and the top 1000 proteins that possibly self-assemble or partner-dependent assemble to form condensates were considered [16]. If the proteins were predicted with high self-assemble and high partner-dependent-assemble, they were considered to preferentially undergo self-assembling phase separation (SaPS).

The prediction data from the three predictors (DrLLPS, PSAP, and PhaSePred) were integrated and proteins predicted by at least two predictors were considered candidate phase separation scaffold proteins. Combining these with published phase separation scaffold proteins resulted in a list of all potential phase separation scaffold proteins, of which, the proteins located in the nucleus and subnuclear compartments were further recognized based on their subcellular localization or GO terms from UniProt.

### Identification of potential phase separation scaffold proteins at pericentric heterochromatin

The subnuclei localization and protein family of candidate phase separation scaffold proteins was filtered based on the keywords in protein description, subcellular localization and GO terms. The keywords used were: (i) pericentric heterochromatin: heterochromatin, methylated chromatin, methylated DNA, pericentromeric, pericentric, chromocenter, condensed; (ii) euchromatin: euchromatin, transcriptionally active, active transcription, transcriptional enhancer; (iii) nucleus speckle: nuclear speckle, nucleus speckle; (iv) nucleolus: nucleolus; (v) nuclear pore: nuclear pore; (vi) DNA damage: DNA damage; (vii) inactive X chromosome: inactive X; (viii) paraspeckle: paraspeckle; (ix) germ granule: germ granule; (x) Cajal: cajal; (xi) PML: PML; (xii) methyl-CpG binding domain (MBD) and cbx: MBD, MeCP, cbx5; (xiii) histone deacetylase: histone deacetylase; (xiv) histone acetylase: histone acetyltransferase, histone deacetylase corepressor; (xv) NuRD: NuRD, Gatad.

The potential phase separation scaffold proteins in pericentric heterochromatin were recognized by integrating the datasets from phase separation predictors, pericentric heterochromatin proteome, and heterochromatin phase separation (HC_P) proteome. The pericentric heterochromatin proteomic data was acquired by reanalyzing the pericentric heterochromatin proteomic data in mouse brain from [4], focusing on proteins consistently detected across all three replicates (pericentric heterochromatin proteome). Then, the proteins present in pericentric heterochromatin proteome, HC_P proteomic data and predicted pericentric heterochromatin phase separation scaffold proteins (PCH prediction) were considered potential phase separation scaffold proteins.

### Native mass spectrometry

Protein samples of MBD2∆N and MBD2∆N∆CC were thawed on ice and centrifuged for 5 min at 17 000 × *g*. The supernatant was diluted to a protein concentration of 32 µM in 200 mM ammonium acetate (Sigma, CAS cat. no.: 631-61-8) and 5 µl of the solution was loaded into glass needle emitters produced in-house with a Sutter P97 needle puller (Sutter Instrument, One Digital Drive Novato, CA 94949, USA) (Supplementary Table S6). The protein solution was then electrosprayed into a Waters Synapt XS ion mobility-mass spectrometer (Waters Corporation, 34 Maple Street Milford, MA) in positive ion mode. A capillary voltage of 1.5 kV, a source temperature of 30°C, and a sampling cone voltage of 30 V were used. The spectra were processed by smoothing two times over 50 channels using a Savitzky–Golay filter in MassLynx V4.2 software (Waters corporation). The mass of MBD2 variant monomers and oligomers were calculated based on the charge states and m/z ranges using MassLynx V4.2 software.

### Prediction of protein structures and key residues for phase separation

The structures of MBD2 monomers and oligomers were predicted by Alphafold Server [29] (Supplementary Table S7). The MBD2 phase separation key residues at the amino terminus or the carboxyl terminus were predicted by PSPHunter [30] (Supplementary Table S7).

### Mammalian cell culture and transfection

C2C12 mouse myoblast cells (Supplementary Table S3) were cultured in Dulbecco’s modified Eagle medium (DMEM) high glucose (Sigma–Aldrich Chemie GmbH, cat. no.: D6429) supplemented with 20% fetal calf serum, 1× L-glutamine (Sigma–Aldrich Chemie GmbH, G7513), and 1 µM gentamicin (Sigma–Aldrich Chemie GmbH, cat. no.: G1397). The transfection was performed using a Neon Transfection System (Thermofisher) following the manufacturer’s instructions. Cells were seeded onto 35 mm plates with a glass bottom for live cell experiments or cell culture dishes with glass coverslips (Paul Marienfeld GmbH & Co. KG, cat. no.: 0111520) for fixed cell experiments.

The mouse embryonic stem cells (mESC) J1 cells were grown in DMEM high glucose (Sigma–Aldrich Chemie GmbH, cat. no.:D6429) supplemented with 15% fetal calf serum, 1 × nonessential amino acids [Sigma–Aldrich Chemie GmbH, cat. no.: M7145), 1 × penicillin/streptomycin (pen/strep) (Sigma–Aldrich Chemie GmbH, cat. no.: P4333), 1 × L-glutamine (Sigma–Aldrich Chemie GmbH, cat. no.: G7513), 0.1 mM beta-mercaptoethanol (Carl Roth, cat. no.: 4227), 1000 U/ml recombinant mouse LIF (Millipore), and 2i [1 M PD032591 and 3 M CHIR99021 (Axon Medchem, cat. nos.: 1408 and 1386 respectively)] on gelatin-coated culture dishes (0.2% gelatin; Sigma–Aldrich Chemie GmbH, cat. no.: G1393) or laminin-coated coverslips (10 µg/ml laminin; Th. Geyer GmbH & Co. KG, cat. no.: L2020-1MG).

### Generation of MBD2 knockout cells

The guide RNA (gRNA) was designed using Benchling (https://www.benchling.com/). One gRNA (5′-CATCCTCTTCCCGCTCTCCG-3′) targeting the second start codon of MBD2 was selected and cloned into pSpCas9(BB)-2A-Puro (PX459) (pc3926, Addgene: 48139). The repair template containing mRFP-SV40 poly(A), flanked by MBD2 sequencing targeting the CRISPR/Cas9 cutting site, was generated by inserting the MBD2 sequences downstream of the second start codon into the plasmid pFB-MBD2.1-mRFP (pc2391).

For MBD2 knockout, the Neon electroporation system (Thermo Fisher Scientific) was used to deliver 5 µg CRISPR/Cas9 and 20 µg repair template DNA into 5 × 10^6^ ES J1 cells. Electroporated cells were seeded onto gelatin-coated 100-mm dishes with ES cell culture medium. Twenty-four hours after transfection, 1 µg/ml puromycin was added to the medium for 48 h to enrich transfected cells. Following selection, cells were trypsinized into a single-cell suspension, and 5 × 10^5^ cells were replated onto new gelatin-coated 100-mm dishes. After 1 week of growth, individual colonies were manually picked and transferred into gelatin-coated 96-well plates for expansion. Colonies exhibiting red fluorescent protein (RFP) fluorescence were selected for further validation by PCR, western blotting, and immunofluorescence.

### Quantification of endogenous MBD2 abundance

Nuclei were prepared as previously described by McKittrick *et al.* (2004) [31] with modifications. In brief, ES and C2C12 cell pellets were resuspended in cold TM2 buffer [10 mM Tris, pH 7.5, 2 mM MgCl_2_, protease inhibitors (PMSF, AEBSF, E64 and pepstatin A)] with gentle vortexing, held on ice for 1 min, and 10% NP-40 was added to a final concentration of 1.5% with gentle vortexing. After 5 min on ice, crude nuclei were pelleted by centrifuge at 800 rpm, 4°C, 5 min. Pellet was washed once by adding 1 ml TM2 buffer and centrifuged at 1000 rpm, 4°C, 5 min. Pellets were resuspended in PBS with protease inhibitors (PMSF, AEBSF, E64, and pepstatin A) and nuclei number counted. Nuclei were centrifuged at 1000 rpm, 4°C, 5 min and pellets resuspended with modified PARP buffer (0.025 M Tris, pH 8, 1 M NaCl, 0.05 M glucose, 0.2% Tween 20, 0.2% NP-40 substitutive, 2 mM MgCl_2_) together with protease inhibitors and 100 U/ml benzonase (30 µl PARP per 1 × 10^6^ nuclei). Proteins were released by DNA digestion at 4°C with vortexing for 1 h and the concentration determined using Pierce™ 660 nm protein assay kit as mentioned before. Extracts from 5 × 10^5^ (C2C12) and 1 × 10^6^ (ES) nuclei were loaded onto an sodium dodecyl sulphate–polyacrylamide gel electrophoresis (SDS–PAGE) gel together with purified MBD2 proteins (1, 5, 10, 20, and 30 ng), transferred to a nitrocellulose membrane and the membrane was blocked with 5% low-fat milk (in PBS) for 1 h at room temperature, followed by primary antibody incubation (anti-MBD2, RA-18) at 4°C overnight, washing, and secondary antibody incubation (anti-rabbit IgG Cy5) for 1 h (Supplementary Table S5). Fluorescent signals were detected using Amersham Imager (Supplementary Table S4). The intensities (gray values) of protein bands were measured using ImageJ and plotted using Excel. Protein abundances were calculated according to the linear trendline.

To estimate the MBD2 abundance in mouse tissue, MBD2 levels in C2C12 (myoblast cells from mouse muscle) cells and mouse muscle were assumed to be comparable. Thus, physiological MBD2 levels in other tissues and mean/median abundance across tissues were calculated according to their relative abundance (ppi) to that in muscle.

### Co-immunoprecipitation and western blot analysis

We performed co-immunoprecipitation experiments as described in [32–34] to analyze protein-protein interactions between MBD2 isoforms and Hdac11 and Kat7. HEK cells were co-transfected with MBD2 proteins and Hdac11/Kat7 expression vectors using PEI (polyethyleneimine, pH 7.0, cat. no.: 40827–7, Sigma–Aldrich Chemie GmbH, Steinheim, Germany) as previously described [35]. For 10 cm diameter dishes, 90 µl PEI and 30 µg plasmid DNA were added to 900 µl DMEM without supplements and mixed by vortexing. The mixtures of PEI and DNA were combined, vortexed for 80 s, and incubated at room temperature for 30 min to 1 h. Then, the mixture was added dropwise to the cells and harvested by washing 48 h after transfection. Cell suspensions were centrifuged for 5 min at 2000 rpm and 4°C. The pellets were resuspended in 300 ice-cold lysis buffer containing 20 mM Tris–HCl (pH 8), 250 mM NaCl, 1.5 mM MgCl_2_, 0.4% NP-40, 0.2 mM EDTA, and protease inhibitors 1 mM AEBSF [4-(2-aminoethyl) benzyl sulfonyl fluoride hydrochloride, cat. no.: A1421.0100, VWR, Radnor, PA, USA], 1 mM E64 (cat. no.: E3132, Sigma–Aldrich, St Louis, MO, USA), 1 nM Pepstatin A (cat. no.: 77170, Sigma–Aldrich, St Louis, MO, USA), PMSF (10 µM, Sigma–Aldrich, St. Louis, MO, USA/Solarbio; catalog #P8340) and AEBSF (1 mM, AppliChem, Darmstadt, Germany). Cell lysis was performed with a syringe using 21 G needles and 25 strokes per sample, while maintained on ice. After homogenization and incubation on ice for 25 min, cell lysates were cleared by centrifugation (15 min at 13 000 × *g* and 4°C). For the input fraction, 80 μl of the lysate was taken apart. For the binding fraction, the remaining lysate was incubated with green fluorescent protein (GFP)-binder beads [36] on rotation at 4°C for 2 h. Then, beads were washed four times to remove nonbound proteins with 600 µl of washing buffer containing 20 mM Tris–HCl, 150 mM NaCl (pH 8), 1.5 mM MgCl_2_, and 0.2 mM EDTA. Washing was performed by centrifugation of the samples at 2000 rpm, followed by supernatant removal. After washing, beads were resuspended in a small volume of loading buffer 4× SDS (400 mM DTT, 200 mM Tris–HCl, pH 6.8, 8% SDS, 0.4% bromophenol blue, and 40% glycerol). Both input and bound fractions were boiled at 95°C and separated on 8% SDS–PA gels. The SDS–PAGE and western blotting experiments were performed as in [37]. Four microlitres of protein ladder maker were loaded into the polyacrylamide gel (MWP06 BlueEasy Prestained Protein Marker, Nippongenetics). For visualization of the bands, horseradish peroxidase conjugated secondary antibodies were used. All the characteristics and dilutions of primary and secondary antibodies and dilutions used are described in Supplementary Table S5. To develop the membranes, Pierce™ ECL Western Blotting Substrate was used (cat. no.: 32209, Thermo Fisher Scientific, Waltham, MA, USA). The Amersham AI600 Imager with a CCD camera (GE Healthcare, Chicago, II, USA) was used to image immunoreactive bands. Unprocessed scans for all the blots are provided with the data sets uploaded to TUdatalib.

To analyze the efficiency of the nuclei fractionation method, the CP, NP, EC, HC, and P fractions from equal amounts of nuclei (the same volume as all fractions were extracted with the same volume of buffer) were used for western blot analysis. Laemmli buffer was added to all samples with a final 1× Laemmli buffer [2% SDS (cat. no.: 2326.2, Carl Roth), 50 mM Tris (pH 6.8), 10% glycerol (cat. no.: 0798.3, Carl Roth), 0.01% bromophenol blue (cat. no.: A512.1, Carl Roth), 100 mM DTT], followed by boiling at 95°C for 5 min. After electrophoresis, proteins were transferred to nitrocellulose membranes using a trans-blot^®^ turbo™ transfer system (1704150, Bio-Rad) at 25 V for 60 min. The membranes were stained with Ponceau S solution (cat. no.: P7170, Sigma–Aldrich) to check for transfer efficiency. The membranes were then blocked with 5% low-fat milk in PBS for 30 min and incubated with mouse anti-lamin B (61047C, Progen Biotechnik GmbH, 1:10), rabbit anti-beta III tubulin (ab52623, Abcam, 1:1000), mouse anti-H1 (sc-8030, Santa Cruz Biotechnology, 4 µg/ml), rat anti-MeCP2 (4H7, self-made, undiluted) [38], and mouse anti-HP1a (MAB3584, Active Motif SA, 1:500) (Supplementary Table S5) in 5% low-fat milk in PBS overnight at 4°C. The membranes were washed three times with 0.05% PBST (0.05% Tween 20 in PBS), incubated with secondary antibodies in 5% low-fat milk for one h (anti-mouse IgG Cy5 (JIM-715-175-150, Jackson ImmunoResearch Europe Ltd., 1:500), anti-rabbit IgG Cy5 (715-175-152, Jackson ImmunoResearch Europe Ltd., 1:1000), and anti-rat IgG Cy3 [JIM-712-165-153, Jackson ImmunoResearch Europe Ltd., 1:1000)] (Supplementary Table S5), and washed again three times with PBST. The fluorescence was detected using an Amersham AI600 imager (Supplementary Table S4).

### Immunofluorescence staining and imaging

Transfected C2C12 cells were fixed 36 h after transfection with 3.7% formaldehyde in PBS for 15 min, washed three times using PBST (0.02% Tween 20 in PBS), permeabilized using 0.5% Triton X-100 in PBS for 10 min, washed three times using 0.02% PBST, and blocked in 4% BSA in 1× PBS for 1 h at room temperature. GATAD2b and H3K27ac were detected using rabbit anti-GATAD2b (Invitrogen, PA5-53536) and rabbit anti-acetyl-histone H3 (Lys27) (Cell signalling Technology, D5E4) antibodies separately for 60 min at room temperature in 4% BSA, followed by three times washing with PBST. Cells were then incubated with the secondary antibody donkey anti-rabbit IgG Cy5 (Jackson ImmunoResearch Europe Ltd., 711-175-152) (Supplementary Table S5) for 60 min at room temperature and washed with PBST three times. DNA was counterstained with 4,6-diamidine-2-phenylindole dihydrochloride (DAPI, 1 g/ml) for 6 min, followed by three times washing using PBST and one time washing using H_2_O. Cells were finally kept in mounting media and stored at −20°C until use.

2D images were taken using Leica TCS SP5 II confocal microscope with a HCX PL APO 100×/1.44 oil Corr CS objective or Nikon Eclipse TiE2 microscope equipped with a Plan Apo λ 40× air objective (Supplementary Table S4). For 3D images, cells with comparable GFP levels were imaged using Leica TCS SP5 II confocal microscope with a HCX PL APO 100×/1.44 oil Corr CS objective.

### Quantitative image analysis

2D images were quantitatively analyzed using FIJI. The cell nuclei and pericentric heterochromatin compartments were segmented based on the DAPI intensities. Cells were subgrouped into low, middle, and high ectopic expression levels of MBD2 constructs based on the GFP intensities. Nuclei and heterochromatin compartment parameters (size, number, fluorescence intensities, etc.) were measured. The fraction of DNA in pericentric heterochromatin was calculated as the percent ratio of total DAPI intensity in pericentric heterochromatin (PCH) (SPCH × n × IPCH) to total DAPI intensity in the whole nucleus (SN × IN). The percent of MBD2 constructs in pericentric heterochromatin was calculated as the percent ratio of total MBD2 intensities in pericentric heterochromatin (SPCH × n × IPCH) to that in the whole nucleus (SN × IN). Significances were calculated by unpaired *t*-test in GraphPad Prism. ns *P* >.05; *P ≤.05; **P ≤.01; ***P ≤.001. Scatter plots represent the mean ± standard deviation (SD), as well as statistical significance and were generated in GraphPad Prism.

3D images were analyzed using FIJI and Rstudio (https://posit.co/) as described previously [39]. Nuclei were segmented in FIJI and classified into seven compaction classes in Rstudio based on the DAPI channel. The percentage of DAPI in each class was calculated using Rstudio. The bar plot was generated and significance was calculated by unpaired *t*-test in GraphPad Prism. ns *P* >.05; *P ≤.05; **P ≤.01; ***P ≤.001. Data are presented as mean ± SD.

### Fluorescence recovery after half photobleaching

C2C12 transfected cells with similar expression levels in the bleached region and similar pericentric heterochromatin sizes were selected for fluorescence recovery after half photobleaching (half-FRAP) assays. Twenty-four hours after transfection, live cells were transferred onto a prewarmed Leica TCS SP5 II confocal microscope with a HCX PL APO 100×/1.44 oil Corr CS objective (Supplementary Table S4). The half-FRAP assay was performed at 37°C. For excitation of GFP, the 488 nm argon ion laser was applied at 13% power and the emission was detected with a 490–560 nm filter. The settings for scanning were 400 Hz, 10× zoom, image format 256 × 256 pixels, pinhole 95.55 μm. The photobleaching of half of a pericentric heterochromatin compartment was obtained by 100% 488 nm argon ion laser power. The changes in fluorescence signal in both bleached and nonbleached half of the compartment were tracked with nine images taken before and 130 taken after photobleaching at an interval of ∼1.3 s.

Ectopic expression levels of MBD2 constructs in imaged live cells were estimated using a standard curve as described by Zhang *et al.* (2022) [11]. In brief, gradient solutions of purified GFP-MeCP2R168X [11] protein were loaded onto a chamber made of double-sided tape and sealed with coverslips. The images were taken using the same microscope applied for half-FRAP with the same settings. The mean fluorescence intensities of homogeneous GFP-MeCP2R168X solutions were measured using ImageJ and plotted versus the corresponding known protein concentrations to generate the standard curves. The ectopic MBD2 concentrations in nuclei were calculated, and the cells with MBD2 concentrations from 15 to 35 µM were chosen for further analysis.

The selected half-FRAP images were analyzed, and dip values were calculated using the released script from [40] (Supplementary Table S7). The normalized fluorescence in both bleached and nonbleached half at all time points of each independent experiment were downloaded after analysis. The curves of each MBD2 construct were generated using GraphPad Prism showing the mean ± SD. The dip values were plotted and unpaired *t*-tests were conducted using GraphPad Prism. ns *P* >.05; **P ≤.01; ***P ≤.001.

### Fluorescence loss in photobleaching

The experiments were performed using a Leica SP5-II confocal microscope (Supplementary Table S4). Scanning was set to 256 × 64 pixels at 1000 Hz for a pixel size of 0.20 µm/pixel and 0.15 s exposure time in a argon 488 nm laser. Samples were scanned for cells with different MBD2 levels (see above). Then, a bleach circle with ∼1 µm radius was defined either outside of the nucleus (for negative) or between heterochromatin compartments. After a first frame of pre-bleach, a sequence of 150 frames (22.5 s) was acquired at 10–20% of laser power while the bleach area was receiving a 100% laser power.

For the analysis, the bleached area was segmented and several 3 × 3 pixels region of interest were placed either in heterochromatin or nucleoplasm compartments. The mean intensity was measured and normalized to the starting intensity in each region. For each condition, 12–20 cells from two biological replicates were measured and averaged to produce the final graphs. In addition to the average, a 95% confidence interval was calculated to show the variability of the regions within the same condition.

## Results

### The heterochromatin fraction phase separates *in vitro*

Previous work reported phase separation (PS) as a potential underlying mechanism modulating heterochromatin compartmentalization, including pericentric heterochromatin (PCH) [9–11] and inactive X chromosome (Xi) [7, 41]. However, the complex and distinct assemblies of proteins involved in different heterochromatin compartments [4] and their respective phase separation properties remain largely unknown. Given the challenges of exploring the phase separation properties and functions of all heterochromatin-associated proteins one by one, we started by investigating the phase separation properties of heterochromatin fractions through *in vitro* phase separation assays following heterochromatin protein isolation.

**Figure 1.**
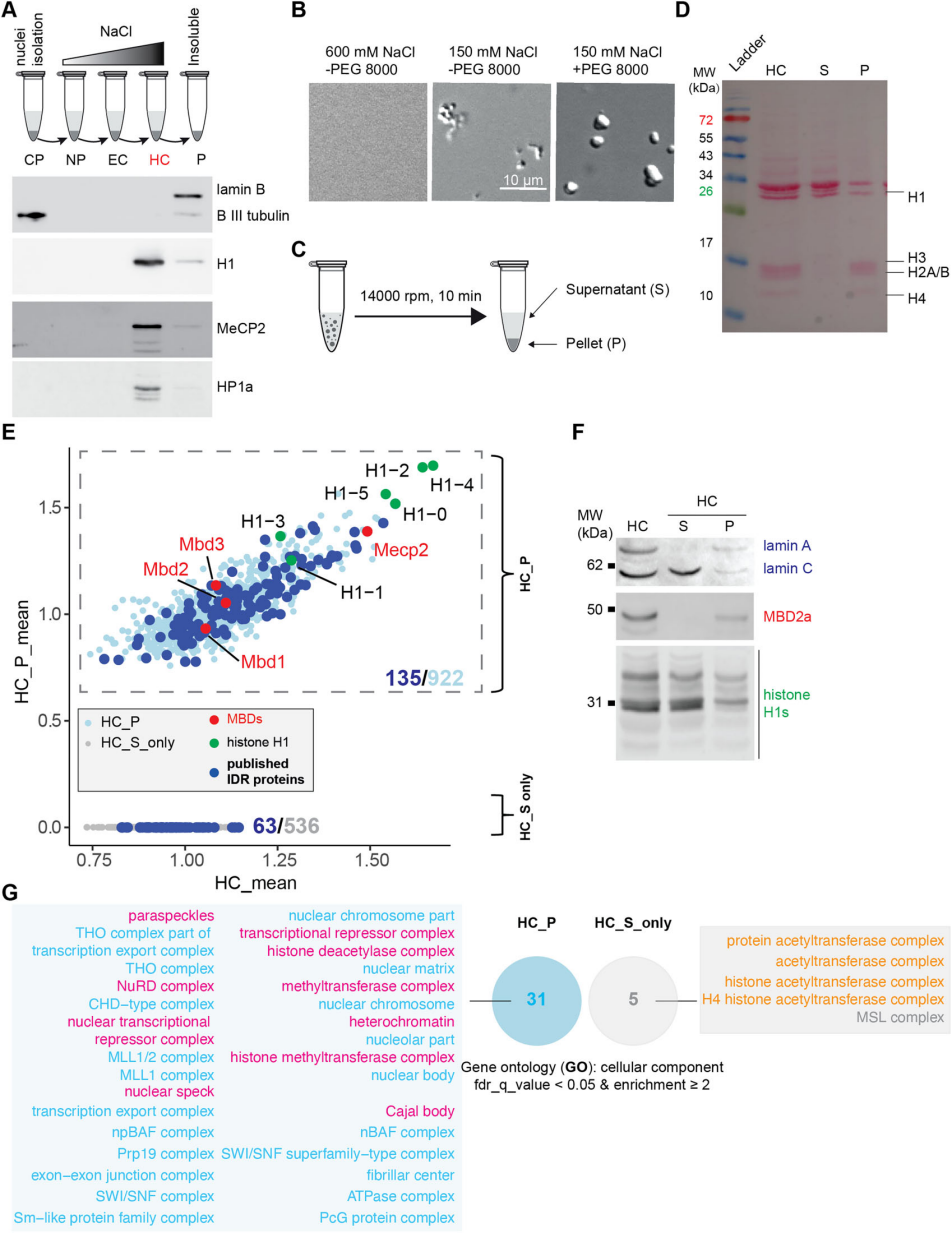
Mouse brain heterochromatin fraction phase separates *in vitro*. **(A)** Subnuclear fractionation (top) and corresponding validation (bottom). (top) Workflow of nuclear isolation from the cell pellet and salt-gradient mediated subnuclear fractionation. Free proteins in nucleoplasm (NP) were first eluted, followed by those bound to euchromatin (EC) and heterochromatin (HC), leaving the insoluble structure proteins in the pellet (P) with increased salt concentration. (bottom) Western blot detection of marker proteins for cytoplasm (beta III tubulin), heterochromatin (HP1 alpha, MeCP2 and histone H1) and insoluble nuclear proteins (lamin B). Full blots are shown in Supplementary Fig. S2A. CP: cytoplasm. **(B)** DIC images of heterochromatin condensates following *in vitro* phase separation with/without the crowding agent PEG 8000 in a buffer containing 150 mM NaCl. *n* = three replicates. **(C)** Schematic graph of droplet sedimentation assay after *in vitro* phase separation and the samples used for data-independent acquisition mass spectrometry (DIA-MS) analysis. **(D)** Ponceau staining of total proteins after SDS–PAGE analysis. Samples from heterochromatin (HC), supernatant (S), and pellet (P) as shown in panels (A)–(C) were loaded. **(E)** Dot plot of protein mean intensities in heterochromatin pellets (condensates) (HC_P) versus those in the (total) heterochromatin fractions. Only proteins detected in ≥2 replicates were considered in each group and the mean intensities were calculated. Gray dots: Proteins not found in HC_P. Light blue dots: Proteins found in HC_P. Dark blue dots: Published intrinsically disordered (IDR) proteins found in heterochromatin fraction. Green dots: MBD family proteins. Red dots: Histone H1 variants. *n* = three replicates. **(F)** Western blot detection of protein distribution in condensates (P) and supernatant (S). Full blots were shown in Supplementary Fig. S2B. **(G)** GO analysis of proteins recognized (HC_P) and unrecognized (HC_S only) in pelleted HC condensates. The protein list was subjected to the GOrilla tool [4] for GO analysis in the category of cellular component. The proteins recognized in the whole nuclei were applied as the background list. GO terms with a FDR q-value ≤0.05 and enrichment ≥2 were considered. The GO terms for cytoplasmic, RNA, ribosome, and nuclear membrane were removed manually.

We isolated the heterochromatin proteins from mouse brains using nuclei isolation [4] and salt gradient-mediated nuclei fractionation [5, 22] (Fig. 1A top). This enriched the nuclei proteins into four subnuclear fractions: (i) freely diffusing proteins in the nucleoplasm (NP), (ii) proteins bound to highly accessible euchromatin (EC), (iii) proteins bound to the highly compacted heterochromatin (HC), and (iv) pelleted insoluble structural proteins (P) (Fig. 1A). Western blot analysis confirmed the enrichment of heterochromatin-related proteins into the heterochromatin fraction, including MeCP2 and HP1a, two well-known heterochromatin markers with reported phase separation properties (Fig. 1A, bottom, and Supplementary Fig. S2A) [9–11]. Then, the heterochromatin fraction was subjected to *in vitro* phase separation. By reducing the salt concentration, the soluble heterochromatin proteins underwent phase separation, forming irregular aggregates that transitioned into more spherical condensates with smoother boundaries in the presence of crowding agents (PEG 8000) (Fig. 1B), indicating the broad phase separation capacity of heterochromatin proteins. The condensates were further collected by centrifugation (Fig. 1C). Proteins in the supernatant (S) and pellet (P) were different in both abundance and composition as shown by Ponceau staining (Fig. 1D). This indicates that only a subset of heterochromatin proteins are directly involved in heterochromatin phase separation.

To identify the heterochromatin phase separation-associated proteins, we performed mass spectrometry (MS) analysis. The whole nuclear extract, the heterochromatin fraction, and both supernatant and pellet from the *in vitro* heterochromatin phase separation assay were analyzed using DIA-MS [6]. The DIA-MS data from three replicates were analyzed following the pipeline described in Supplementary Fig. S3A. Approximately 4000 and 1900 proteins were detected in the whole nuclei and heterochromatin fraction, respectively, with low variation between replicates (Supplementary Fig. S3B and C and Supplementary Table S8). Around one thousand proteins were detected in the phase-separated condensates (pellets), representing around half of the heterochromatin proteins (Supplementary Fig. S3B). Given the boundary effect inherent to phase-separated condensates, which selectively recruit cofactors via multivalent weak interactions while excluding other factors, we compared the protein composition in supernatants and pellets of heterochromatin fraction after phase separation and centrifugation (Fig. 1E). To ensure reproducibility, proteins detected in only one replicate were removed from the analysis. Around 40% of heterochromatin proteins were exclusively detected in supernatants (HC_S_only), further confirming the boundary effect of heterochromatin condensates (Fig. 1E). Importantly, as phase separation occurs when multivalent weak interactions surpass the phase separation threshold required to maintain saturated protein concentration, phase separation proteins, in principle, are expected to partition in both pellet and supernatant fractions, such as linker histone H1 isoforms, MeCP2, lamin A/C, MBD2 and cbx5 (HP1a) (Fig. 1E). Western blot analysis further confirmed the presence of MBD2a, lamin A/C and H1 isoforms in the pelleted condensates (Fig. 1F and Supplementary Fig. S2B). Functionally, GO analysis revealed that proteins recruited into phase-separated condensates (HC_P) were tightly correlated with heterochromatin and subnuclear membraneless organelles, while proteins excluded from the phase-separated condensates (HC_S only) were strongly associated with histone acetylation (Fig. 1G). As protein phase separation is driven/influenced by intrinsic disorder and concentration, we compared protein abundance and disorder content across fractions (Supplementary Fig. S3D). Proteins in condensates only (HC_P only) and supernatant only (HC_S only) showed lower abundance than those in both condensates and supernatants (HC_P&S) (Supplementary Fig. S3E). However, HC_S only proteins exhibited markedly reduced disorder content relative to both HC_P&S and HC_P only (Supplementary Fig. S3F). These findings indicate that intrinsic disorder, in addition to abundance, is a key determinant distinguishing protein partitioning upon HC phase separation. In summary, all proteins detected in heterochromatin phase separated condensates (HC_P) were considered heterochromatin phase separation-associated proteins (Fig. 1E).

The data were then benchmarked against experimentally reported intrinsically disordered proteins in *Mus musculus* and *Homo sapiens* from DisProt (https://www.disprot.org/) (Fig. 1E, dark blue dots, and Supplementary Table S9). Among the 198 unstructured proteins detected in heterochromatin, 135 (∼68.2%) were found in the heterochromatin pellet, confirming that the heterochromatin isolation coupled with phase separation and MS is a reliable approach to identify candidate phase separation proteins in heterochromatin. Notably, protein concentration is a key determinant of phase separation, although thresholds vary among proteins. Indeed, the published heterochromatin phase separation proteins that were absent from the heterochromatin pellets exhibited relatively lower abundance (Fig. 1E).

In summary, we could show that the heterochromatin fraction underwent phase separation, enriching phase separation related proteins (scaffolds, regulators, and clients as mentioned in the introduction) into condensates while excluding others. However, potential contaminants from nuclear pore and nucleolus-related proteins due to spatial proximity and ensuing co-fractionation, as well as incomplete separation of pellets from supernatants, can not be overlooked. Therefore, additional strategies are necessary to identify the most likely phase separation scaffold proteins in heterochromatin.

### Predicting phase separation scaffold proteins using multiple machine learning-based predictors

**Figure 2.**
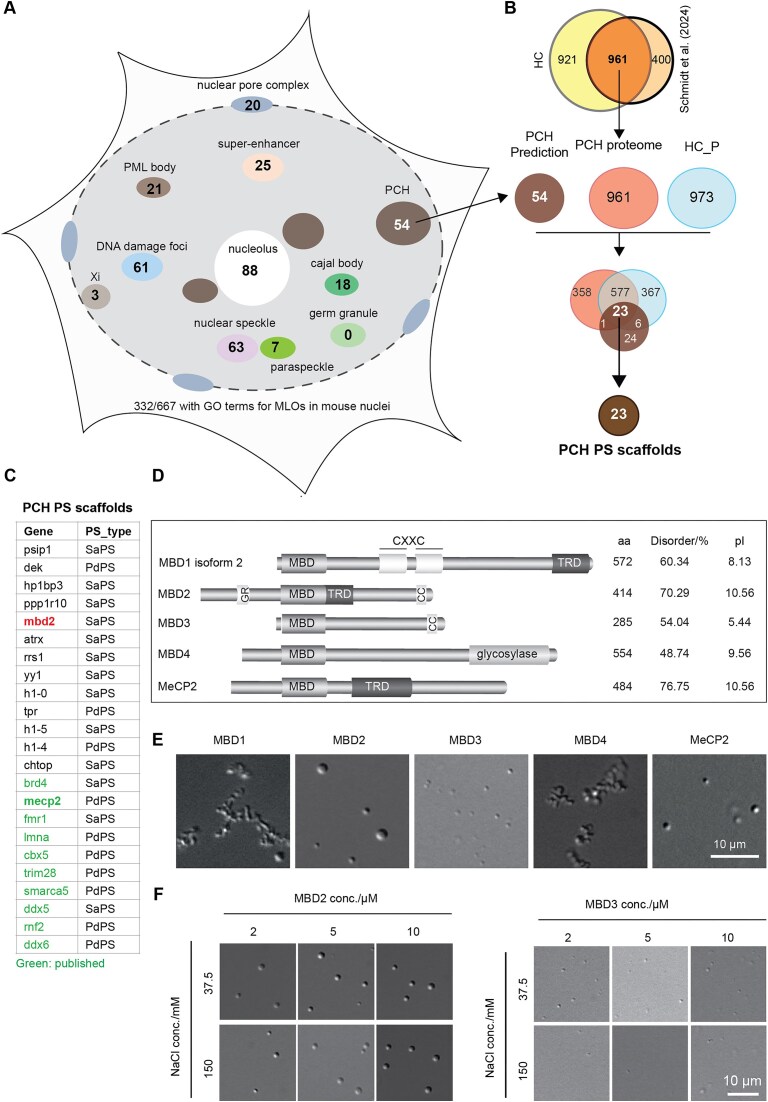
MBD2 is a candidate phase separation scaffold protein in mouse pericentric heterochromatin. **(A)** Number of predicted phase separation scaffold proteins with GO terms for multiple subnuclear membraneless organelles. **(B)** Venn diagram showing the overlap of three independent proteome-wide data to identify the most likely phase separation (PS) scaffold proteins in pericentric heterochromatin (PCH). “PCH Prediction” represented all predicted phase separation scaffold proteins with GO terms for pericentric heterochromatin compartment, as shown in panel (A). “HC_P” represents all proteins identified in the pellet fraction after heterochromatin phase separation and DIA_MS as shown in Fig. 1F. “PCH proteome” represents the pericentric heterochromatin proteomic data from Schmidt *et al.* [4]. **(C)** Table list of published (Green) and newly identified (black) phase separation scaffold proteins (PS scaffolds) in pericentric heterochromatin as shown in panel (B). PdPS, partner-dependent phase separation; SaPS, self-assembling phase separation. **(D)** Scheme summarizing the structures, length (aa), disorder scores, and isoelectric points (pI) of MBD-containing proteins. MBD: methyl-CpG binding domain; TRD: transcriptional repression domain. G/R: glycine/arginine; CC: coiled coil domain. The disorder scores were predicted using IUPred2A (https://iupred2a.elte.hu/). The isoelectric points (pI) were calculated using the peptide property calculator (https://pepcalc.com/). (**E, F**) Representative DIC images showing the phase separation properties of MBD-containing proteins. Following *in vitro* phase separation (Supplementary Fig. S6C), the mixtures were transferred to chambers made of double-sided tapes and sealed with coverslips 45 min after incubation at room temperature. The droplets were observed using a Nikon Eclipse TiE2 microscope equipped with DIC microscopy. Images were taken using a Nikon Eclipse TiE2 microscope equipped with DIC. *n* = three replicates. Scale bar = 10 μm.

Next, we aimed to identify candidate phase separation scaffold proteins (proteins capable of phase separation by themselves) utilizing multiple phase separation prediction tools proteome-wide. We first collected published phase separation scaffold proteins from various databases, including PhaSepDB [25], LLPSDB [26], and PhaSePro [27] (Supplementary Fig. S4A, left, and Supplementary Table S9). In mice, 324 intrinsically disordered proteins have been reported to undergo phase separation, characterized by distinct compositional features and biophysical/chemical properties, such as enrichment in glycine, arginine, proline, serine, asparagine, and aspartate, compared to the structured amino acid sequences [42]. Leveraging these features, several machine learning-based prediction tools (PS predictors) have been developed to predict the candidate phase separation scaffold proteins on a proteome-wide scale. Next, we applied three top-performing phase separation predictors: DrLLPS [18], PSAP [17], and PhaSePred [16]. Proteins predicted by at least two of the three predictors were considered candidate phase separation scaffold proteins, resulting in 854 predictions (Supplementary Fig. S4A, right, and Supplementary Table S9). Combining these with the published phase separation scaffold proteins, 1046 potential phase separation scaffold proteins were identified in the mouse proteome (Supplementary Fig. S4B), of which 667 are located in the cell nucleus based on their annotated subcellular localization collected from UniProt [28] (Supplementary Table S9). These proteins were further classified based on GO terms associated with various membraneless subnuclear organelles (Fig. 2A). In total 332 of the 667 nuclear protein candidates (∼50%) were categorized into different subnuclear compartments, while the remaining half were either uniformly distributed across the nucleus or had unknown subnuclear localization. In pericentric heterochromatin, 54 candidate phase separation scaffold proteins were identified (Fig. 2A and Supplementary Fig. S4B), among which 26 proteins, including cbx5 (HP1a) and MeCP2 [11, 43], have been experimentally validated for their phase separation properties, while 28 await experimental validation (Supplementary Table S9). Additionally, the phase separation scaffold proteins were further subdivided into self-assembling phase separation (SaPS) and partner-dependent phase separation (PdPS) proteins based on PhaSePred predicting scores [16] (Supplementary Table S9). However, the distinction between PdPS and SaPS remains somewhat ambiguous due to limitations in the training datasets. For instance, MeCP2 was originally classified as PdPS but was later demonstrated to phase separate by itself (SaPS) [11–13].

### MBD2 is a candidate scaffold for pericentric heterochromatin compartmentalization

We then sought to identify the high-confidence phase separation scaffold proteins in pericentric heterochromatin. To this end, we firstly compared the HC proteome dataset with the pericentric heterochromatin proteomic data reported by Schmidt *et al.* (2024) [4] (Fig. 2B and Supplementary Fig. S5A). 961 proteins were present in both datasets (PCH proteome; Supplementary Table S8), many of which are functionally associated with chromatin repression and membraneless subnuclear organelles (Supplementary Fig. S5B). In contrast, proteins uniquely identified in either datasets were enriched for functions related to active histone acetylation or mitochondrial activities, and were therefore excluded for the screening of phase separation scaffold proteins in pericentric heterochromatin. Then, we overlapped the PCH proteome with the HC_P proteomic data (HC_P) and with the predicted pericentric heterochromatin phase separation scaffold proteins (PCH prediction) (Fig. 2B and Supplementary Fig. S4C). This analysis identified 23 proteins present in all three datasets, each containing at least one long disordered region (Fig. 2C and Supplementary Fig. S4C–E). These proteins were therefore considered candidate pericentric heterochromatin phase separation scaffold proteins.

PCH is characterized by high levels of H3K9me3 and DNA cytosine methylation, which could be “read” by cbx5 (HP1a) and methyl-cytosine binding proteins, respectively. In addition to cbx5, MBD containing proteins such as MBD2 and MeCP2 were also identified with predicted scaffold phase-separation potential. By contrast, MBD1 and MBD3, two additional MBD-containing proteins detected in HC phase-separated condensates (Fig. 1E), showed no predicted scaffold phase separation properties. To experimentally validate/evaluate these predictions, we compared the phase separation abilities of five major MBD-containing proteins (MBD1-4, MeCP2) using *in vitro* phase separation assay (Fig. 2D and Supplementary Fig. S6A–C). Consistent with its prediction, MBD2 and MeCP2 formed spherical condensates with fusion properties, whereas MBD1 and MBD4 formed only irregular aggregates (Fig. 2E) (Movie 1). MBD3, which shares ∼80% homology with MBD2 outside the MBD domain [44] exhibited a much weaker phase separation capacity, forming only small condensates in all conditions (Fig. 2F). Thus, the strategy combining experimental recognition (heterochromatin isolation, phase separation and condensate composition detection) and *in silico* prediction is capable of isolating/recognizing candidate scaffold phase separation proteins in pericentric heterochromatin.

Taken together, these findings suggested that MBD2 may regulate pericentric heterochromatin dynamics via phase separation. Hence, we next explored the molecular determinants of MBD2 phase separation and its role in heterochromatin organization.

### MBD2 spherical condensate formation is driven by its C-terminus and modulated by the CC domain in an isoform dependent manner *in vitro*

**Figure 3.**
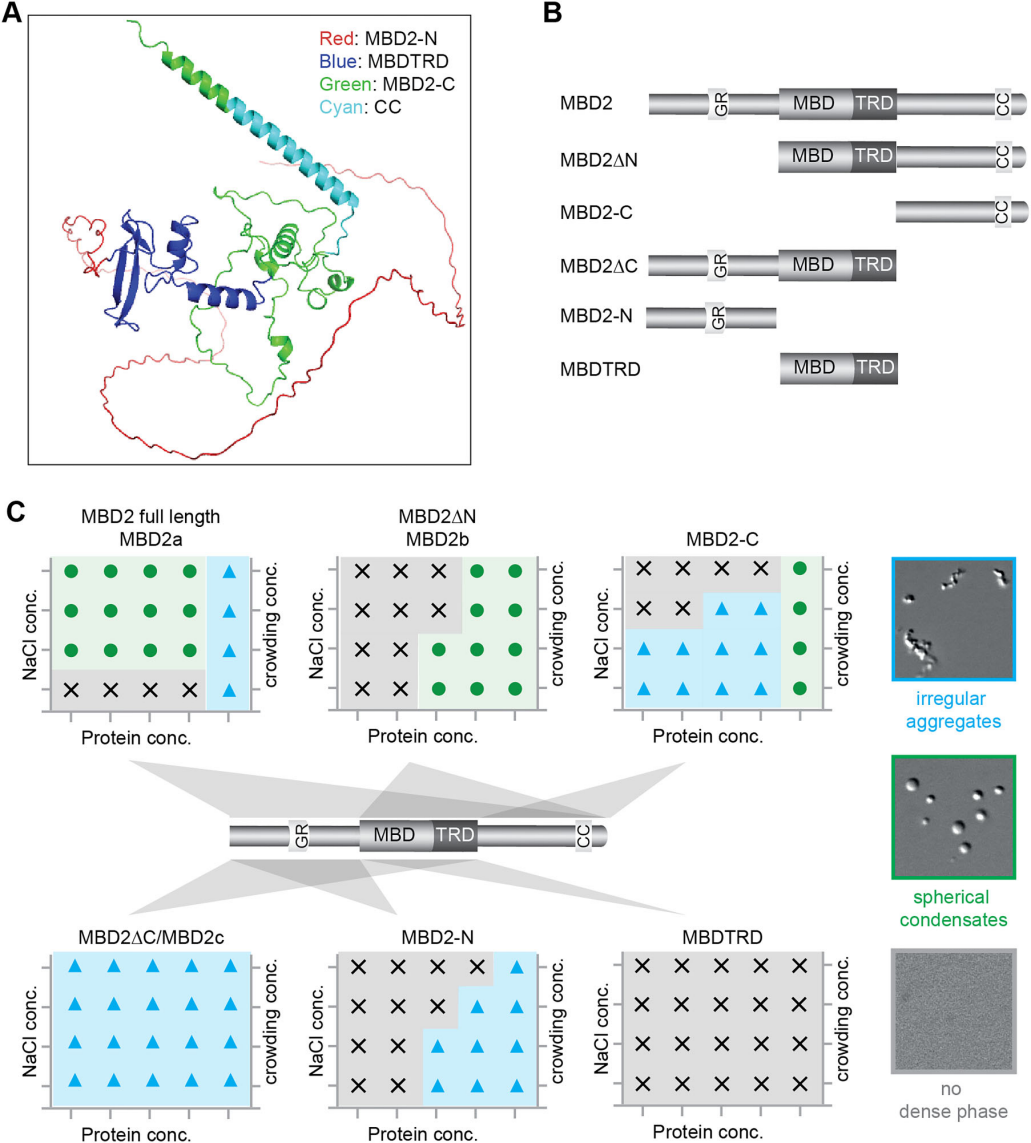
MBD2 forms spherical condensates that depend on the C-terminus. **(A)** MBD2 structure prediction using the AlphaFold Server (https://alphafoldserver.com/). The lines indicated the unstructured regions. Known MBD2 protein domains were color-coded as indicated. **(B)** Scheme showing the MBD2 constructs applied for *in vitro* phase separation assay. **(C)** Summarized phase diagrams of MBD2 constructs with representative microscopic images. MBD2 forms two kinds of distinct condensates with different morphology: (i) liquid-like spherical droplets with smooth and well-defined boundaries; (ii) solid/gel-like irregular aggregates with rough, uneven, or jagged boundaries. *n* = three replicates. Full information and images are shown in Supplementary Fig. S7.

MBD2 contains three regions, the well-defined MBDTRD, the amino terminus (N) before the MBDTRD, and the carboxyl terminus (C) after the MBDTRD (Supplementary Fig. S1). Structure prediction using the AlphaFold server showed that MBD2 is highly disordered outside the functional MBDTRD domain (Fig. 3A) [29]. To explore how different regions contribute to the phase separation properties of MBD2, we performed *in vitro* phase separation assays with purified MBD2 truncations from either the amino terminus or the carboxyl terminus (Fig. 3B and Supplementary Figs S6D, S7, and S8).

The full-length MBD2 formed spherical condensates at high protein and low salt concentrations and irregular aggregates in the presence of crowding agents (PEG 8000) (Fig. 3C and Supplementary Fig. S7). In comparison, truncating from the amino terminus [MBD2∆N, and MBD2∆N∆MBDTRD (MBD2-C)] reduced the likelihood of forming spherical condensates. Both irregular and spherical condensates were detected under different conditions. However, deletion of the carboxyl terminus (MBD2∆C, MBD2-N, and MBDTRD) abolished the ability to form spherical condensates. Thus, the carboxyl terminus is essential for spherical condensate formation, which could be enhanced by the amino terminus. Considering its DNA binding ability, we further checked the contribution of DNA in MBD2 spherical condensate formation (Supplementary Fig. S8). DNA promoted the transition of MBD2 condensates from spherical ones to irregular aggregates, inhibited MBD2∆N condensate formation, and enhanced MBD2∆C irregular aggregates formation.

**Figure 4.**
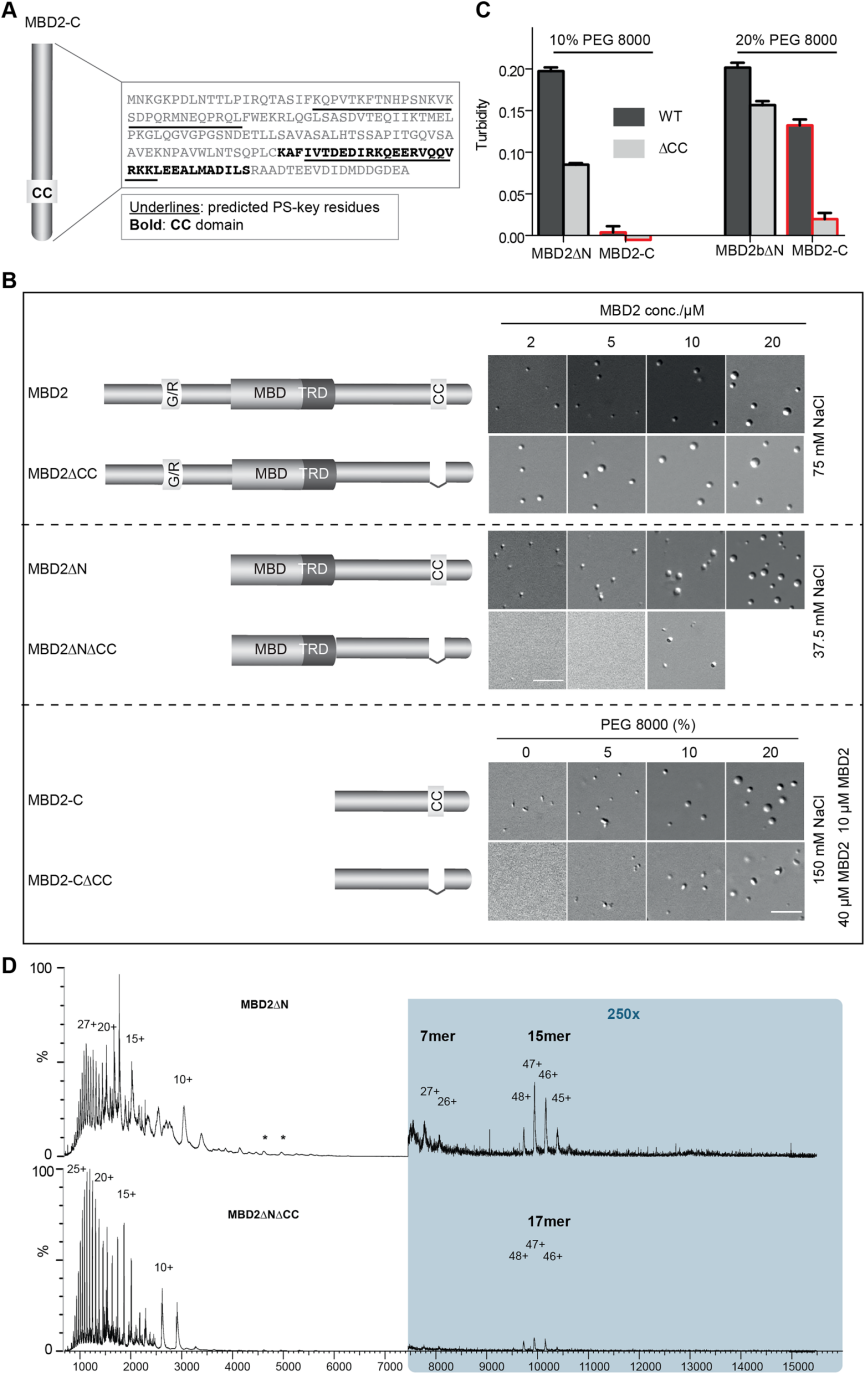
CC domain modulates MBD2 spherical condensate formation in a domain-dependent manner *in vitro*. **(A)** Prediction of the key phase separation residues within the MBD2 carboxyl terminus assessed using PSPHunter (http://psphunter.stemcellding.org/). The CC domain is highlighted in cyan. The predicted key residues are underlined. **(B)** Graphic summary of the phase separation properties of MBD2 constructs with and without CC domain. *n* = three replicates. **(C)** Quantification of turbidity assay upon phase separation of MBD2 constructs with or without CC domain at different conditions. Absorbance at 340 nm was plotted as mean ± SD. *n* = three replicates. Raw data can be found in Supplementary Table S10. **(D)** Spectra of MBD2∆N (top), MBD2∆N∆CC (bottom) show monomer in multiple charge states in the low m/z range. The observed charge state envelopes of both monomers are characteristic for partially disordered proteins. Both variants of MBD2∆N form oligomers of 467 kDa at m/z 10 000 (A: MBD2∆N 15mer; B: MBD2∆N∆CC 17mer). Intermediate oligomers are observed for MBD2∆N at m/z 8000 (A: 7mer, 210 kDa). The * annotation marks peaks corresponding to the identified contaminant DnaK (*E. coli*, 69 kDa).

Further analysis by the PSPHunter predicted two phase separation key regions in the carboxyl terminus, one of which is located within the structure CC domain (Fig. 4A) [30]. Moreover, the CC is predicted to undergo dimerization by the AlphaFold server (Supplementary Fig. S9A), suggesting that the CC-mediated self-interaction drives the MBD2 spherical condensate formation. Indeed, deleting the CC domain in MBD2∆N and MBD2-C abolished the ability to form spherical condensates, although the full-length MBD2 with CC deletion showed even stronger spherical condensate formation ability (Fig. 4B and Supplementary Fig. S10). This suggested that the other regions of MBD2 carboxyl terminus also exhibited phase separation properties, which were largely repressed by CC, such as the other predicted phase separation key region shown in Fig. 4A. Alternatively, previous reports regarding the function of MBD2 residues R286L287 in direct interaction with the histone deacetylase core of NuRD suggest possible functions of R286L287 in such weak interactions [45, 46]. Further, the necessity of CC in MBD2 phase separation was confirmed by turbidity assay and condensate sedimentation assay with lower turbidity in the absence of CC (Fig. 4C) and less protein fraction in condensates (Supplementary Fig. S9B and C). Moreover, using native mass spectrometry, we detected the presence of oligomers (from pentamers up to 26mer forms) in the MBD2∆N condensate fraction, which was largely abolished by the depletion of CC (Fig. 4D). Altogether, this implies that MBD2 phase separation is likely initiated by CC domain-based dimerization and further stabilized by multiple weak interactions mediated by the disordered C terminus outside the CC domain.

Additionally, the amino terminus containing truncations (MBD2∆C and MBD2-N) showed a strong ability to form irregular aggregates, consistent with phase transition behavior. The PSPHunter predicted two phase separation key regions within the amino terminus, which are separated by the low complexity glycine-arginine (GR) repeat region (Supplementary Fig. S11A). The GR-mediated separation of the two phase separation key regions potentially increased the intramolecular interactions between the two regions. As the intramolecular interactions could influence the phase separation properties, we hypothesized that the GR regulates the phase separation behavior of MBD2. Supporting this hypothesis, we found that the amino terminus formed only spherical droplets in the absence of GR (MBD2-N∆GR), and this was enhanced by the carboxyl terminus (Supplementary Fig. S11B).

In summary, we found that the C-terminus drives the MBD2 phase separation and spherical condensate formation, which could be modulated by CC domain in a domain dependent manner *in vitro*, whereas the N-terminus drives the phase transition and irregular aggregate formation in a GR dependent manner by enhancing the intramolecular interactions of the amino terminus.

### MBD2 modulates pericentric heterochromatin dynamics in an isoform-dependent manner

**Figure 5.**
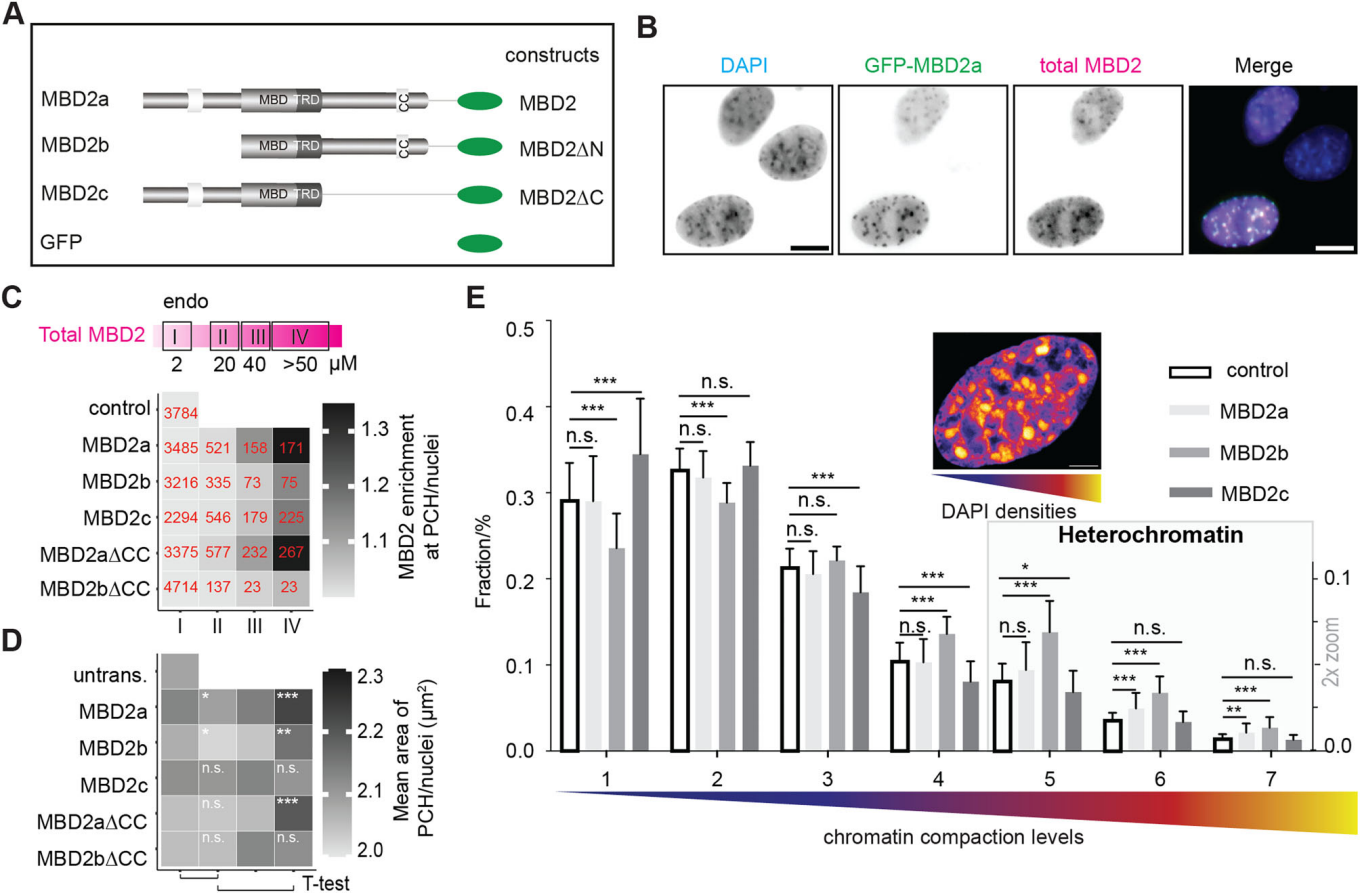
MBD2a/b influences chromatin compaction. **(A)** Schematic representation of MBD2 isoforms and constructs used for ectopic expression. **(B)** Representative images of ectopic MBD2a and total MBD2 distribution within nuclei. Total MBD2 levels were detected by immunofluorescence staining using antibody against the MBD domain of MBD2 and Cy5-conjugated secondary antibody, followed by fluorescence imaging using a Nikon Eclipse TiE2 microscope equipped with a Plan Apo λ 40× air objective. (**C, D**) Heat map showing the influence of MBD2 isoforms on their subnuclear localization **(C)** and pericentric heterochromatin compartment sizes **(D)** with and without CC domain. Nuclei were subclassified into four categories based on their total MBD2 levels (I: endogenous, 2–3 µM as calculated in Supplementary Fig. S12D). Assuming a linear relationship of Cy5 fluorescence and total MBD2 levels, we estimated the protein levels (µM) of each nucleus and subgrouped them based on their MBD2 levels: II 20 µM (10–30 µM); III 40 µM (30–50 µM); and IV > 50 µM. For the heat map, mean values were plotted. Cell numbers in each condition were shown in panel (C). Significances were determined by pairwise *t*-test with Benjamini–Hochberg (BH) correction false discovery rate (FDR) correction. ns, no significance, *P* >.05; *P ≤.05; **P ≤.01; ***P ≤.001. Raw data can be found in Supplementary Table S10. **(E)** Bar plot showing the effect of the different MBD2 isoforms on the compaction of chromatin. The cell nucleus was classified into seven different chromatin compaction classes based on the DAPI intensities from DNA-free interchromatin region (class 1) to highly active and less compacted euchromatin (classes 2–4) and to highly compacted heterochromatin (classes 5–7). 3D images were taken using Leica TCS SP5 II confocal microscope with a HCX PL APO 100×/1.44 oil Corr CS objective. 3D images with similar MBD2 protein levels were taken and used for quantitative analysis. Data are represented as mean ± SD. *n* (control) = 33. *n* (MBD2a) = 32. *n* (MBD2b) = 34. *n* (MBD2c) = 32. Significances were calculated by an unpaired *t*-test. ns, no significance, *P* >.05; *P ≤.05; **P ≤.01; ***P ≤.001. Raw data can be found in Supplementary Table S10.

MBD2 exists in three isoforms due to alternative splicing and translational start sites: MBD2a (full length MBD2), MBD2b (corresponding to MBD2∆N), and MBD2c (corresponding to MBD2∆C) (Fig. 5A). MBD2a and MBD2b exhibit similar expression profiles across differentiation, whereas MBD2c is regulated differently [47]. *In vitro*, both MBD2a and MBD2b formed liquid-like spherical condensates, whereas MBD2c failed to do so (Fig. 3). These indicate that phase separation differences of MBD2a/b and MBD2c possibly reflect their functional differences in heterochromatin compartmentalization *in vivo*.

Hence, we checked how MBD2 isoforms influence the pericentric heterochromatin architecture (Fig. 5B–E, and Supplementary Fig. S12). Firstly, we reanalyzed and compared the levels of MBD2 across mouse tissues using the proteomics data from Geiger *et al.* (2013) (Supplementary Fig. S12A) (data downloaded form PaxDB database; Supplementary Table S7) [48]. MBD2 levels varied widely, with the lowest abundance in muscle. Consistently, our previous work showed low MBD2 levels in C2C12 myoblast cells, a mouse skeletal muscle cell line [49]. Moreover, MBD2 was shown to promote C2C12 differentiation into myotubes via interaction with focal adhesion kinase together with increased MBD2 expression [50]. Thus, C2C12 cells were chosen as the *in vivo* model to examine how MBD2 contributes to the heterochromatin organization. The endogenous MBD2 concentration in C2C12 myoblasts is ∼1.9 µM for MBD2a and 0.9 µM for MBD2b (Supplementary Fig. S12B–D). Combined with the proteomics data from Geiger *et al.* (2013) [48], we further estimated MBD2 abundance across mouse tissues to range from ∼3 µM in muscle to ∼320 µM in ileum, with a median of ∼41 µM and a mean of ∼79 µM (Supplementary Fig. S12A, bottom).

In C2C12 cells, endogenous MBD2 was enriched in DAPI-densed pericentric heterochromatin regions with modest heterogeneity in abundance and slightly restricted pericentric heterochromatin compartment size (Supplementary Fig. S12E–H). This was further confirmed in ES cell (Supplementary Fig. S13), a cell line with similar MBD2 abundance to C2C12 cells (Supplementary Fig. S12D). To directly test the functional contribution of endogenous MBD2, we generated an MBD2 knockout ES cell line (Supplementary Fig. S13A–E). Consistently, endogenous MBD2 in ES cells modestly constrained pericentric heterochromatin compartment size (Supplementary Fig. S13F and G) and its deletion resulted in enlarged compartment size (Supplementary Fig. S13H).

We next checked how ectopic expression of MBD2 isoforms influences the pericentric heterochromatin dynamics in C2C12 cells after transfection and immunofluorescence (Fig. 5 and Supplementary Fig. S14). All MBDTRD-containing constructs showed enrichment at pericentric heterochromatin via specific MBD–mC interactions [51], while the carboxyl terminus (MBD2-C) exhibited widespread distribution across the whole cell except for the nucleolus (Supplementary Fig. S14A). Cells were stratified by total MBD2 concentration into four groups (I: ∼2 µM [endogenous]; II: ∼20 µM; III: ∼40 µM; IV: >40 µM). All MBD2 isoforms enriched at PCH in a dose-dependent manner, though to different extents (Fig. 5C). MBD2c showed no significant effect on pericentric heterochromatin compartment size. In contrast, MBD2a and MBD2b reduced the size of pericentric heterochromatin compartments at lower levels (< 20 µM) but significantly enlarged the pericentric heterochromatin compartment size at higher levels, independent of the CC domain (Fig. 5D). Moreover, we noticed that the MBDTRD domain, the core region of MBD2 with stable 3D structures to bind methyl-cytosine (mC) and recruit cofactors, promoted the growth of pericentric heterochromatin compartments in a dose dependent manner (Supplementary Fig. S14B). These data indicate that MBD2 modulates pericentric heterochromatin compartment size via mechanisms independent of phase separation.

In theory, phase separated liquid-like condensates exhibit boundary effects, restrict molecule exchange and modulate condensate compositions, e.g. nucleic acids, proteins. Next, we measured the influence of MBD2 on DNA distribution (Fig. 5E and Supplementary Fig. S14C and D). The nuclei were classified into seven chromatin compaction classes based on the DAPI intensity (Fig. 5E). Compared to the control cells (GFP only), MBD2a increased the fractions of highly compacted heterochromatin (classes 5–7) and concomitantly decreased the fractions of low chromatin compaction (classes 2–3). This effect was further enhanced by CC deletion (Supplementary Fig. S14C), which strengthens spherical condensate formation (Fig. 3B). MBD2b strongly increased the fractions of highly compacted heterochromatin (Fig. 5E), which however was abolished by CC deletion (Supplementary Fig. S14D), a construct with weaker spherical condensate formation ability (Fig. 3B). MBD2c, which did not form spherical condensate, showed no influence on DNA distribution (Fig. 5E).

Taken together, this indicates a positive correlation between the ability of MBD2 to form spherical condensates and its capacity to modulate heterochromatin compaction. MBDTRD modulates pericentric heterochromatin compartment size, whereas the CC fine-tunes condensate morphology and chromatin compaction and reorganization.

### MBD2a/b spherical condensates generate an interfacial barrier surrounding pericentric heterochromatin compartments

In addition to self-assembling phase separation forming liquid-like spherical condensates [SaPS, or liquid–liquid phase separation (LLPS)], recent work has suggested an alternative phase separation mechanism known as partner-dependent phase separation (PdPS) [16]. Unlike SaPS, PdPS proteins cannot establish sufficient weak homo interactions to overcome the phase separation threshold. Instead, multivalent weak interactions with large polymers drive the protein phase separation. Thus the process is also called polymer–polymer phase separation (PPPS) [52] . Condensates formed by LLPS (SaPS) and PPPS (PdPS) exhibit distinct properties. One hallmark distinguishing them is that the LLPS-mediated condensates have an interphase barrier which creates a protein pool with high concentrations of free protein molecules and generates apparent interfacial barriers. These interfacial barriers result in preferentially intra-condensate molecular exchanges and restrict the inter-phase molecular exchange, while PPPS-mediated aggregates do not [53].

**Figure 6.**
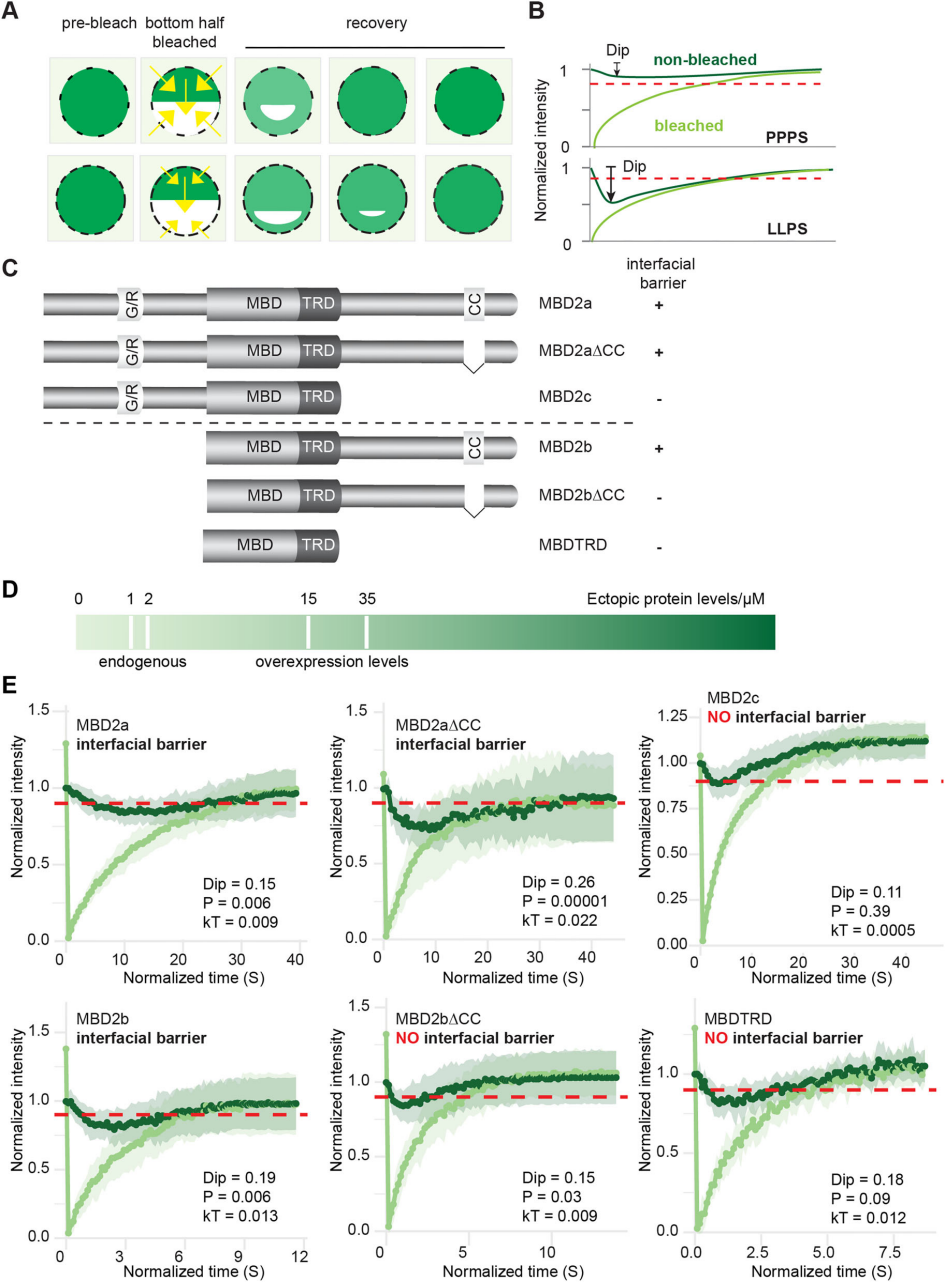
MBD2a/b generates interfacial barriers surrounding pericentric heterochromatin compartments. **(A)** Schematic representation of the half-FRAP strategy. Yellow arrows indicate whether the fluorescence protein predominantly diffuses from within the heterochromatin compartment or the surrounding nucleoplasm and their sizes indicate how much/fast from which compartment. **(B)** Corresponding fluorescence recovery in the bleached and unbleached half of a condensate without (top) and with (bottom) interfacial barrier. The maximum intensity decrease of the nonbleached half is referred to as “Dip”. The red dashed line indicates the threshold. **(C)** Schematic representation of MBD2 constructs (MBD2s). **(D)** Quantification and selection of cells with ectopic MBD2 levels from 15–35 µM/nucleus. A detailed quantification method is shown in Supplementary Fig. S16. **(E)** Normalized fluorescence changes in the bleached half (light green) and the nonbleached half (dark green) for different MBD2 constructs. P (Student test against dip in free solution) < 0.01 was considered as an apparent interfacial barrier. kT: Energy barrier per molecule. *n* (MBD2a) = 23. *n* (MBD2a∆CC) = 17. *n* (MBD2c) = 19. *n* (MBD2b) = 20. *n* (MBD2b∆CC) = 17. *n* (MBDTRD) = 7. For additional details, see Methods. Raw data can be found in Supplementary Table S10.

Given that the MBD domain binds specifically to methylated DNA and MBDTRD itself could not phase separate (Fig. 3B and Supplementary Fig. S7), we examined if DNA or methyl-DNA (mC-DNA) could promote MBDTRD phase separation. Indeed, MBDTRD formed irregular condensates in the presence of methylated DNA (Supplementary Fig. S15A). Thus, both CC-mediated homo-interaction and MBD–mC-mediated hetero-interaction probably contribute to MBD2 phase separation and pericentric heterochromatin reorganization. To dissect the underlying mechanism of MBD2 phase separation in the pericentric heterochromatin region, we made use of partial compartment fluorescence recovery after photobleaching (half-FRAP) approach recently coined MOCHA-FRAP [40]. In brief, we bleached one half of a pericentric heterochromatin compartment and measured the fluorescence intensity in the bleached and the nonbleached half in C2C12 cells (Fig. 6A). The fluorescence of the beached half recovered due to molecule exchange with the unbleached half and the surrounding region. In the absence of an interfacial barrier or with a weak interfacial barrier, molecular exchanges between the surrounding and bleached half as well as between the nonbleached and bleached half occur with similar kinetics, resulting in only a subtle and transient fluorescence decrease in the nonbleached half (smaller “Dip” values). Conversely, in the presence of a strong interfacial barrier, there is a preferential intra-pericentric heterochromatin mixture between the bleached and nonbleached half, leading to a significant initial fluorescence decrease in the nonbleached half (bigger “Dip” values). At later time points, the fluorescence recovery shows the same kinetics as the bleached half (Fig. 6B). Cells with ectopic MBD2 abundance from 15 to 35 µM (levels found in tissues) were chosen (Fig. 6C and D and Supplementary Fig. S16). We found that the CC domain-containing constructs exhibited significant interfacial barriers, while all CC-depleted and carboxyl terminus-depleted constructs exhibited no apparent interfacial barriers except for MBD2a∆CC, which showed stronger spherical condensate formation ability *in vitro* (Fig. 6C and E).

In order to verify the MOCHA-FRAP data, we performed fluorescence loss in photobleaching experiments with different MBD2 constructs, analyzed the fluorescence loss in unbleached nucleoplasm (blue rectangles) and heterochromatin compartments (green rectangles) after bleaching of the nucleoplasm (red dashed circles) (Supplementary Fig. S17A). The unbleached nucleoplasm regions rapidly lost ∼20% fluorescence upon photobleaching for all MBD2 constructs (Supplementary Fig. S17B). In the heterochromatin compartments, the MBD2a, MBD2a∆CC, and MBD2b showed no fluorescence changes, while MBD2c, MBD2b∆CC, and MBDTRD showed a fast fluorescence loss upon photobleaching of nucleoplasm within the same nuclei (Supplementary Fig. S17B). These indicate that the MBD2a, MBD2a∆CC, and MBD2b form apparent interfacial barriers between the heterochromatin compartments and the nucleoplasm.

Therefore, MBD2 first locally enriches (seeds) at the pericentric heterochromatin region via the mC–MBD interactions thereby overcoming the phase separation threshold. Subsequently, MBD2a/b forms liquid-like condensates via C-terminus-mediated homo-oligomerization, generates apparent interfacial barriers, and ultimately modulates the pericentric heterochromatin composition.

### MBD2 liquid-like spherical condensates modulate the composition of heterochromatin

To explore whether and how MBD2a/b condensates modulate the heterochromatin composition, we firstly detected the localization of MBD2 in reconstituted heterochromatin condensates. The purified MBD2 molecules were labelled with Alexa Fluor 546 (AF546-MBD2) and mixed with unlabelled MBD2 proteins in a ratio of 1:99, followed by *in vitro* phase separation assay. All MBD2 condensates could be labelled with AF546, confirming that the fluorescence probe does not influence the MBD2 phase separation (Supplementary Fig. S15B, left). Then high MBD2 concentration (10 µM MBD2 with 0.1 µM AF546-MBD2 with MBD2 phase separation) and low MBD2 concentration (0.1 µM AF546-MBD2 without MBD2 phase separation) were mixed with heterochromatin (0.5 µg/µl heterochromatin fractions with heterochromatin phase separation) for *in vitro* phase separation, separately. AF546-MBD2 was homogeneously incorporated into all heterochromatin condensates regardless of whether having added MBD2 condensates or not (Supplementary Fig. S15B and C). Importantly, irregular heterochromatin aggregates transitioned into larger and more spherical condensates upon additional MBD2 condensates (Supplementary Fig. S15B).

**Figure 7.**
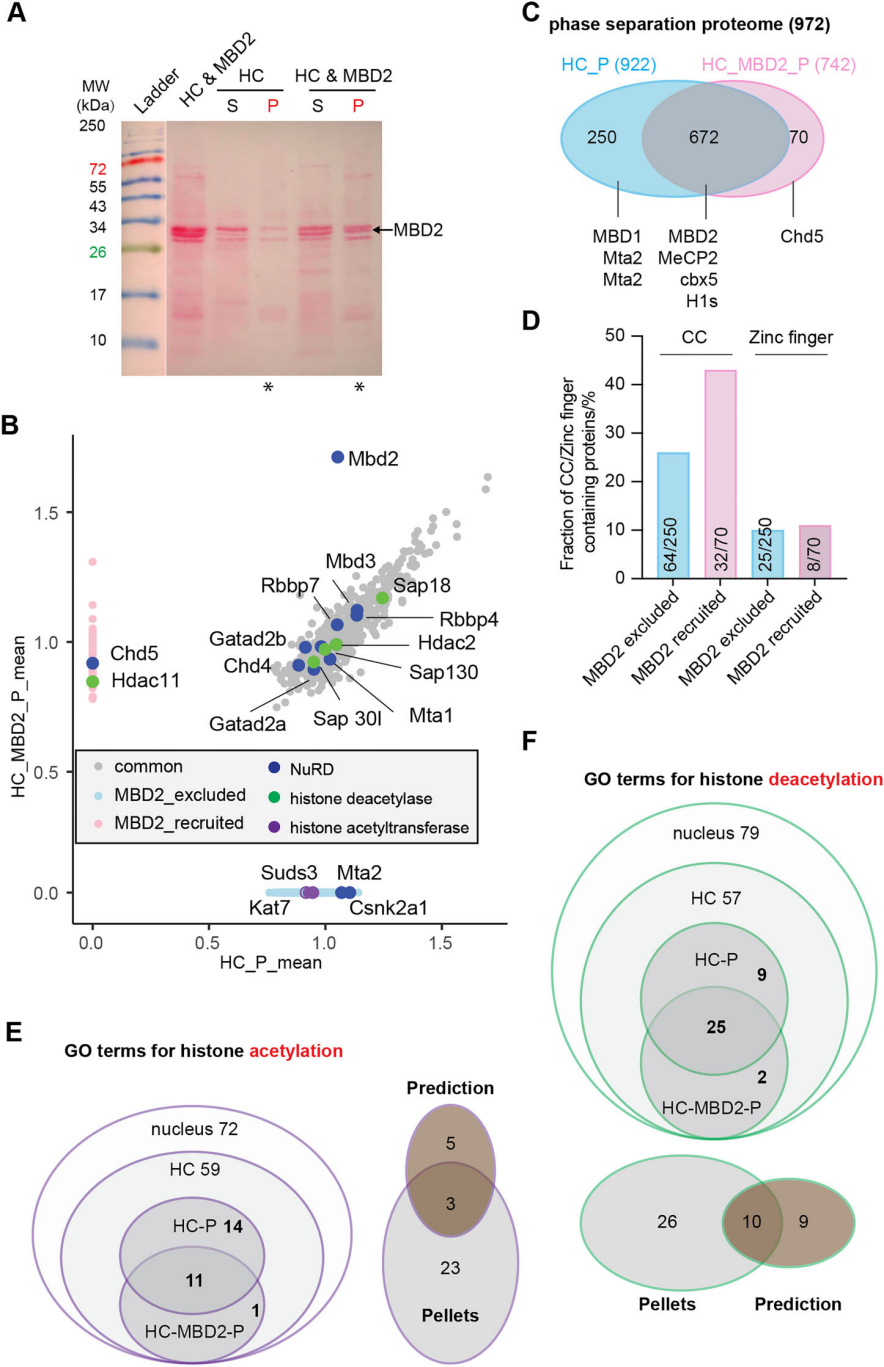
MBD2a/b spherical condensation-dependent interfacial barrier modulates the heterochromatin proteome. **(A)** Ponceau staining after SDS–PAGE showing the compositional differences among mouse brain heterochromatin fraction (HC), *in vitro* heterochromatin condensate assay supernatant **(S)** and pellet **(P)** in the presence or absence of additional MBD2 condensates. * indicate the fractions used for downstream mass spectrometry analysis in panel (B). **(B)** Dot plot of protein mean intensities in HC_MBD2_P versus those in HC_P. Only proteins detected in ≥2 replicates were considered in each group. Gray dots: all proteins found in heterochromatin pellets, regardless of additional MBD2 condensates. Pink dots: Proteins specifically recruited into the condensates by additional MBD2 condensates. Light blue dots: Proteins specifically excluded from the condensates by additional MBD2 condensates. Dark blue dots: NuRD members. Green dots: Histone deacetylase-related proteins. Purple dots: Histone acetyltransferase-related proteins and histone deacetylase repressors. *n* = three replicates. **(C)** Venn diagram showing the number of proteins identified in the heterochromatin pellets in the presence and absence of additional MBD2 condensates as shown in panel (B). Representative proteins in each sub-class are listed. **(D)** Fraction of coiled coil and zinc finger-containing proteins in heterochromatin fractions that were specifically recruited or excluded by MBD2 condensates. The coiled coil and zinc finger domains were predicted using InterPro (https://www.ebi.ac.uk/interpro/). **(E)** Left: Number of histone acetylation-related proteins identified in different fractions of mouse brain nuclei. Right: Venn diagram showing the overlap between histone acetylation-related proteins identified in pellets and those predicted with scaffold phase separation properties. **(F)** Top: Number of histone deacetylation-related proteins identified in different fractions of mouse brain nuclei. Bottom: Venn diagram showing the overlap between histone deacetylation-related proteins identified in pellets and those predicted with scaffold phase separation properties.

Molecularly, distinct proteins were enriched in the pellets compared to the supernatant in the presence of additional MBD2 condensates (HC_MBD2_S versus HC_MBD2_P) (Fig. 7A), in agreement with MBD2 being a scaffold factor driving large-scale heterochromatin phase separation. Furthermore, the protein composition of HC condensates differed in the presence of additional MBD2 condensates (HC_MBD2_P) compared to heterochromatin alone (HC_P) (Fig. 7A). Thus, we compared the protein compositional differences of heterochromatin condensates with and without additional MBD2 condensates using mass spectrometry (Fig. 7B and C, Supplementary Fig. S18A and B, and Supplementary Table S8). A total of 922 and 742 proteins were detected in heterochromatin condensates in the absence and presence of additional MBD2 condensates, respectively (Fig. 7C, left). Among these, 672 proteins were consistently detected including MeCP2 and cbx5, 250 were excluded from the heterochromatin condensates including MBD1, and 70 proteins were enriched into the condensates upon addition of MBD2 condensates. Both MBD2 condensate recruited and excluded proteins exhibited mildly lower disorder and abundance (Supplementary Fig. S18C and D). Moreover, we found that MBD2 condensate containing phase separation showed a preference for recruiting CC domain-containing proteins (but not zinc-finger domain-containing proteins), likely via hetero CC interactions (Fig. 7D). This is further evidenced by a recent report that CC pairing drives protein phase separation *in vivo* and *in vitro* [54]. These findings indicate that MBD2 modulates heterochromatin protein composition in part through phase separation.

### MBD2a/b modulates heterochromatin epigenetics via phase separation-driven molecular exclusion and inclusion

Given that a hallmark of heterochromatin is low histone acetylation levels, which could be brought about by MBD2-containing histone deacetylation complexes, we hypothesized that MBD2a/b-driven spherical condensate formation might influence the distribution of histone (de)acetylation-related proteins, thereby having functional consequences. Indeed, the proteins excluded by MBD2 condensates showed an enrichment for active histone acetyltransferase (Supplementary Fig. S18D). Further, we examined the distributions of histone acetylation and deacetylation-related proteins (Fig. 7E and F). We found that additional MBD2 condensates substantially excluded histone acetylation-related proteins from heterochromatin condensates to ∼56% (14/25) (Fig. 7E, left) (Supplementary Table S8). Interestingly, only a small portion of these proteins (3/26) were predicted to have phase separation properties (Fig. 7E, right). In contrast, histone deacetylation-related proteins were largely retained within heterochromatin condensates (∼74%, 25/34) regardless of additional MBD2 condensates, possibly due to the intrinsic phase separation properties of other deacetylation components with predicted scaffold phase separation properties (10/36) (Fig. 7F).

MBD2 and MBD3 are well-known scaffold proteins for the assembly and localization of the nucleosome remodelling and histone deacetylation (NuRD) complex [55]. Unlike MBD2, which is enriched in highly methylated inactive heterochromatin, MBD3 is more widely distributed across the nucleus due to a lack of mC binding [49, 55, 56]. Both MBD2 and MBD3 are expressed in the mouse brain and were detected in heterochromatin condensates (Fig. 7B) although MBD3 itself showed much weaker phase separation properties (Fig. 2F). We hypothesized that MBD2 promotes the local enrichment of NuRD members and subsequent NuRD assembly via spherical condensation.

Among the 41 reported NuRD-related proteins, 18 and 16 were detected in the mouse brain nuclei and heterochromatin fraction, respectively (Supplementary Fig. S19A). Additionally, 12 and 11 NuRD members were detected within heterochromatin condensates with and without additional MBD2 condensates (Supplementary Fig. S19A, left, and Supplementary Table S8) with a preference for CC domain-containing proteins (such as MBD2; Supplementary Fig. S19A, right). Notably, only MBD2 exhibited predicted phase separation scaffold properties (Supplementary Fig. S19A, bottom). Consistently, we found that ectopic expression of MBD2a/b promoted the relocalization and enrichment of the NuRD member GATAD2b into DNA-dense and MBD2-enriched pericentric heterochromatin regions in a dose-dependent manner in both C2C12 mouse myoblasts and ES cells (Supplementary Fig. S19B–E). This was not the case when overexpressing MBD2c (Supplementary Fig. S19B–E), the isoform with no spherical condensate formation ability (Fig. 3C). Consistently, MBD2 deletion resulted in decreased GATAD2b abundance and enrichment at pericentric heterochromatin compartments (Supplementary Fig. S19F and G). These data indicate that MBD2 facilitates the local enrichment and subsequent assembly of NuRD complexes within pericentric heterochromatin compartments, which is correlatively consistent with a possible contribution of MBD2 phase separation to NuRD assembly. However, our current data cannot directly distinguish the effects of direct MBD2–NuRD protein interactions from potential condensate-mediated recruitment, and more precise strategies are required in future studies.

**Figure 8.**
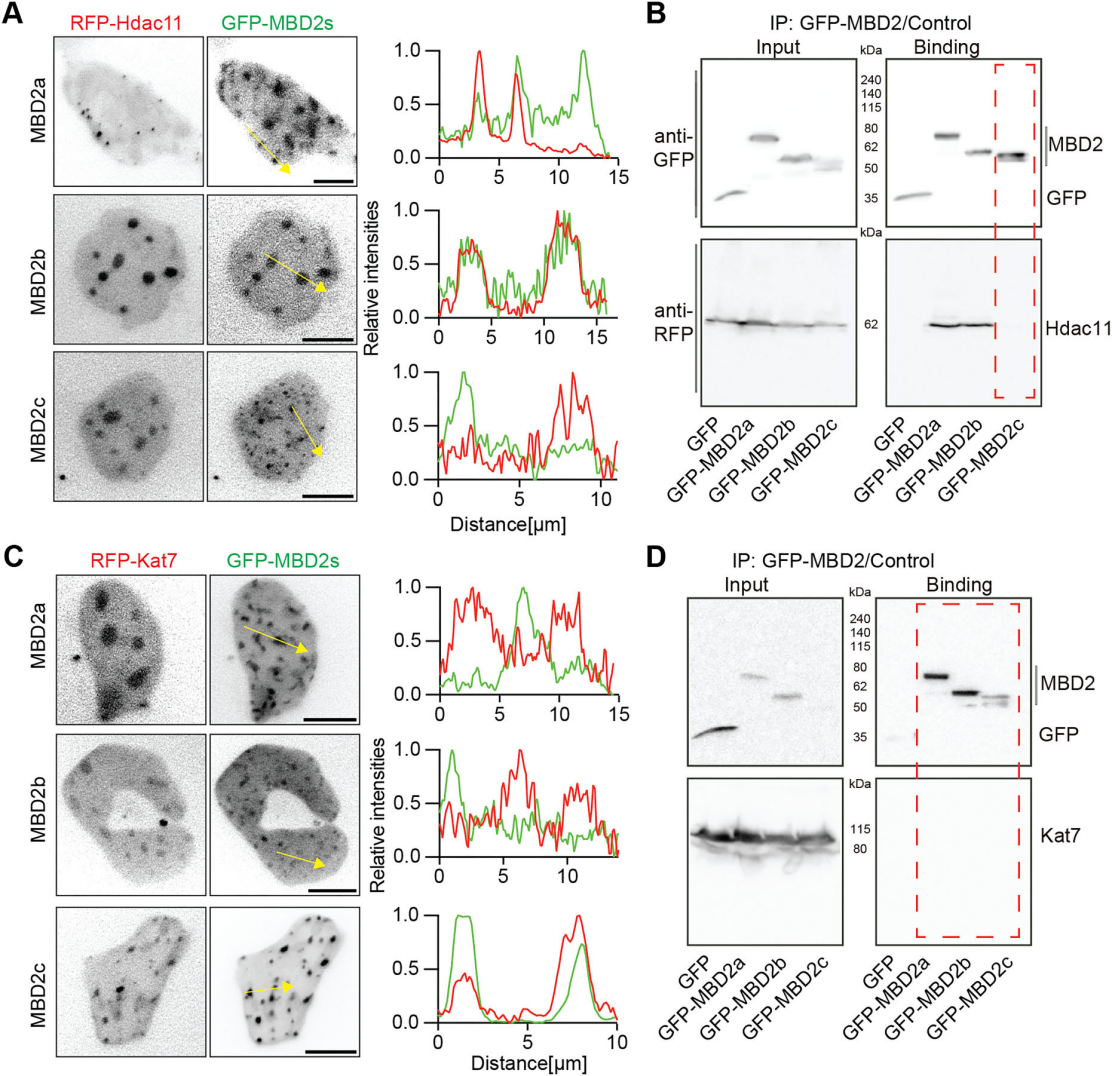
MBD2a/b Interacts with Hdac11 but excludes Kat7. (**A, B**) MBD2–Hdac11 interactions. **(A)** Representative images (left) showing the localization of GFP-tagged MBD2 isoforms and RFP-tagged Hdac11 in ES J1 cells 48 h after cotransfection. Line profiles (right) depict fluorescence intensity along the yellow lines. **(B)** Co-immunoprecipitation analysis of MBD2–Hdac11 interaction in HEK cells. GFP-tagged MBD2a, MBD2b, MBD2c, or GFP as a control were immunoprecipitated using GFP-binder beads. Pull-down fractions were analyzed by western blot using anti-RFP. Input and bound fractions are shown. Dashed red boxes highlight the absence of pull-down. (**C, D**) MBD2–Kat7 interactions. **(C)** Representative images (left) showing the localization of GFP-tagged MBD2 isoforms and RFP-tagged Kat7 in ES J1 cells 48 h after cotransfection. Line profiles (right) depict fluorescence intensity along the yellow lines. **(D)** Co-immunoprecipitation analysis of MBD2–Kat7 interaction in HEK cells. GFP-tagged MBD2a, MBD2b, MBD2c, or GFP as a control were immunoprecipitated using GFP-binder beads. Pull-down fractions were analyzed by western blot using anti-RFP. Input and bound fractions are shown. Dashed red boxes highlight the absence of pull-down.

Beyond MBD2-NuRD complex, we examined the potential interplay between MBD2 and other chromatin (de)acetylation enzymes that are specifically recruited (Hdac11) or excluded (Kat7) from HC condensates in the presence of additional MBD2 condensates. For this purpose, we performed fluorescence microscopy and colocalization analysis as well as coimmunoprecipitation (CoIP) analysis following co-transfection with plasmids coding for GFP-tagged MBD2 and RFP-tagged Hdac11 or Kat7 (Fig. 8). For CoIP, GFP-tagged proteins from cell lysates were immunoprecipitated using a GFP-binder nanobody [36], and precipitated proteins were analyzed by western blot. Fluorescence microscopy analysis revealed that MBD2a and MBD2b, but not MBD2c, enriched Hdac11 locally (with partial recruitment into MBD2a subnuclear foci) (Fig. 8A). CoIP assays further validated the interaction of Hdac11 with MBD2a and MBD2b but not MBD2c (Fig. 8B). In contrast, Kat7 was selectively enriched into MBD2c condensates but not MBD2a or MBD2b subnuclear foci (Fig. 8C). However, neither of the three MBD2 isoforms were able to pull-down the Kat7 (Fig. 8D), indicating that the MBD2c–Kat7 interaction visualized under the microscope might be too weak to be detected by co-precipitation.

**Figure 9.**
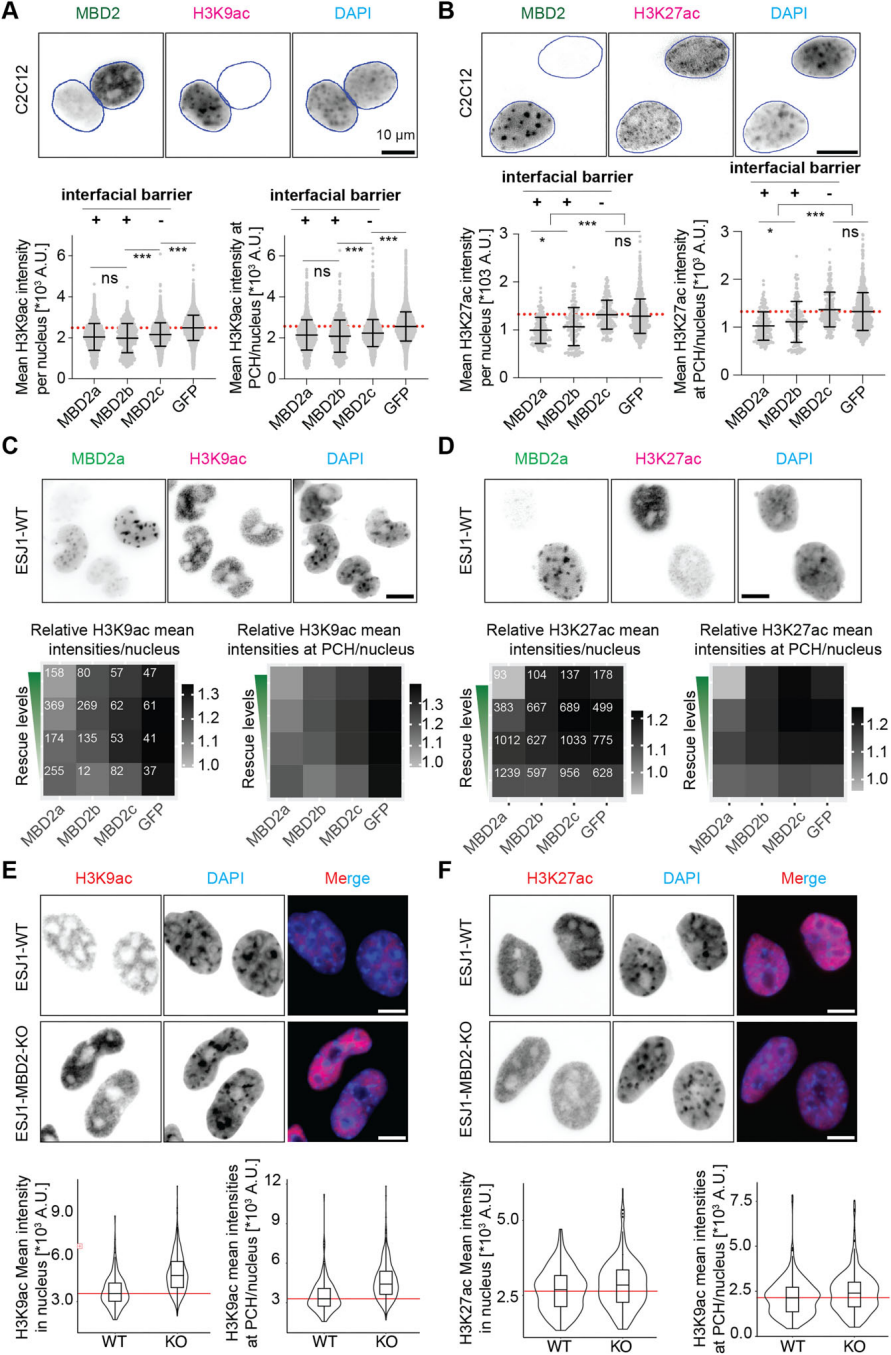
MBD2a/b decreases the histone acetylation levels in pericentric heterochromatin regions in both C2C12 (**A, B**) and ES (**C–F**) cells. (**A, B**) Representative images (top) and quantitative analysis of histone acetylation [H3K9ac **(A)**, H3K27ac **(B)**] abundance in whole nuclei and in pericentric heterochromatin (PCH) (bottom) in C2C12 cells 48 h after transfection of plasmid coding for GFP-MBD2 isoforms. Cells with similar expression levels of MBD2 isoforms were chosen. H3K9ac and H3K27ac were visualized by immunofluorescence staining after transfection. DNA was counterstained with DAPI. Images were taken using a Nikon Eclipse TiE2 microscope equipped with a Plan Apo λ 40× air objective. Data are represented as scatter plots with mean ± SD. The red dashed line indicates the mean fluorescence intensities in cells with GFP expression. For panel (A), *n* (MBD2a) = 172; *n* (MBD2b) = 145; *n* (MBD2c) = 205; *n* (GFP) = 919. For panel (B), *n* (MBD2a) = 1459; *n* (MBD2b) = 831; *n* (MBD2c) = 3016; *n* (GFP) = 5779. Significances were calculated by an unpaired *t*-test. ns *P* >.05; ***P ≤.001. Raw data can be found in Supplementary Table S10. (**C, D**) Representative images (top) and quantitative analysis of histone acetylation [H3K9ac **(C)**, H3K27ac **(D)**] abundance in whole nuclei and in pericentric heterochromatin (PCH) (bottom) in ES cells 48 h after transfection of plasmid coding for GFP-MBD2 isoforms. Cells were classified into four classes based on GFP intensities. Each class contains cells with similar GFP intensities. H3K9ac and H3K27ac were visualized by immunofluorescence staining after transfection. DNA was counterstained with DAPI. Images were taken using a Nikon Eclipse TiE2 microscope equipped with a Plan Apo λ 40× air objective. Data are represented as heat maps showing the mean values. Cell numbers in each condition were shown. Raw data can be found in Supplementary Table S10. (**E-F**) Representative images (top) and quantitative analysis of histone acetylation [H3K9ac **(E)**, H3K27ac **(F)**] abundance in whole nuclei and in pericentric heterochromatin (PCH) (bottom) in wild-type (WT) and MBD2 knockout (MBD2-KO) ES cells. H3K9ac and H3K27ac were visualized by immunofluorescence staining. DNA was counterstained with DAPI. Images were taken using a Nikon Eclipse TiE2 microscope equipped with a Plan Apo λ 40× air objective. Data are represented by violin plots embedded with box plots. Violin plots displayed the probability density of the data at different values, mirrored around the center line. The box plots indicated the median (central line), interquartile range (IQR) (box), and whiskers representing 1.5 × IQR. For panel (E), *n* (WT) = 318; *n* (MBD2-KO) = 255. For panel (F), *n* (WT) = 148; *n* (MBD2-KO) = 186. Raw data can be found in Supplementary Table S10.

Given the differential effects of MBD2 isoforms on the localization of histone (de)acetylation machinery, we checked their influences on histone acetylation levels in pericentric heterochromatin compartments in C2C12 and ES cells (KO and upon re-expressing the MBD2 isoforms) following transfection and immunofluorescence staining (Fig. 9). Consistently, overexpression of MBD2a and MBD2b, but not MBD2c, decreased the acetylation levels at histone H3 lysine 27 and lysine 9 (H3K27ac and H3K9ac) in pericentric heterochromatin regions in a concentration-dependent manner (Fig. 9A–D). Consistently, MBD2 depletion increased the H3K27ac and H3K9ac levels at pericentric heterochromatin compartments (Fig. 9E–F).

Altogether, we found that MBD2a and MBD2b, but not MBD2c, form spherical condensates *in vitro*, establish apparent interfacial barriers surrounding membraneless pericentric heterochromatin compartments *in vivo*, and consequently result in lower acetylation states, reduced chromatin accessibility, and diminished transcriptional activity.

## Discussion

Several publications have suggested liquid-like properties of various membraneless organelles *in vivo*, with complex and distinct protein compositions. However, only a small fraction of these proteins seem to be essential for condensate formation. In this study, we employed a novel and comprehensive approach combining spatial proteomics (nuclei fractionation) with *in vitro* phase separation and advanced phase separation prediction tools to uncover the mechanisms underlying heterochromatin compartmentalization, with a particular focus on pericentric heterochromatin. Firstly, utilizing heterochromatin fractions isolated from mouse brain, we demonstrated that heterochromatin undergoes phase separation *in vitro*, selectively enriching specific proteins within condensates while simultaneously excluding others. This suggests that heterochromatin forms dynamic and phase-separated condensates rather than simply indiscriminate protein aggregation. However, realizing that not all proteins contribute equally to heterochromatin phase separation, we employed multiple phase separation prediction tools (DrLLPS, PSAP, and PhaSePred) to identify the candidate phase separation scaffold proteins (scaffolds) across the mouse proteome. These scaffolds are capable of phase separation by either self-assembling (SaPS) or partner-dependent mechanisms (PdPS). By intersecting these predictions with the heterochromatin phase separation proteome data, we identified ∼250 potential phase separation scaffolds at heterochromatin out of ∼1000 proteins found in heterochromatin condensates. This further supports the hypothesis that phase separation is a driving force underlying heterochromatin compartmentalization. Notably, 20 proteins were predicted to modulate pericentric heterochromatin compartmentalization based on their known subcellular localizations, including the MBD2 protein, a member of the NuRD complex involved in various cellular processes, including embryonic stem (ES) cell differentiation [47, 57] and carcinogenesis [58, 59].

MBD2 has been reported to exist in three isoforms due to alternative splicing and translation start sites. All isoforms contain the highly conserved MBDTRD but retain either the amino terminus (MBD2c, termed MBD2∆C here) or the carboxyl terminus (MBD2b, termed MBD2∆N here) or both (MBD2a, termed MBD2 here). The three isoforms exhibit different binding partners and functions [47]. Previous work indicated that MBD2c is expressed in ES cells and maintains the pluripotent state, while MBD2a/b are increasingly expressed during differentiation [47]. Thus, here we investigated the contributions of the different MBD2 domains—amino terminus, MBDTRD, and carboxyl terminus. Through a combination of *in vitro* phase separation assays, *in silico* analyses and experiments in cells, we demonstrated that MBD2 undergoes LLPS via the CC domain-mediated oligomerization, forming liquid-like condensates that are likely critical for pericentric heterochromatin organization and dynamics. In addition, the glycine/arginine (GR) repeat region in the amino terminus of MBD2 modulates condensate morphology via preferential intra-molecular interactions. Our data suggest a model wherein MBD2 first seeds at the pericentric heterochromatin region by binding to methylated DNA through its MBD domain, thus, locally enriching MBD2 concentration and surpassing the LLPS concentration threshold. This seeding event increases the MBD2 local concentration and, thus, enhances the MBD2 oligomerization. The latter leads to the formation of liquid-like condensates with apparent interfacial barriers that subsequently modulate the composition and promote overall low acetylation levels at pericentric heterochromatin by recruiting as well as excluding other proteins within these structures as shown by our data. As a result, MBD2 phase separation increases the compaction of heterochromatin, reduces the accessibility of various active molecular machineries, such as transcription complexes, to DNA repeats, and finally limits the activities of these repeats, including their transcriptional levels.

Our study also highlights the nuanced differences between self-assembling phase separation (SaPS, or LLPS) and partner-dependent phase separation (PdPS, or PPPS), with MBD2 exemplifying how a protein can leverage both mechanisms. While LLPS is driven by homotypic interactions and generates interfacial barriers, PPPS involves multivalent interactions with other macromolecules, such as nucleic acids [40, 53, 52] and does not form interfacial barriers. The interplay between the two types of phase separation likely contributes to a more precise fine-tuning of chromatin architecture and function, adding another layer of complexity to our understanding of nuclear chromatin organization.

In summary, we developed an integrated platform combining nuclei fractionation, *in vitro* phase separation, mass spectrometry, phase separation and protein structure predictions, and functional assays to identify key phase separation scaffolds involved in pericentric heterochromatin compartmentalization and to provide mechanistic insights. These findings pave a new way for future studies aimed at deciphering the specific roles of phase separation scaffolds in chromatin dynamics and their implications in gene regulation and genome stability.

## Supplementary Material

gkaf1380_Supplemental_Files

## Data Availability

The mass spectrometry proteomics data have been deposited to the ProteomeXchange Consortium via the PRIDE [60] partner repository with the dataset identifier PXD055626 at https://www.ebi.ac.uk/pride/. All image data have been deposited and are available at https://tudatalib.ulb.tu-darmstadt.de/handle/tudatalib/4431.
