# Correction to ‘Heterochromatome wide analyses reveal MBD2 as a phase separation scaffold for heterochromatin compartmentalization and composition’

**DOI:** 10.1093/nar/gkag032

**Published:** 2026-01-16

This is a correction to: Hui Zhang, Enes Ugur, Christian Hake, Hector Romero, Maria Arroyo, Marah Mahmoud, Frederik Lermyte, Heinrich Leonhardt, M Cristina Cardoso, Heterochromatome wide analyses reveal MBD2 as a phase separation scaffold for heterochromatin compartmentalization and composition, Nucleic Acids Research, Volume 53, Issue 22, 11 December 2025, gkaf1380, https://doi.org/10.1093/nar/gkaf1380

In the originally published version of this article, an incorrect Author Contributions statement was inserted during the typesetting process. This error has now been corrected, and the article has been updated to include the accurate Author Contributions statement as provided by the authors. The publisher apologizes for this error.

