# Supplementary material for: Heterochromatome wide analyses reveal MBD2 as a phase separation scaffold for heterochromatin compartmentalization and composition
Source: Nucleic Acids Res. 2025 Dec 19;53(22):gkaf1380. doi: 10.1093/nar/gkaf1380 (PMC12715500; doi:10.1093/nar/gkaf1380)
Supplement: gkaf1380_Supplemental_Files [file gkaf1380_supplemental_files.zip › Zhang et al_supplementary.pdf]

Heterochromatome wide analyses reveal MBD2 as a phase separation scaffold for heterochromatin compartmentalization and composition

Hui Zhang[1], Enes Ugur[2,3], Christian Hake[4], Hector Romero[1], Maria Arroyo[1], Marah Mahmoud[1], Frederik Lermyte[4], Heinrich Leonhardt[2], M. Cristina Cardoso[1,*]

[1] Cell Biology and Epigenetics, Department of Biology, Technical University of Darmstadt, Germany
[2] Human Biology and Bioimaging, Faculty of Biology, LMU Munich, Germany
[3] Department of Proteomics and Signal Transduction, Max-Planck Institute of Biochemistry, Martinsried, Germany
[4] Clemens-Schöpf Institute of Organic Chemistry and Biochemistry, Department of Chemistry, Technical University of Darmstadt, Germany
* Correspondence M. Cristina Cardoso; Tel: +49-6151-1621882; Email: cardoso@bio.tu-darmstadt.de

SUPPLEMENTARY FIGURES

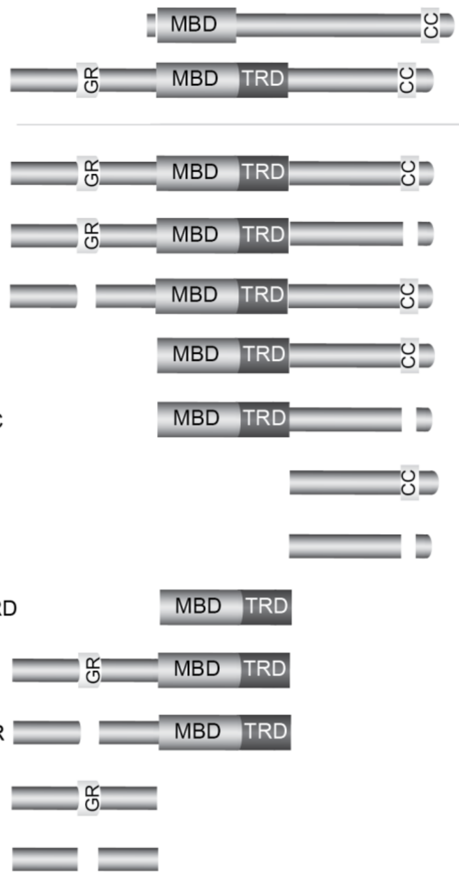

Construct name	Protein Length /aa	Mammalian Expression **pc number**	Mammalian Expression **addgene**	Bacteria Expression **pc number**	Bacteria Expression **addgene**
MBD3	285			pc4784	229749
MBD2	414	pc2399	211572	pc4786	229751
MBD2 **MBD2a**	414	pc2399	211572	pc4786	229751
MBD2ΔCC	414	pc5088	229764	pc5081	229760
MBD2ΔGR	414	pc5085	---	pc5078	229758
MBD2ΔN **MBD2b**	262	pc2068	229560	pc4787	229752
MBD2ΔNΔCC	228	pc5089	229765	pc5082	232745
MBD2-C	179	pc2841	229561	pc4793	229756
MBD2-CΔCC	145	pc5091	229766	pc5084	229761
MBD-MBDTRD	83	pc2843	229562	pc4791	229754
MBD2ΔC **MBD2c**	235	pc4794	229757	pc4792	229755
MBD2ΔCΔGR	235	pc5086	229762	pc5079	229759
MBD2-N	152	pc2067	229559	pc4789	229753
MBD2-NΔGR	135	pc5087	229763	pc5083	230974

Figure S1. Scheme summarizing the structures and plasmids of MBD3 and MBD2 truncations. MBD: methyl-CpG binding domain; TRD: transcriptional repression domain. G/R: glycine/arginine; CC: coiled coil domain. pc: plasmid collection.

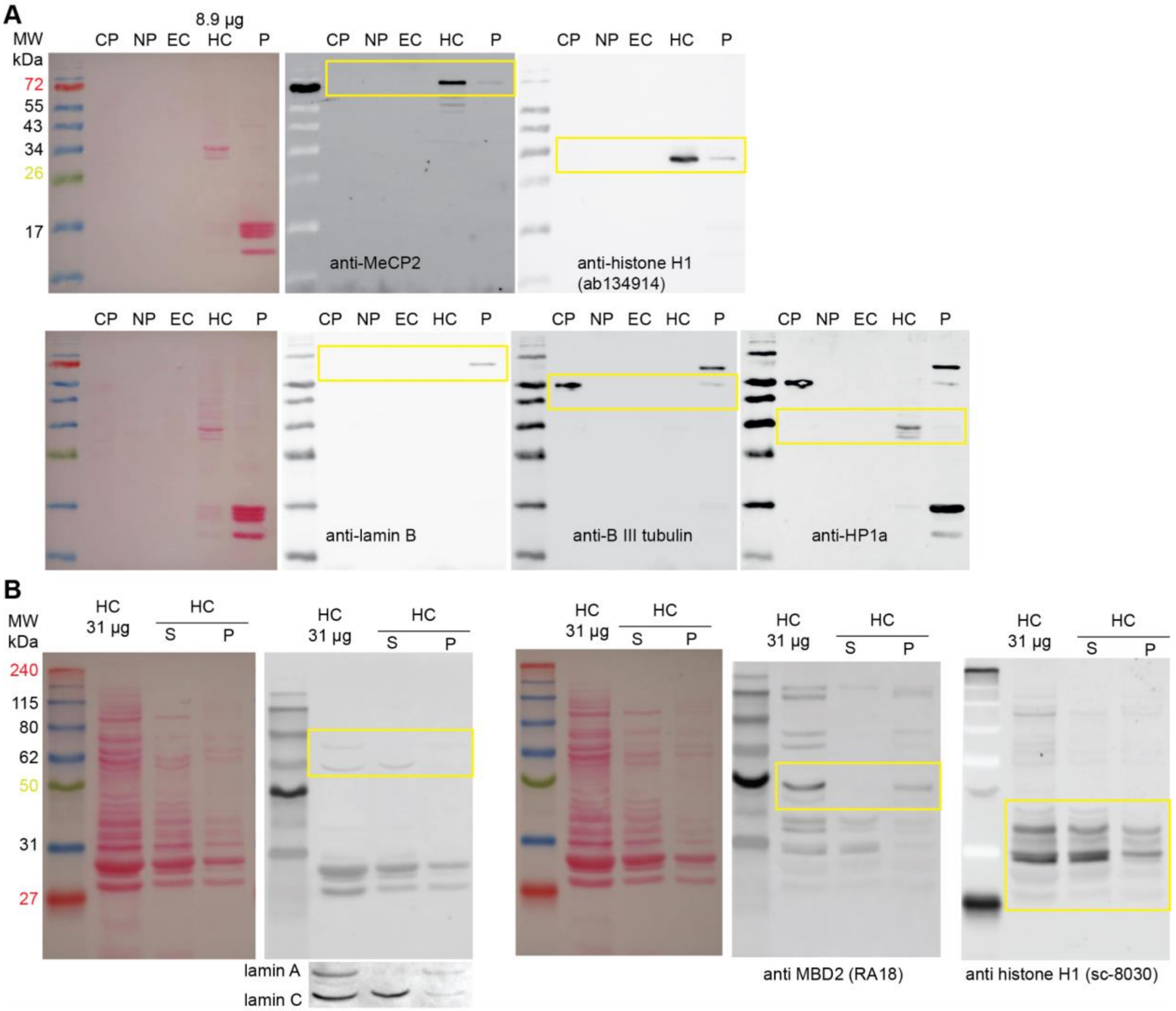

Figure S2. Western blot detection of protein distribution after nuclei fractionation (**A**) and heterochromatin phase separation (**B**). (**A**) Full Western blot of marker proteins for cytoplasm (beta **III** tubulin), heterochromatin (HP1 alpha, MeCP2 and histone H1) and insoluble nuclear proteins (lamin B), corresponding to **Figure 1A**. (**B**) Full Western blot of the detection of lamin A/C, MBD2 and histone H1 isoforms (H1s) in phase-separated condensates, corresponding to **Figure 1F**. A longer exposure of the lamin A/C is shown below the respective blot.

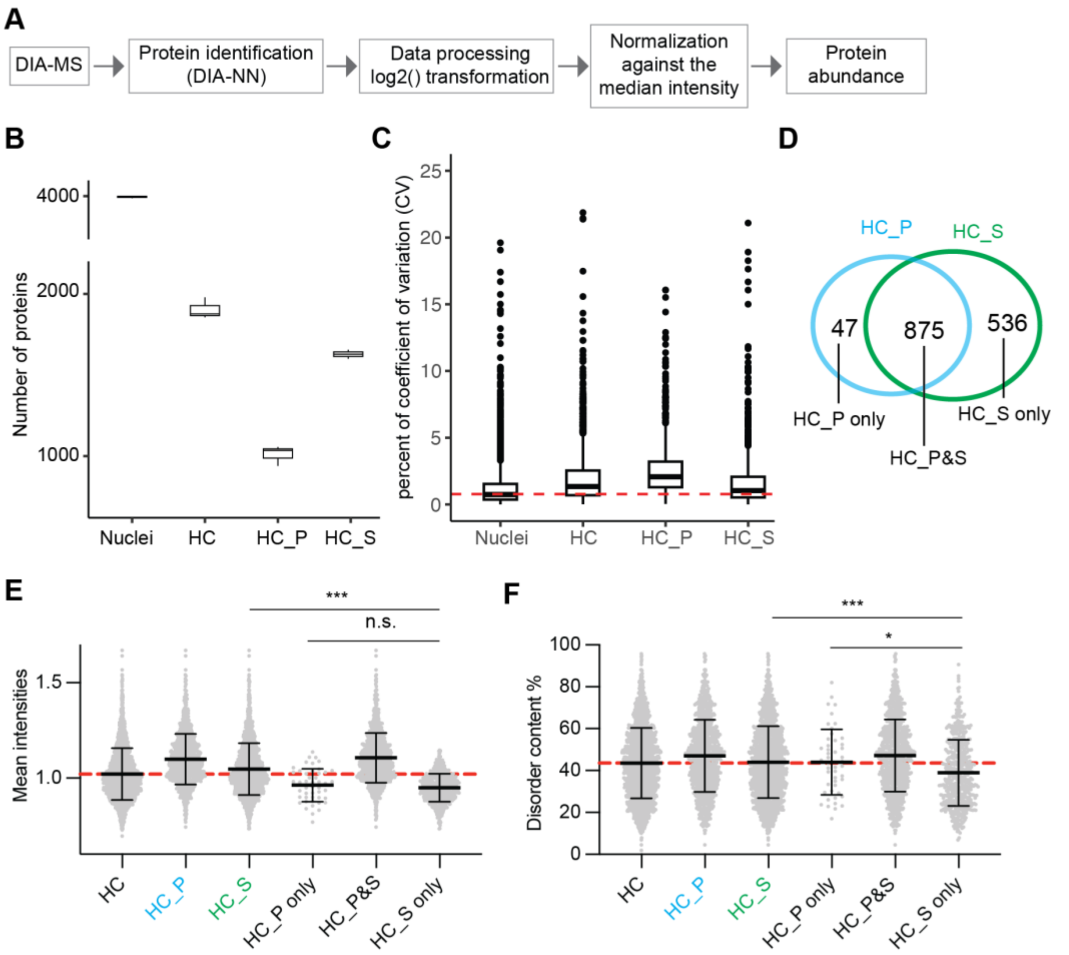

Figure S3. Mass spectrometry data analysis. (**A**) DIA-MS data analysis pipeline. DIA-MS data were uploaded onto DIA-NN software, followed by protein identification, and data processing (log transformation and normalization). (**B**) Box plot of protein numbers in each measurement. The box plots indicate the median (central line), interquartile range (IQR) (box), and whiskers representing 1.5 × IQR. n = three replicates. Raw data can be found in Table S10. (**C**) Box plot of protein variances among the three replicates of each fraction. The red dashed line represents the mean values of percent of coefficient of variation (CV) in nuclei. The box plots indicated the median (central line), interquartile range (IQR) (box), and whiskers representing 1.5 × IQR. n = three replicates. Raw data can be found in Table S10. (**D**) Venn diagram showing the overlap of proteins identified in supernatant (S) and pelleted condensates (P) following heterochromatin fraction (HC) phase separation, centrifugation, and mass spectrometry measurements. Proteins found exclusively in condensates and supernatants were named HC_P only and HC_S only, respectively, while the ones in both measurements were named HC_P&S. (**E-F**) Scatter plot showing the relative abundance (**E**) and the disorder scores (**F**) of the proteins recognized in different fractions. Data are shown with Mean ± SD. The red dashed line indicates the mean fluorescence intensities (**E**) and the mean disorder score (**F**) in the whole heterochromatin fraction. n (HC) = 1805; n (HC_P) = 915; n (HC_S) = 1403; n (HC_P only) = 47; n (HC_P&S) = 868; n (HC_S only) = 535. Significances were calculated by an unpaired t-test. n.s. no significance, P > 0.05; *P ≤ 0.05; ***P ≤ 0.001. Raw data can be found in Table S10.

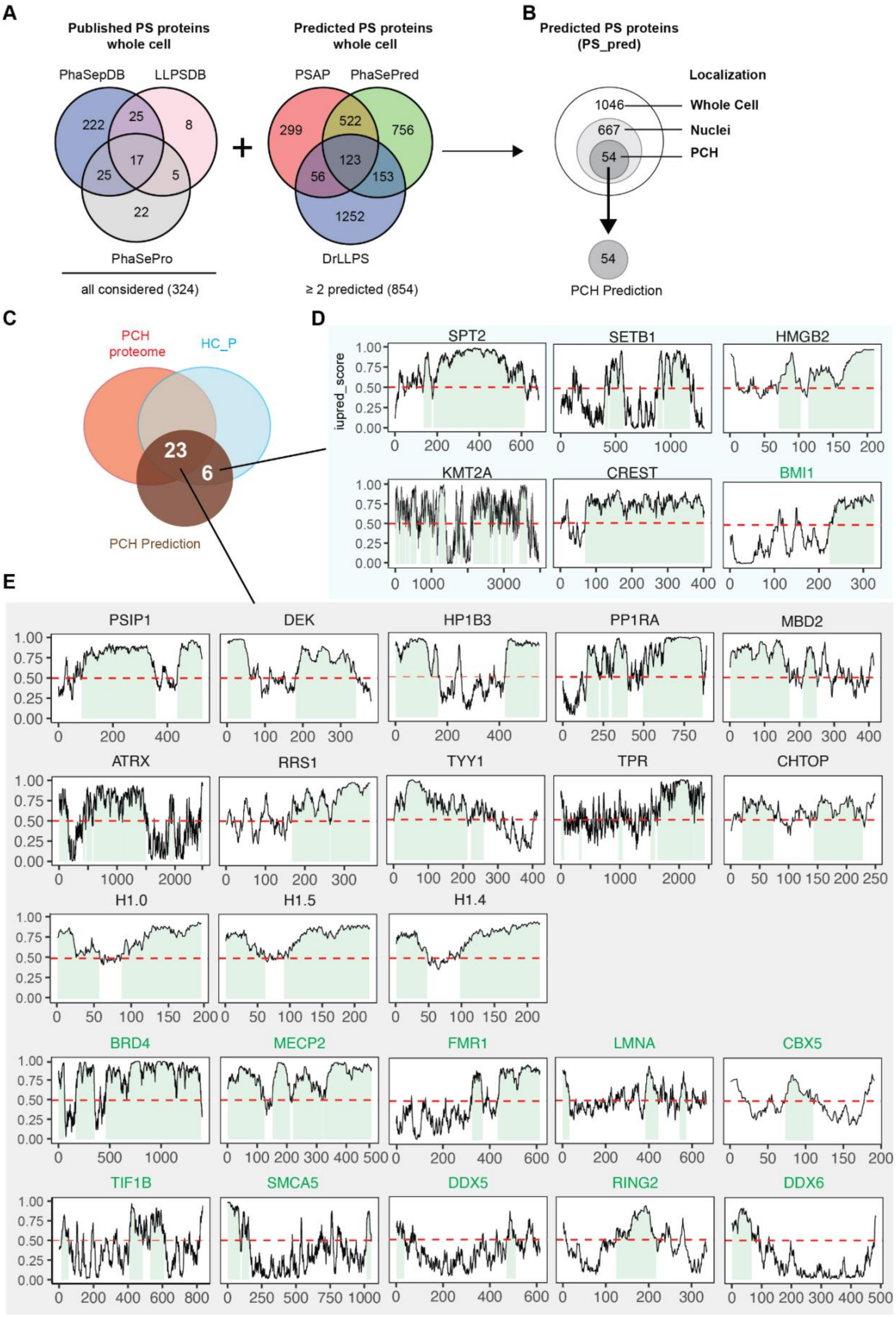
A
Published PS proteins whole cell
PhaSepDB
LLPSDB
PhaSePro
222
25
8
17
25
5
22
all considered (324)
+
Predicted PS proteins whole cell
PSAP
PhaSePred
DrLLPS
299
522
756
123
56
153
1252
≥ 2 predicted (854)
B
Predicted PS proteins (PS_pred)
Localization
1046
Whole Cell
667
Nuclei
54
PCH
54
PCH Prediction
C
PCH proteome
HC_P
23
6
PCH Prediction
D
iupred_score
SPT2
SETB1
HMGB2
KMT2A
CREST
BMI1
E
PSIP1
DEK
HP1B3
PP1RA
MBD2
ATRX
RRS1
TYY1
TPR
CHTOP
H1.0
H1.5
H1.4
BRD4
MECP2
FMR1
LMNA
CBX5
TIF1B
SMCA5
DDX5
RING2
DDX6

Figure S4. Prediction of potential phase separation scaffold proteins in proteome-wide and pericentric heterochromatin. (**A**) Overlap among datasets of published (left) and predicted (right) phase separation scaffold proteins in mice. For the PhaSePred datasets, the phase separation score of the whole mouse proteome was downloaded and the top 1000 proteins that possibly self-assemble (SaPS) or partner-dependent-assemble (PdPS) to form condensates were considered (22). For the PSAP, the phase separation scores of the whole mouse proteome were predicted using the scripts from van Mierlo, Guido et al. (23). The top 1000 proteins were considered. For DrLLPS, the candidate phase separation proteins in mice were downloaded directly (24). Only proteins predicted in ≥ two predictors were considered candidate phase separation scaffold proteins. (**B**) Venn diagram showing the number of published and predicted phase separation scaffold proteins in mouse whole cell, nuclei, and pericentric heterochromatin regions. (**C**) Venn diagram showing the overlap of three independent proteome-wide data and highlighting the most likely phase separation (PS) scaffold proteins in pericentric heterochromatin (PCH) as shown in **Figure 2B**. (**D-E**) Line profile showing the disorder prediction of proteins recognized in pericentric heterochromatin regions using IUPred2A (https://iupred2a.elte.hu/). >0.5 (red dashed line) was considered disordered. Long disordered regions with contiguous disorder segments of >30 amino acids (aa) were highlighted with light green. Published candidates were labeled with green. (**D**) Candidate PCH scaffolds recognized in only two proteomes (PCH (scaffold) prediction, HC_P proteome). (**E**) Candidate PCH scaffolds recognized in all three proteomes (PCH proteome, PCH (scaffold) prediction, HC_P proteome).

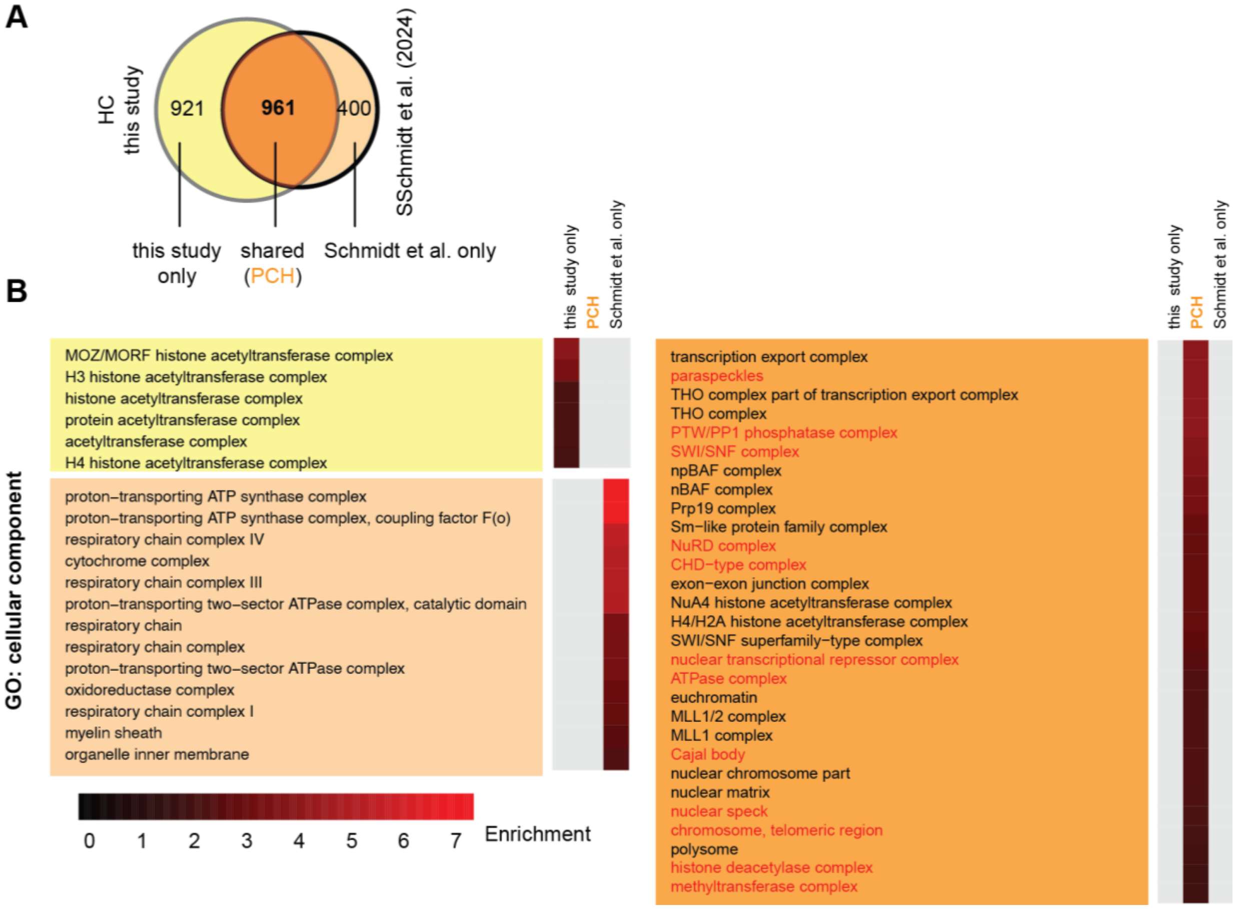

Figure S5. Comparison of the heterochromatin proteomes from two independent strategies. (**A**) Venn diagram showing the overlap of proteins recognized in HC fraction in this study and Schmidt et al. (2024) (8). Proteins exclusively recognized in either dataset were named this study only or Schmidt et al. only, respectively. Proteins recognized in both were called shared (or PCH). (**B**) Gene ontology (GO) analysis of the proteins in each subclass as mentioned in (**A**). The protein list was subjected to the GOrilla tool (68) for gene ontology analysis in the categories of cellular components. The proteins recognized in the whole nuclei were applied as the background list. GO terms with a FDR q-value ≤0.05 and enrichment ≥2 were considered. The GO terms for cytoplasmic, RNA, ribosome, and nuclear membrane were removed manually. The heatmap indicates the enrichment levels. Heterochromatin, chromatin inactivation, and subnuclear membraneless organelles-related GO terms are highlighted with red.

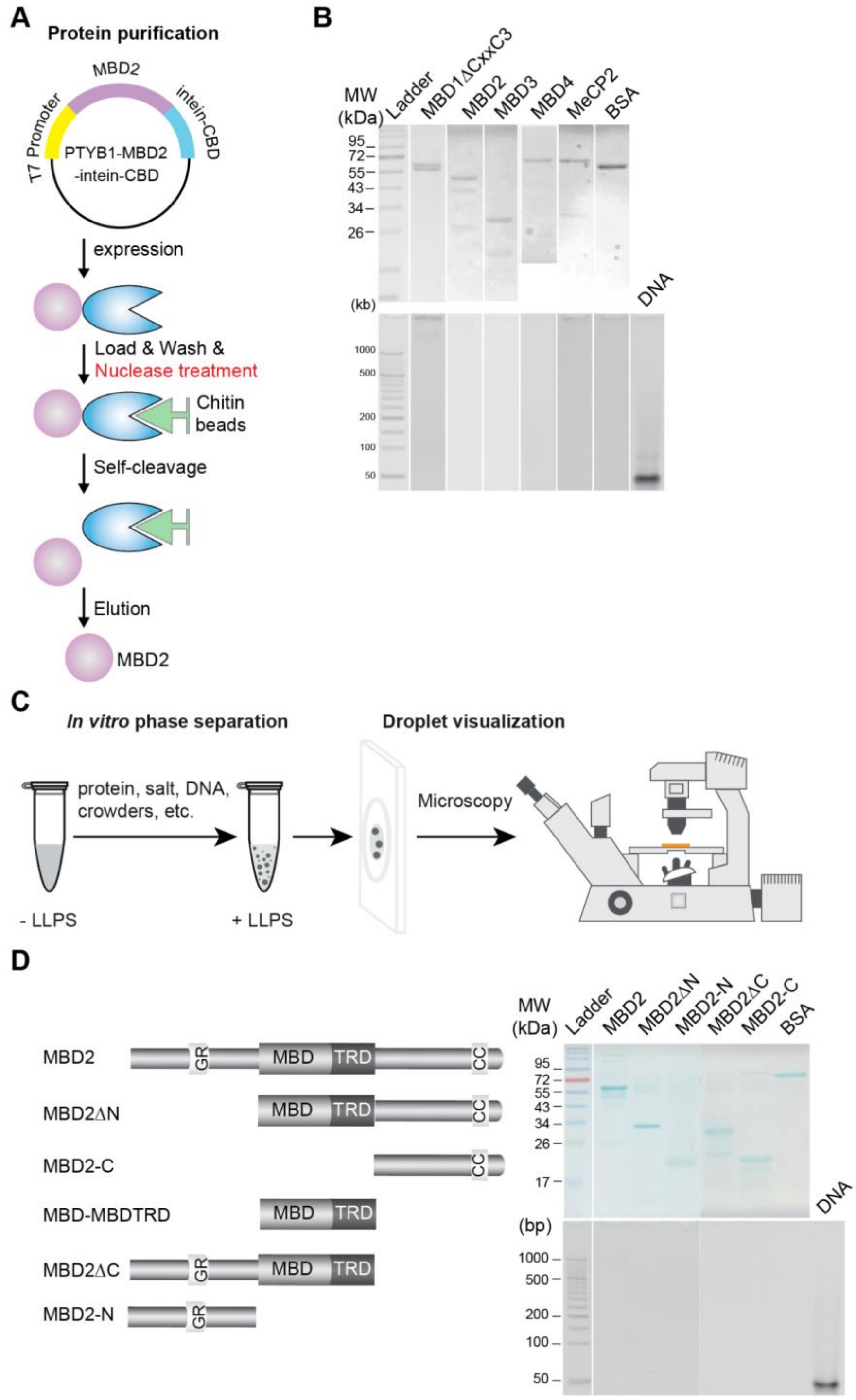

Figure S6. Scheme of protein purification and in vitro phase separation assay. (**A**) Schematic Illustration of intein-CBD-mediated protein purification. The protein coding sequence was fused to the amino terminus of intein-CBD (chitin-binding domain). Protein was produced in bacteria and immobilized to chitin beads, followed by nuclease treatment to remove possible DNA/RNA contaminations. The untagged protein was released by intein-mediated self-cleavage. (**B**) Validation of protein purity. The purity and DNA/RNA contaminations of purified MBD-containing proteins were detected by Coomassie blue staining (top, 2 μg except for MBD3 (4 μg)) and ethidium bromide (EtBr) (bottom, 10 μg) staining, respectively. Negative

control: BSA with the same amount. Positive control: ~140 ng of 42 bp dsDNA. (**C**) Schematic graph of in vitro phase separation assay. Purified protein was diluted in buffers at different conditions and incubated at room temperature for 45 min. Then, the mixtures were transferred to glass slides for microscopic imaging. (**D**) Validation of protein purity. Left: Schemed protein structures. Right: Protein purity and DNA/RNA contaminations were detected by coomassie blue staining (top, 2 μg) and ethidium bromide (EtBr) (bottom, 10 μg) staining, respectively. Negative control: BSA with the same amount. Positive control: ~140 ng of 42 bp dsDNA. GR: glycine/arginine rich region; MBD: methyl-CpG binding domain; TRD: transcriptional repression domain; CC: coiled coil domain.

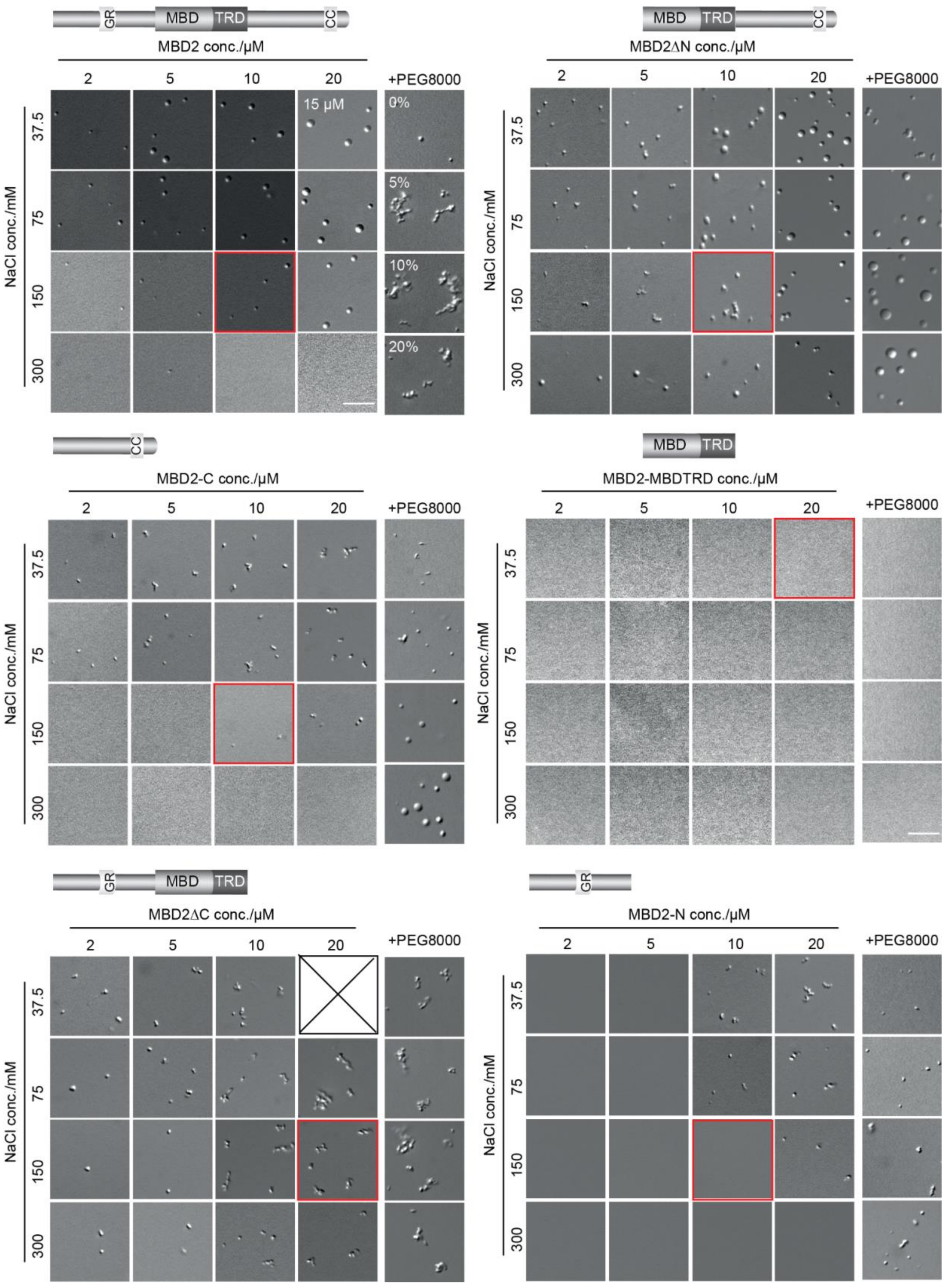
GR
MBD
TRD
CC
MBD2 conc./μM
2
5
10
20
+PEG8000
15 μM
0%
5%
10%
20%
NaCl conc./mM
37.5
75
150
300
MBD2ΔN conc./μM
MBD2-C conc./μM
MBD2-MBDTRD conc./μM
MBD2ΔC conc./μM
MBD2-N conc./μM

Figure S7. The phase separation properties of MBD2 truncations at the indicated salt/protein/crowding concentrations. The in vitro phase separation assay was done at different protein and salt concentrations. The mixtures were transferred to chambers made of double-sided tapes and sealed with coverslips 45 min after incubation at room temperature. The droplets were observed using a Nikon Eclipse TiE2 microscope equipped with differential interference contrast (DIC) microscopy. The red boxes indicate the protein/salt conditions used to check the influences of crowding agents. n = three replicates. Scale bars = 10 μm. conc.: concentration.

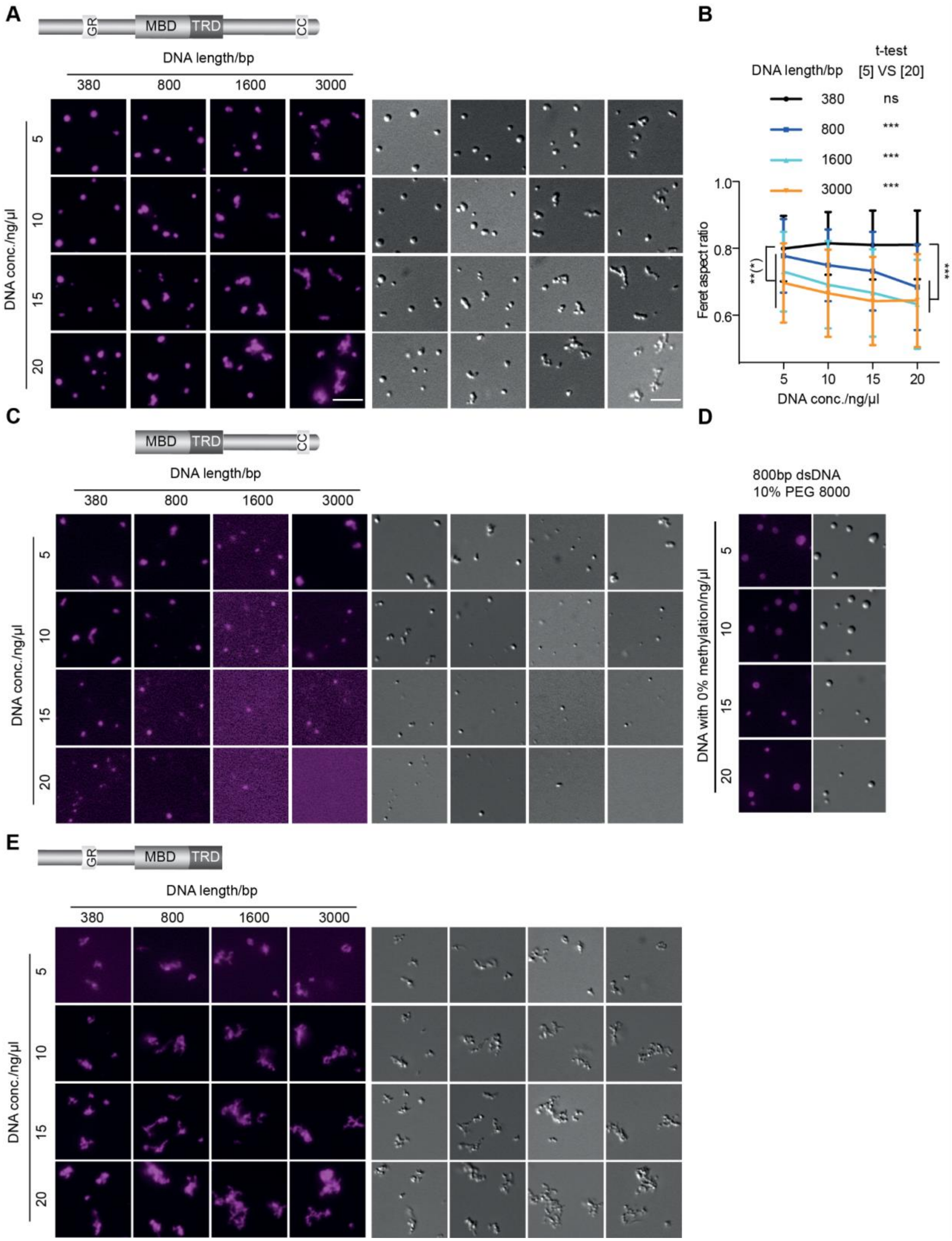

Figure S8. The phase separation properties of MBD2 truncations in the presence of DNA. The in vitro phase separation assay was done by incubating 10 μM purified MBD2, MBD2ΔN, and MBD2ΔC protein

with various conditions of DNA (length/concentrations) and 150 mM NaCl, followed by microscopic imaging as described in **Figure S7**. DNA was visualized by DRAQ5 dyes. Scale bars = 10 μm. conc.: concentration. n = three replicates. (**A, C-E**) Representative differential interference contrast (DIC) and fluorescent images showing the phase separation properties of different MBD2 constructs and DNA distributions. (**B**) Quantitative analysis showing the influences of DNA length and concentration on the MBD2 condensate morphology, as shown in (**A**). condensates were segmented based on the fluorescent channel (DRAQ5). Condensate morphology was evaluated by the ferret aspect ratio. Results are shown with Mean ± SD. Raw data can be found in Table S10.

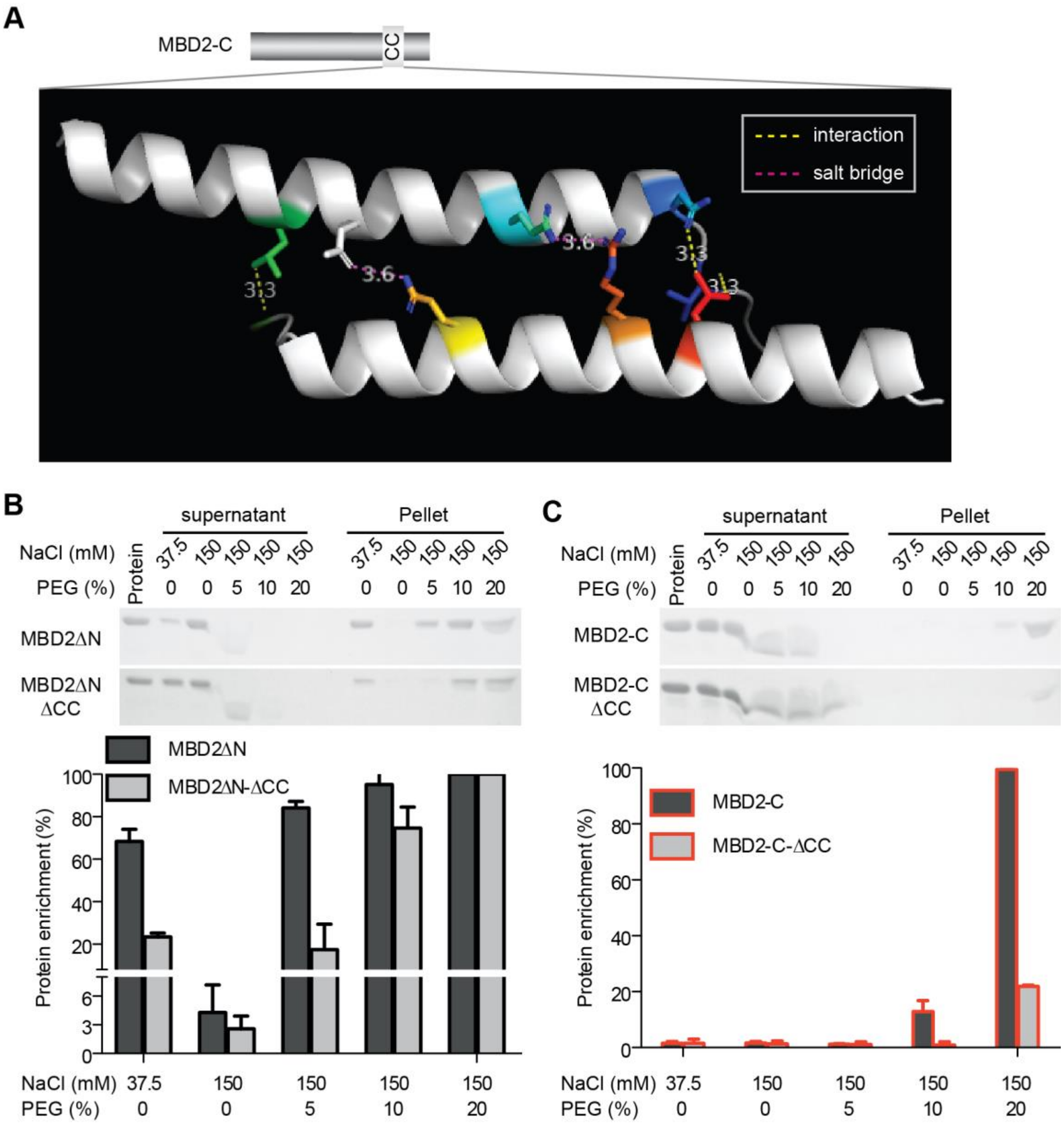

Figure S9. MBD2 spherical condensation is driven by coiled coil (CC) domain-mediated oligomerization. (**A**) Prediction of CC antiparallel dimerization by AlphaFold Server (https://alphafoldserver.com/). The indicated interactions and salt bridges between the two CC chains were labelled with yellow and pink dashed lines, respectively. (**B-C**) MBD2 condensate sedimentation and respective quantification. MBD2 constructs used are as in **B**. The phase separation mixtures with and without the crowding agent PEG 8000 were centrifuged to pellet the condensates. Clear supernatants and pellets were separated and collected, analyzed by SDS-PAGE followed by coomassie blue staining. According to Odom et al. (1997) (69), PEG 8000 binds SDS micelle and migrates in the SDS-PAGE, which shifts the SDS monomer–micelle equilibrium in favor of micelles and thus lowers the apparent critical micelle concentration (cmc) of SDS. As a result, proteins larger than 19-20 kDa (MBD2ΔN: 30kDa; MBD2-C: 19.7 kDa) migrate faster in the loading buffer which contains PEG 8000. The protein fractions in pellets relative to total MBD2 protein were quantitatively analyzed using FIJI. n = three biologically independent experiments. Data are represented as mean ± SD. Raw data can be found in Table S10.

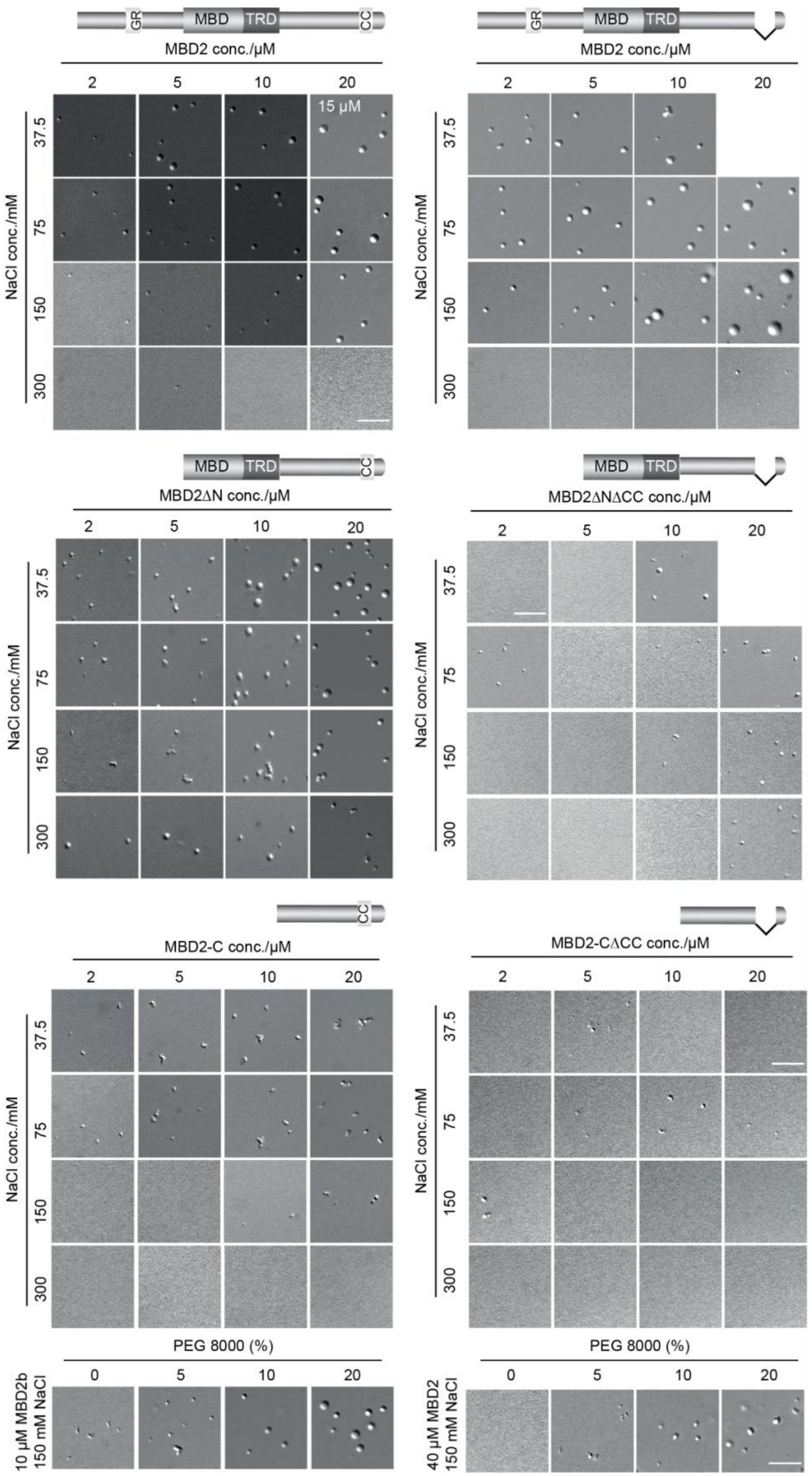

GR
MBD
TRD
CC
MBD2 conc./µM
2
5
10
20
15 µM
NaCl conc./mM
37.5
75
150
300
MBD2ΔN conc./µM
MBD2ΔNΔCC conc./µM
MBD2-C conc./µM
MBD2-CΔCC conc./µM
PEG 8000 (%)
0
10 µM MBD2b
150 mM NaCl
40 µM MBD2
150 mM NaCl

Figure S10. The contributions of the CC domain to MBD2 phase separation properties at the indicated salt/protein/crowding concentrations. The droplets were observed using a Nikon Eclipse TiE2 microscope equipped with differential interference contrast (DIC) microscopy. n = three replicates. Scale bars = 10 μm. conc.: concentration.

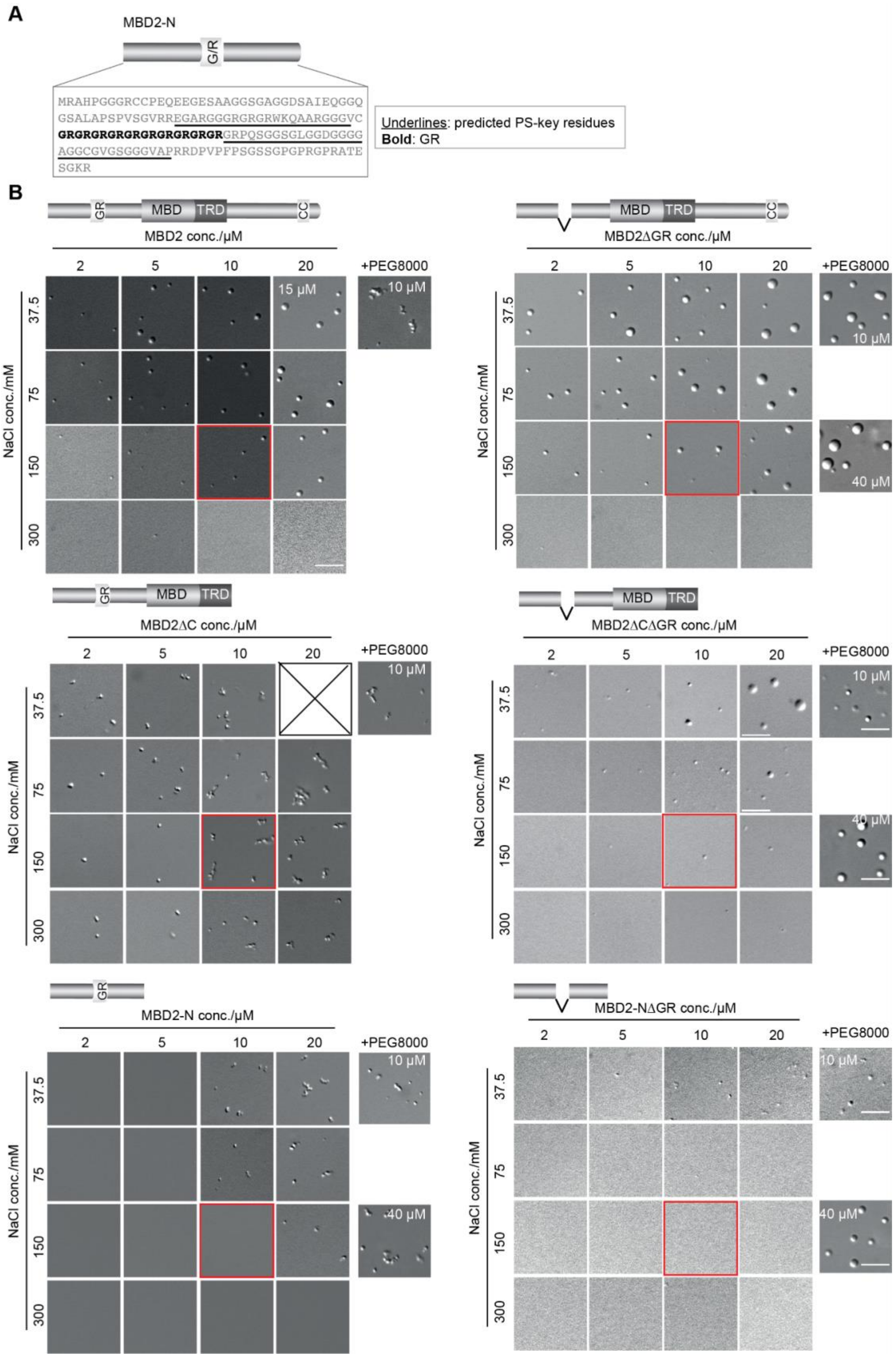
A
MBD2-N
G/R
MRAHPGGGRCCPEQEEGESAAGGSGAGGDSAIEQGGQ
GSALAPSPVSGVRREGARGGGRGRGRWKQAARGGGVC
GRGRGRGRGRGRGRGRGRGRGRPQSGGSGLGGDGGGG
AGGCGVGSGGGVAPRRDPVPFPSGSSGPGPRGPRATE
SGKR
Underlines: predicted PS-key residues
Bold: GR
B
GR
MBD
TRD
CC
MBD2 conc./μM
MBD2ΔGR conc./μM
MBD2ΔC conc./μM
MBD2ΔCΔGR conc./μM
MBD2-N conc./μM
MBD2-NΔGR conc./μM
2
5
10
20
+PEG8000
15 μM
10 μM
40 μM
NaCl conc./mM
37.5
75
150
300

Figure S11. MBD2 amino terminus promotes the phase transition. (**A**) Prediction of the key phase separation residues of the MBD2 amino terminus by PSPHunter (http://psphunter.stemcellding.org/). The glycine/arginine (G/R) repeat is highlighted in bold font. The predicted key residues are underlined. (**B**) Representative differential interference contrast (DIC) images showing the phase separation properties of different MBD2 constructs with and without GR. The droplets were observed using a Nikon Eclipse TiE2 microscope equipped with differential interference contrast (DIC) microscopy. n = three replicates. Scale bars = 10 μm. conc.: concentration.

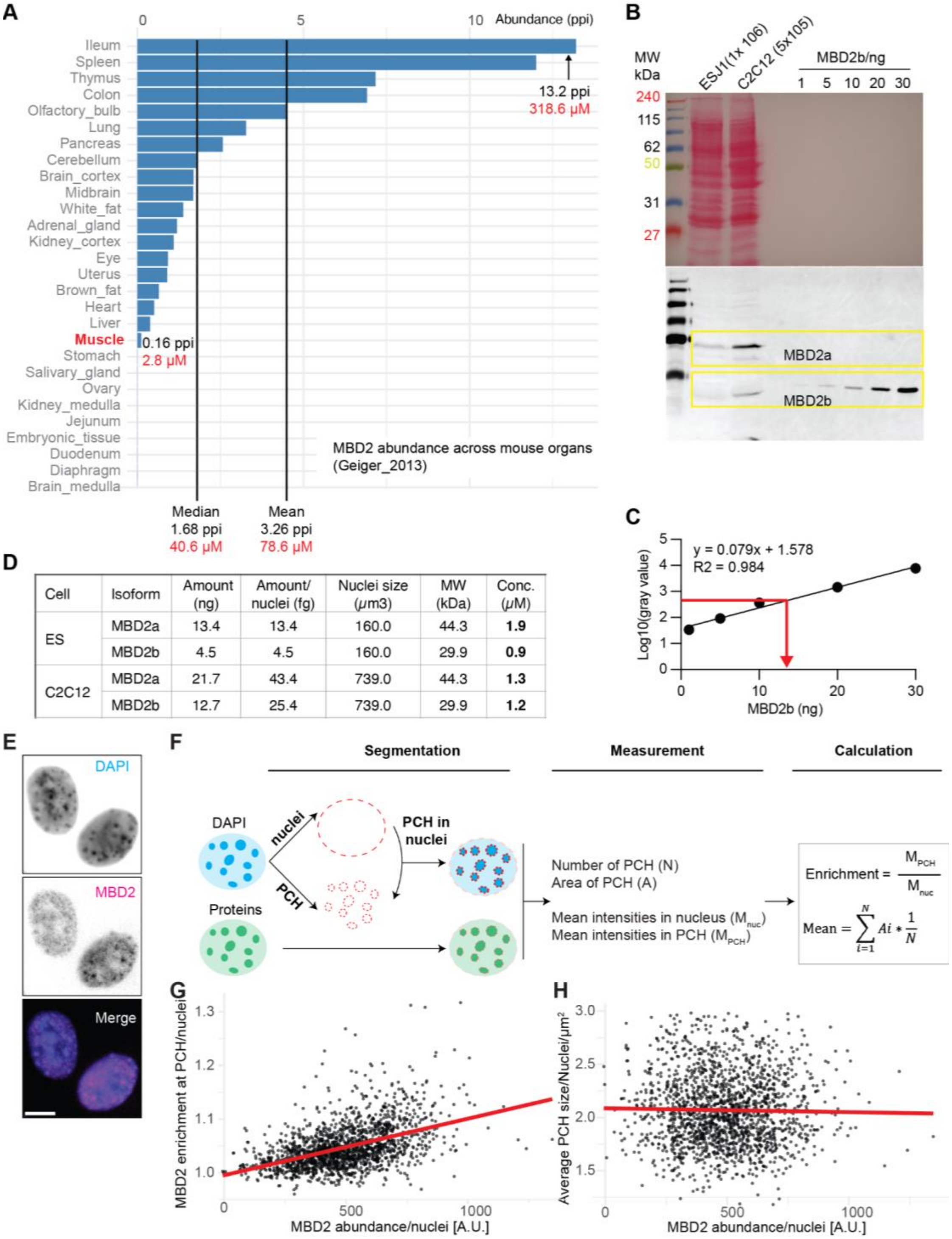

Cell	Isoform	Amount (ng)	Amount/ nuclei (fg)	Nuclei size (μm3)	MW (kDa)	Conc. (μM)
ES	MBD2a	13.4	13.4	160.0	44.3	**1.9**
	MBD2b	4.5	4.5	160.0	29.9	**0.9**
C2C12	MBD2a	21.7	43.4	739.0	44.3	**1.3**
	MBD2b	12.7	25.4	739.0	29.9	**1.2**

Figure S12. Quantitative analysis of endogenous MBD2 abundance and functions across mouse tissues and cultured cell lines. (**A**) Column plot depicting the relative abundance (ppi) of endogenous MBD2 across mouse tissues using the quantitative proteomic data from (54). Assuming a comparable MBD2 abundance in mouse muscle and C2C12 cells (mouse myoblast cells which differentiate into muscle) (**D**), we estimated the MBD2 molar concentration in muscle (≈ that in C2C12 cells, ~3 μM) and calculated MBD2 concentrations (mean, median, and maximum abundance) according to the ratios of relative abundance

(ppi) from the protein abundance in (54). (**B**) Western blot detection of endogenous MBD2 in C2C12 and in ES J1 cells whole nuclei lysates compared with a calibration set of purified MBD2b protein. MBD2 was recognized using anti-MBD2 antibody (RA-18, Table S5), followed by detection with a Cy5-conjugated secondary antibody. (**C**) Quantification of endogenous MBD2a/b based on the western blot results shown in (**B**). Grey values of purified MBD2b were measured using FIJI, log10() transformed and plotted against the corresponding protein amount. A linear trend line with an equation and an R-squared value is shown. (**D**) Table showing the endogenous abundance of MBD2a and MBD2b in C2C12 and in ES cells by western blot (**B**). (**E**) Representative images of endogenous MBD2 distribution in C2C12 cells. Endogenous MBD2 levels were detected by immunofluorescence staining using an antibody against the MBD domain of MBD2 and Cy5-conjugated secondary antibody (Table S5), followed by fluorescence microscopy. DNA was counterstained with DAPI. (**F**) Workflow to analyze the signal distributions in cultured cell nucleus and pericentric heterochromatin (PCH) using FIJI software. The cell nuclei and corresponding PCH were recognized based on the DAPI intensities. The number and size of PCH in each nucleus and the mean and sum fluorescence intensities in both nuclei and PCH were measured. The average PCH size per nuclei was calculated by dividing the total PCH size by PCH number per nucleus (N). The fold enrichment of fluorescence in PCH was calculated as the ratio of mean intensities in PCH (M_{PCH}) to those in the nucleus (M_{nuc}). (**G-H**) Scatter plot of MBD2 enrichment at PCH (**G**) and average PCH size (**H**) against endogenous MBD2 abundance with linear trendline (red). n = 1769. Raw data can be found in Table S10.

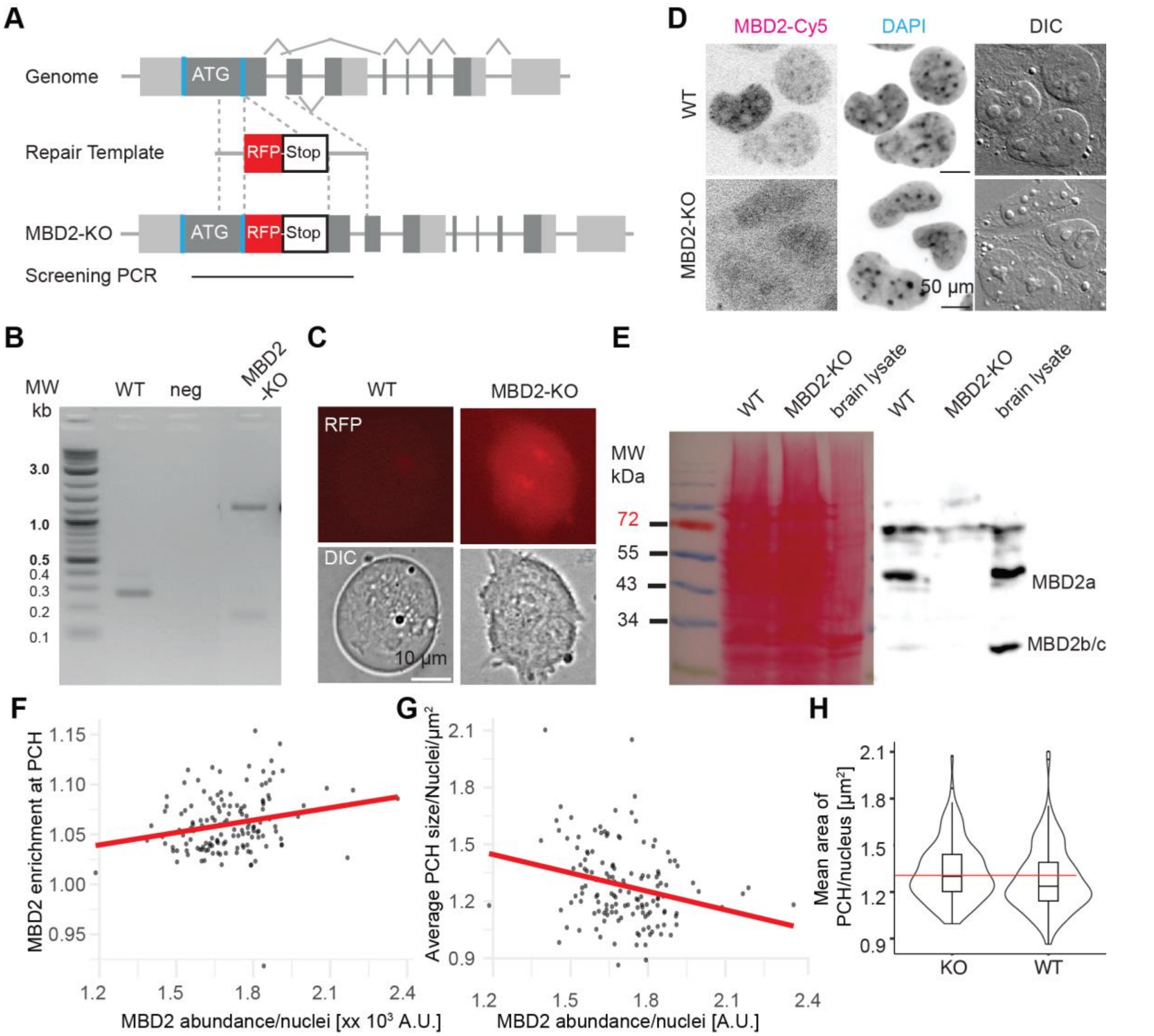

Figure S13. Generation and characterization of MBD2 knockout ES cell line. (**A**) Schematic graph showing the strategy of CRISPR-Cas9-mediated MBD2 triple knockout (all three isoforms were mutated). A double-strand break was induced at the second translational start site (the second blue line) by specific targeting of gRNA and recruitment of Cas9 to that region, followed by the homology repair using dsDNA containing RFP-poly(A) flanked by homology regions at the second translational start site. Blue line: translational start sites (ATG); Gray: coding region; Light gray; untranslated region. (**B-E**) Validation of MBD2 knockout ES cell line by PCR using genomic DNA as template (**B**), fluorescence microscopy (**C**), and immunofluorescence staining (**D**) and Western blot analysis (**E**) using anti-MBD2 antibody. neg: negative control with no PCR template. (**F-G**) Scatter plot of MBD2 enrichment at PCH (**F**) and average PCH size (**G**) against endogenous MBD2 abundance with linear trendline (red) in wild-type ES cells. n = 138. Raw data can be found in Table S10. (**H**) Violin plot embedded with box plot showing the function of endogenous MBD2 in PCH size in ES cells. The violin plot displays the probability density of the data at different values, mirrored around the center line. The box plot indicated the median (central line), interquartile range (IQR) (box), and whiskers representing the 1.5 × IQR. Red lines showed the median PCH size in MBD2 knock-out (KO) ES cells. n (WT) = 138; n (MBD2-KO) = 130. Raw data can be found in Table S10.

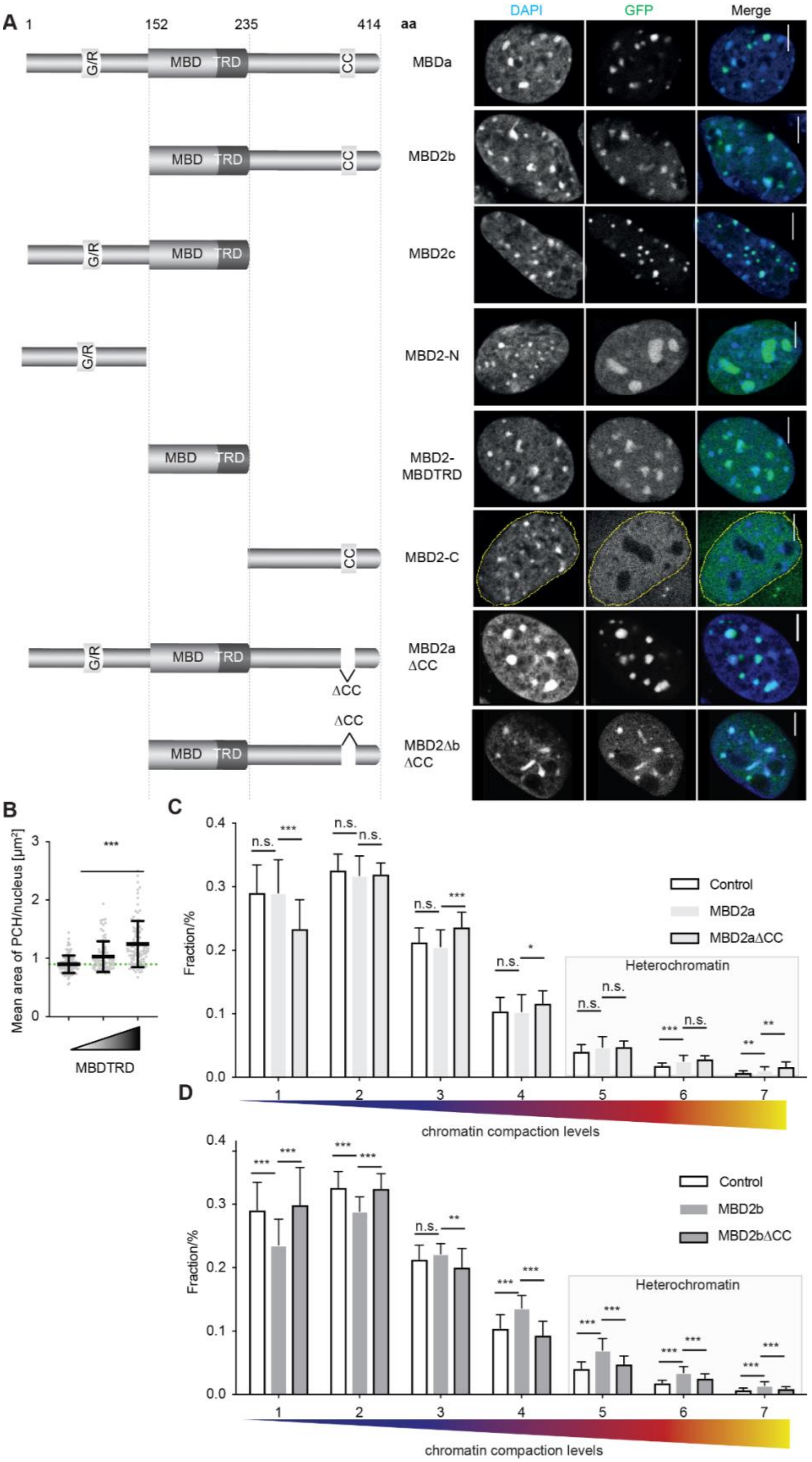
A
1
152
235
414
aa
G/R
MBD
TRD
CC
DAPI
GFP
Merge
MBDa
MBD2b
MBD2c
MBD2-N
MBD2-MBDTRD
MBD2-C
MBD2a ΔCC
ΔCC
MBD2Δb ΔCC
B
Mean area of PCH/nucleus [µm²]
MBDTRD
C
Fraction/%
Control
MBD2a
MBD2aΔCC
Heterochromatin
n.s.
chromatin compaction levels
D
Control
MBD2b
MBD2bΔCC

Figure S14. MBD2 modulates pericentric heterochromatin dynamics in an isoform and coiled coil domain-dependent manner in vivo. (**A**) Localization of ectopic GFP-MBD2 constructs in cultured mouse C2C12 cells. (**B**) Scatter plot showing the size of pericentric heterochromatin compartments with increased levels of the MBDTRD domain of MBD2. Cells were subgrouped into three groups based on the mean GFP intensities. Data are represented as mean ± SD. n (low) = 144; n (middle) = 81; n (high) = 106. Significances were calculated by unpaired t-test. ***P ≤ 0.001. Raw data can be found in Table S10. (**C-D**) Bar plot showing the effect of coiled coil domain on the compaction of chromatin in MBD2a (**C**) and MBD2b (**D**). The nucleus was classified into seven different chromatin compaction classes based on the DAPI intensities from DNA-free interchromatin region (class 1) to highly active and less compacted euchromatin (classes 2-4) and to highly compacted heterochromatin (classes 5-7) (as represented in **Figure 5E**). 3D images with similar ectopic MBD2 levels were taken and used for quantitative analysis. Data are represented as mean ± SD. n (control) = 33. n (MBD2a) = 32. n (MBD2aΔCC) = 30. n (MBD2b) = 34. n (MBD2bΔCC) = 22. Significances were calculated by unpaired t-test. n.s. no significance, P > 0.05; *P ≤ 0.05; **P ≤ 0.01; ***P ≤ 0.001. Raw data can be found in Table S10.

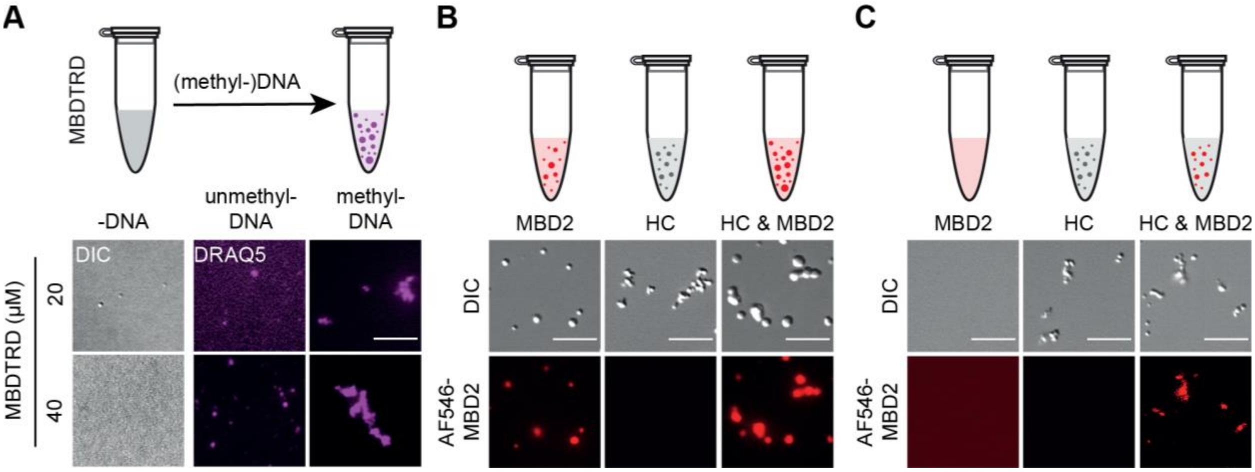

Figure S15. The phase separation properties of MBD2 constructs in the presence of DNA and heterochromatin fractions. (**A**) The methylated DNA drives the condensate formation of MBDTRD. The in vitro phase separation assay was performed by incubating 20 or 40 μM purified MBDTRD with 20 mg/μl 800 bp unmethylated or methylated DNA in a buffer containing 150 mM NaCl for 45 min at room temperature. The DNA was visualized by staining with the DNA dye DRAQ5. (**B-C**) Representative images showing the enrichment of MBD2 into heterochromatin condensates in the presence (**B**) and absence (**C**) of additional MBD2 condensates. Purified MBD2 proteins were labelled with a fluorescent probe (AF546-MBD2) and mixed with unlabelled MBD2 in a ratio of 1:99 before experiments. Then MBD2 condensate-containing solution and MBD2 homogeneous solution were mixed with heterochromatin fractions for in vitro phase separation. The condensates were imaged using a Nikon Eclipse TiE2 microscope.

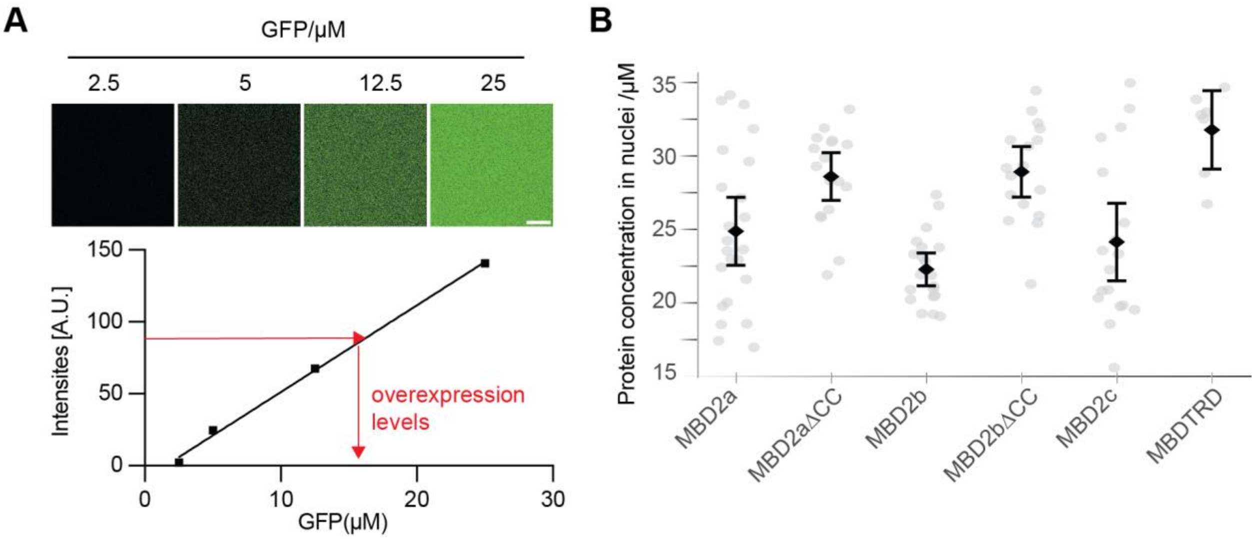

Figure S16. Quantification of ectopic GFP concentrations in live cells. (**A**) Purified GFP protein was sequentially diluted, and the relative fluorescence intensities were measured (top) under the same microscope settings, as applied in **Figure 6**. The standard curve of GFP intensities against GFP amounts was generated with a linear trend line. The ectopic GFP-MBD2 construct concentrations were calculated accordingly. (**B**) Scatter plot showing the ectopic GFP-MBD2 constructs concentrations. Data are shown by mean ± SD. Raw data can be found in Table S10.

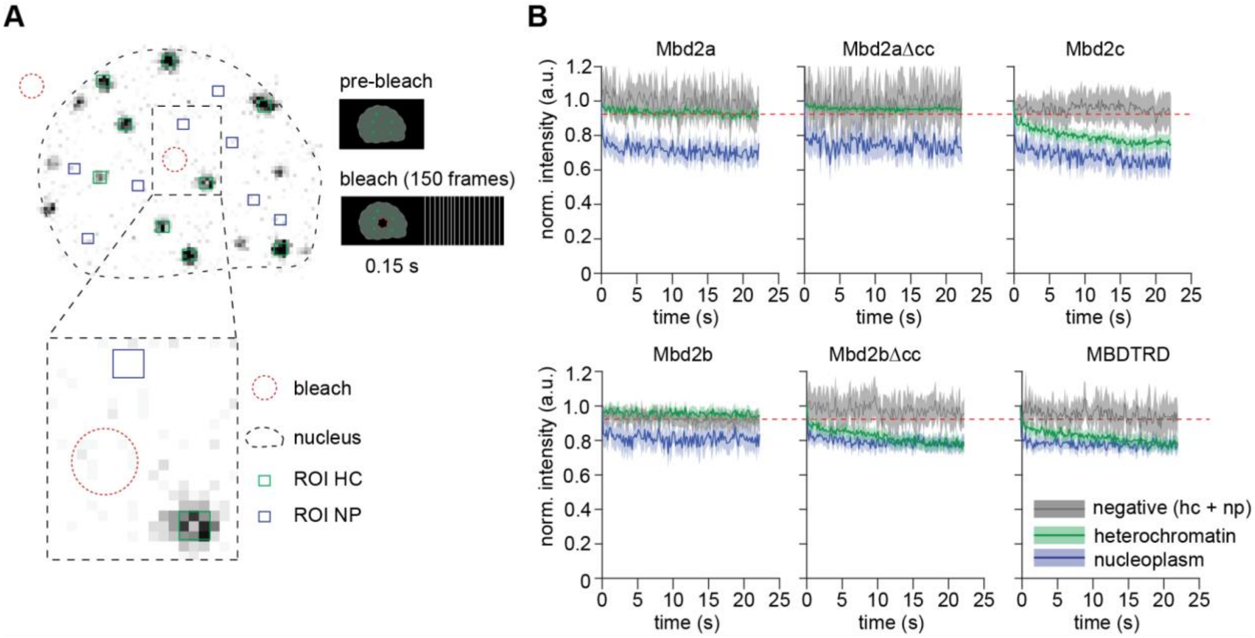

Figure S17. MBD2a/b generates interfacial barriers surrounding pericentric heterochromatin. (**A**) Scheme of the fluorescence loss in photobleaching experiment. A bleaching region was defined, either outside of the nucleus (for negative controls) or within the nucleus, and bleached during 22.5 seconds (150 frames at 0.15 s exposure time). Intensity was measured in regions with equal size (3x3 pixels) located in either nucleoplasm or heterochromatin. (**B**) Average relative intensities, normalized to the pre-bleach intensity for each region, for 12-20 cells containing different Mbd2 constructs. In each construct, the intensity of heterochromatin (green) and nucleoplasm (blue) was calculated, using cells where the bleaching region was located outside of the nucleus as a control (gray) for the photobleaching effect during the exposure time. A line for the heterochromatin plateau of Mbd2a was used for visual reference. n (MBD2a) = 7; n (MBD2aΔCC) = 12; n (MBD2c) = 8; n (MBD2b) = 16; n (MBD2bΔCC) = 20; n (MBDTRD) = 18.

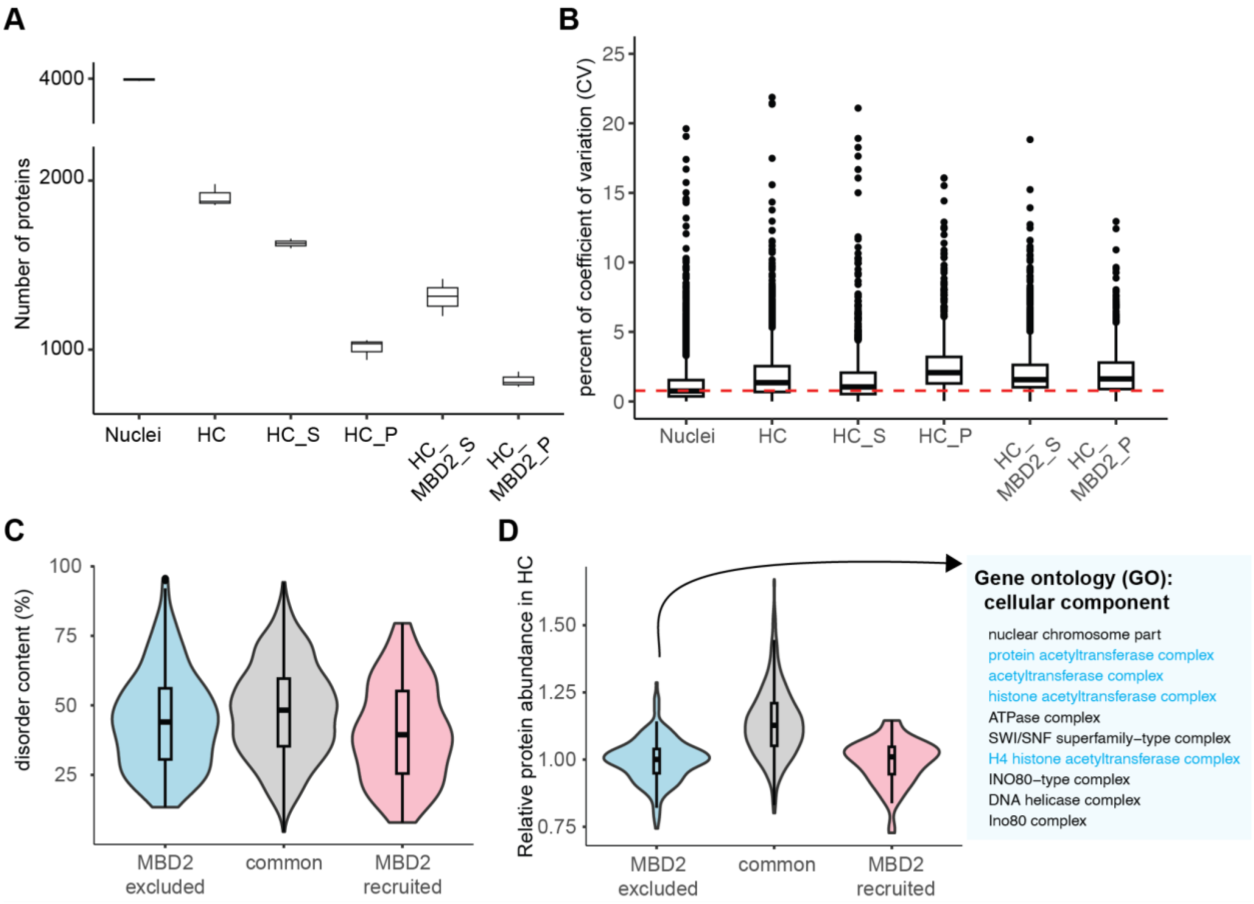

Figure S18. Mass spectrometry data analysis showing the influence of MBD2 condensates on the composition of HC phase-separated condensates. (**A**) Box plot of protein numbers in each measurement. The box plots indicate the median (central line), interquartile range (IQR) (box), and whiskers representing 1.5 × IQR. n = three replicates. Raw data can be found in Table S10. (**B**) Box plot of protein variances among the three replicates of each fraction. The red dashed line represents the mean values of percent of coefficient of variation (CV) in nuclei. The box plots indicate the median (central line), interquartile range (IQR) (box), and whiskers representing 1.5 × IQR. n = three replicates. Raw data can be found in Table S10. (**C-D**) Violin plot embedded with box plot showing the disorder scores (**C**) and protein abundance (**D**) of the proteins recognized in different fractions. Proteins in the heterochromatin condensates that were included, excluded, or not influenced by additional MBD2 condensates were analyzed. Violin plots displayed the probability density of the data at different values, mirrored around the center line. The box plots indicate the median (central line), interquartile range (IQR) (box), and whiskers representing 1.5 × IQR. Raw data can be found in Table S10. n (MBD2 excluded) = 250; n (common) = 672; n (MBD2 recruited) = 70. For excluded proteins, the Gene ontology (GO) analysis was performed (**D, right**). The protein list was subjected to the GOrilla tool (8) for gene ontology analysis in the cellular component category. The proteins recognized in the whole nucleus were applied as the background list. GO terms with a FDR q-value ≤0.05 and enrichment ≥2 were considered. The GO terms for cytoplasmic, RNA, ribosome, and nuclear membrane were removed manually. In the blue font are the acetylation related components.

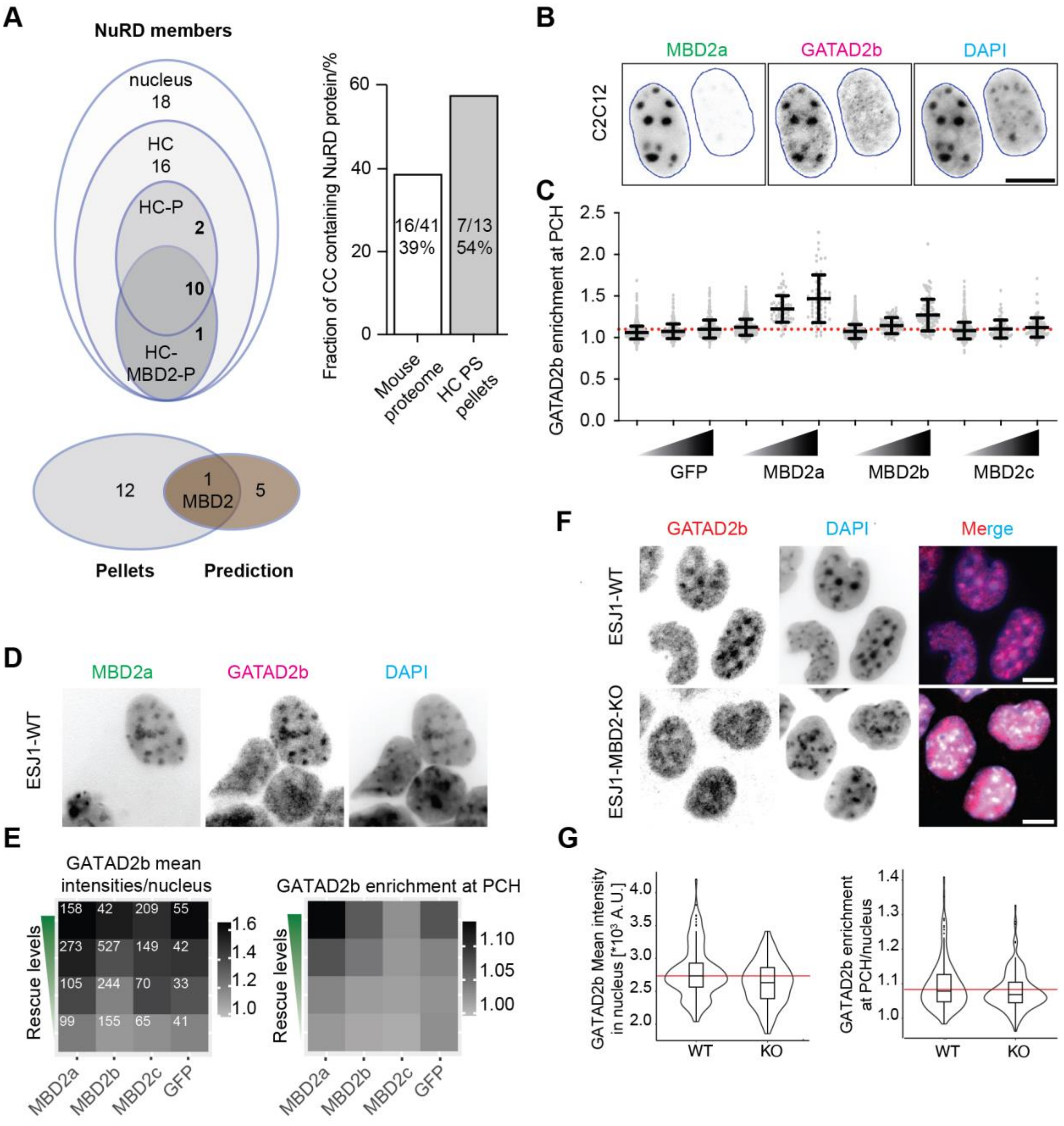

Figure S19. MBD2a/b enriches the NuRD complex at pericentric heterochromatin. (**A**) Summary of NuRD proteins in the different fractions. Top left: Venn diagram showing the number of NuRD proteins identified in different fractions derived from mouse brain nuclei. Bottom left: Venn diagram showing the overlap between NuRD proteins identified in pellets and those predicted with scaffold phase separation properties. Right: Proportion of coiled coil (CC) containing NuRD members in the whole mouse proteome and heterochromatin condensates. The CC domains were predicted using InterPro (https://www.ebi.ac.uk/interpro/). (**B**) Representative images of GATAD2b distributions in C2C12 cells expressing ectopic GFP-MBD2a. GATAD2b was visualized by immunofluorescence staining after transfection. DNA was counterstained with DAPI. Images were taken using a Nikon Eclipse TiE2 microscope equipped with a Plan Apo λ 40x air objective. (**C**) Scatter plot showing the GATAD2b fold

enrichment at pericentric heterochromatin (PCH) in C2C12 cells expressing ectopic GFP-MBD2 isoforms (MBD2a, MBD2b, and MBD2c). Cells were classified into four classes based on GFP intensities. Each class contains cells with similar GFP intensities. Data are shown by mean ± SD. n (GFP low) = 1269; n (GFP middle) = 298; n (GFP high) = 390; n (MBD2a low) = 828; n (MBD2a middle) = 61; n (MBD2a high) = 56; n (MBD2b low) = 1405; n (MBD2b middle) = 71; n (MBD2b high) = 108; n (MBD2c low) = 837; n (MBD2c middle) = 92; n (MBD2c high) = 109, (**D**) Representative images of GATAD2b distributions in wild-type ES cells expressing ectopic GFP-MBD2a. (**E**) Heat map showing the influences of MBD2 isoforms on GATAD2b abundance (left) and localization (right). Cells were classified into four classes based on GFP intensities. Each class contains cells with similar GFP intensities. Data are represented as heat maps showing the mean values. The number of cells in each condition were as shown. Data can be found in Table S10. (**F**) Representative images of GATAD2b distributions in wild type (MBD2-WT) and MBD2 knockout (MBD2-KO) ES cells. (**G**) Violin plot embedded with box plot showing the GATAD2b abundance in the whole nuclei (left) and fold enrichment at PCH (right). Violin plots display the probability density of the data at different values, mirrored around the center line. The box plots indicate the median (central line), interquartile range (IQR) (box), and whiskers representing 1.5 × IQR. red lines correspond to the relative median values in MBD2-WT cells. n (WT) = 148; n (MBD2-KO) = 186. Raw data can be found in Table S10.

SUPPLEMENTARY TABLES

Table S1: Plasmid characteristics

Name	pc number	addgene number	Fluoro phore	Gene species	Promoter	Expression	Reference
peMBD2G	pc2399	211572	EGFP	*Mus musculus*	CMV	mammals	(58, 59)
pMBD2.2-GFP	pc2068	229560	EGFP	*Mus musculus*	CMV	mammals	(59)
pmMBD2.4G	pc2841	229561	EGFP	*Mus musculus*	CMV	mammals	This study
pMBD2.1-GFP	pc2067	229559	EGFP	*Mus musculus*	CMV	mammals	(59)
pmMBD2.6G	pc2843	229562	EGFP	*Mus musculus*	CMV	mammals	(58)
pmMBD2ΔC-G	pc4794	229757	EGFP	*Mus musculus*	CMV	mammals	This study
pmMBD2ΔCC-G	pc5088	229764	EGFP	*Mus musculus*	CMV	mammals	This study
pmMBD2ΔNΔCC-G	pc5089	229765	EGFP	*Mus musculus*	CMV	mammals	This study
pmMBD2-CΔCC-G	pc5091	229766	EGFP	*Mus musculus*	CMV	mammals	This study
pmMBD2ΔCΔGR-G	pc5086	229762	EGFP	*Mus musculus*	CMV	mammals	This study
pmMBD2-NΔRG-G	pc5087	229763	EGFP	*Mus musculus*	CMV	mammals	This study
pTYB1-MBD3	pc4784	229749	-	*Mus musculus*	T7	bacteria	This study
pTYB1-MBD2	pc4786	229751	-	*Mus musculus*	T7	bacteria	This study
pTYB1-MBD2ΔN	pc4787	229752	-	*Mus musculus*	T7	bacteria	This study
pTYB1-MBD2-N	pc4789	229753	-	*Mus musculus*	T7	bacteria	This study
pTYB1-MBD2-MBDTRD	pc4791	229754	-	*Mus musculus*	T7	bacteria	This study
pTYB1-MBD2ΔC	pc4792	229755	-	*Mus musculus*	T7	bacteria	This study
pTYB1-MBD2-C	pc4793	229756	-	*Mus musculus*	T7	bacteria	This study
pTYB1-MBD2ΔCC	pc5081	229760	-	*Mus musculus*	T7	bacteria	This study
pTYB1-MBD2ΔNΔCC	pc5082	232745	-	*Mus musculus*	T7	bacteria	This study
pTYB1-MBD2-CΔCC	pc5084	229761	-	*Mus musculus*	T7	bacteria	This study
pTYB1-MBD2ΔGR	pc5078	229758	-	*Mus musculus*	T7	bacteria	This study
pTYB1-MBD2ΔCΔGR	pc5079	229759	-	*Mus musculus*	T7	bacteria	This study
pTYB1-MBD2-NΔGR	pc5083	230974	-	*Mus musculus*	T7	bacteria	This study
pUC18-MINX-M3	pc3902	-	-	Synthetic	-	PCR template	(60); (61)
pmRFP-C1-Hdac11	pc5151	248141	mRFP	*Mus musculus*	CMV	mammals	This study
pmRFP-C1-Kat7	pc5153	248143	mRFP	*Mus musculus*	CMV	mammals	This study
pmRFP-C1	pc2351	54764	mRFP	*Mus musculus*	CMV	mammals	(74)
pSpCas9(BB)-2A-Puro	pc3926	48139	-	-	CMV/U6	mammals	(75)

pc: plasmid collection. “-“: no

Table S2: Oligonucleotide characteristics

Name	Sequence [5' - 3']	Application	Reference
NdeI-MBD2-F	AAGAAGGAGATATACATATGATGCGCGCGCACCCGGGG	pTYB1-MBD2	This study
EcoRI-MBD2-R	GAAGAGCCCTCGAGGAATTCCGCCTCATCTCCATCGTC	pTYB1-MBD2	This study
NdeI-MBD3-F	AAGAAGGAGATATACATATGATGGAGCGGAAGAGGTGG	pTYB1-MBD3	This study
EcoRI-MBD3-R	GAAGAGCCCTCGAGGAATTCCACTCGCTCTGGCTCCG	pTYB1-MBD3	This study
NdeI-MBD2ΔN-F	TATGAAGAGTACATCATATGATGGACTGCCCGGCCCTC	pTYB1-MBD2ΔN	This study
EcoRI-MBD2ΔN-R	GAAGAGCCCTCGAGGAATTCCGCCTCATCTCCATCGTC	pTYB1-MBD2ΔN	This study
MBD2-C-F	AACAAGGGTAAACCAGAC	pTYB1-MBD2-C	This study
pTYB1-BB PCR-R-1	CATCATATGTATATCTCCTTCT	pTYB1-MBD2-C	This study
pTYB1-BB PCR-F-2	GAGGGCTCTTCCTGCTTTGC	pTYB1-MBD2ΔC<br>pTYB1-MBD2-MBDTRD	This study
MBD2ΔC-R-2	CTGATTGAGGGGGTCATTCCG	pTYB1-MBD2ΔC<br>pTYB1-MBD2-MBDTRD	This study
HindIII-MBD2-F	ACATATTTATAAGCTTATGCGCGCGCACCCG	pmMBD2ΔC-G	This study
SalI-MBD2ΔC-R	GCACGCATTATCGTCGACCTGATTGAGGGGGTCATTCC	pmMBD2ΔC-G	This study
pTYB1-BB PCR-F-3	GAATTCCTCGAGGGCTCT	pTYB1-MBD2-N	This study
MBD2-N-R	CCTCTTCCCGCTCTCCG	pTYB1-MBD2-N	This study
AA397-F	CGGGCTGCGGACACGGAG	CC-deletion	This study
AA362-R	GCAGAGGGGTTGAGATGTGTTAAGC	CC-deletion	This study
(GR)11-F	CCCCAGAGTGGCGGCAGCGGCCT	GR-deletion	This study
(GR)11-R	ACAGACGCCGCCGCCCCGGG	GR-deletion	This study
Fw	CGGTACCTAATACGACTCACTATA	dsDNA synthesis *	(72)
Rev 380	GTGCCAAGCTTGCATGC	dsDNA synthesis *	(72)
Rev 800	ATAGGCGTATCACGAGGC	dsDNA synthesis *	(72)
Rev 1600	TGGTCCTGCAACTTTATCCG	dsDNA synthesis *	(72)
Rev 3000	ATTCGTAATCATGGTCATAGCTG	dsDNA synthesis *	(72)
Hdac11-F	ATGCCTCACGCAACACAGCTGTACC	pmRFP-C1-Hdac11	This study
Hdac11-R	TCAAGGCACAGCACAGGAAAGCAGG	pmRFP-C1-Hdac11	This study
mRFP-Hdac11-F	TTTCCTGTGCTGTGCCTTGATTCTGCAGTCGACGGTACCG	pmRFP-C1-Hdac11	This study
mRFP-Hdac11-R	AGCTGTGTTGCGTGAGGCATACGAGATCTGAGTCCGGACTT	pmRFP-C1-Hdac11	This study

Kat7-F	ATGGCGATAGGTGTTGTAAAGAGAA	pmRFP-C1-Kat7	This study
Kat7-R	ATAGGTCACTTTAAGTGCCCTTGGG	pmRFP-C1-Kat7	This study
pmRFP-C1-Kat7-F	GGGCACTTAAAGTGACCTATTTCTGCAGTCGACGGTACCG	pmRFP-C1-Kat7	This study
pmRFP-C1-Kat7-R	CTTTACAACACCTATCGCCATAGATCTGAGTCCGGACTTG	pmRFP-C1-Kat7	This study
MBD2-gRNA-F	caccgCATCCTCTTCCCGCTCTCCG	pSpCas9-2A-Puro-MBD2	This study
MBD2-gRNA-R	aaacCGGAGAGCGGGAAGAGGATG	pSpCas9-2A-Puro-MBD2	This study

*dsDNA used in in vitro phase separation assay (Figure S8)

Table S3: Bacterial and mammalian cell line characteristics

Name	Species	Purpose	Genotype	Reference
Top 10	*Escherichia coli*	plasmid DNA production	F^– mcrA Δ(mrr-hsdRMS-mcrBC) φ80lacZΔM15 ΔlacX74 recA1 araD139	Invitrogen
BL21(DE3)	*Escherichia coli*	IPTG induced gene expression	F – ompT hsdSB (rB- mB-) gal dcm (DE3)	(76)
BL21(DE3) pLysS	*Escherichia coli*	IPTG induced gene expression	F- ompT hsdSB (rB- mB-) gal dcm (DE3) pLysS (CamR)	(76)
BL21(DE3) Star	*Escherichia coli*	IPTG induced gene expression	F-ompT hsdSB (rB- mB-) gal dcm rne131 (DE3)	New England Biolabs GmbH (NEB)
C2C12	*Mus musculus*	Mammalian gene expression (myoblast)	wildtype	(77)
HEK293-EBNA	*Homo sapiens*	Co-immunoprecipitation	wildtype	Invitrogen
ES J1	*Mus musculus*	Mammalian gene expression; MBD2 knockout	wildtype	(78)
ES J1 MBD2 KO	*Mus musculus*	MBD2 function	MBD2 triple knockout (KO)	this study

Table S4: Imaging systems characteristics

Microscope /Company	Lasers/lamps	Filters (ex. & em. [nm])*	Objectives/ lenses	Detection system	Incubation system	Application	Software
VWR	UV Fluorescence	-	-	Camera: H6Z0812 8-48 mm	-	EtBr staining	-

Amersham AI600 imager	white transillumination: 470 – 635 nm;<br>UV transillumination: 312 nm	-	-	16-bit Pelltier cooled Fujifilm Super CCD	-	Western blot, coomassie blue stained gels	Amersham AI600 imager analysis software
Nikon CREST	SPECTRA X LED 470/24 nm (196 mW)<br>640/30 nm (231 mW)	em.: Quadbandpass (432/25 nm; 515/25 nm; 595/25 nm; 730/70 nm)	40x air Plan Apo λ DIC (0.95 NA, 230 µm WD)	Nikon Qi2 751600 16.25 MPx	-	phase separation	NIS Elements Advanced Research (AR)
Confocal microscope Leica TCS SP5-II	405 nm diode laser 50 mW;<br>488 nm Argon ion laser:<br>458 nm ~5 mW<br>476 nm ~5 mW<br>488 nm ~20 mW<br>496 nm ~5 mW<br>514 nm ~20 mW	DAPI:<br>ex. 420/30<br>em. 465/20<br>FITC:<br>ex. 495/15<br>em. 530/30<br>Rhod:<br>ex. 570/20<br>em. 640/40	HCX PL APO 100x / 1.44 oil Corr CS	HyD Hybrid Detectors	ACU live cell chamber (Olympus)	fixed and live cell imaging	Leica Application Suite Advanced Fluorescence (LAS AF)

*ex.: excitation; em.: emission; EtBr: ethidium bromide; ** AOTF: Acousto-optic tunable filters; UV: ultraviolet; CCD: charge-coupled device; DIC: differential interference contrast; NA: numerical aperture; WD: Working distance; DAPI: 4′,6-diamidino-2-phenylindole; FITC: fluorescein isothiocyanate.

Table S5: Primary and secondary antibody characteristics

Reactivity	Host	Dilution	Application	Catalog/clone	Company/reference
anti-beta III tubulin	rabbit	1:1,000	WB	ab52623	Abcam
anti-GATAD2b	rabbit	1:250	IF	AB-2641884	Invitrogen
anti-GFP	rat	1:1000	WB	Clone 3H9	Chromotek,Planegg-Martinsried, Germany
anti-H1	rabbit	1:1000	WB	ab134914	Abcam
anti-H1	mouse	4 µg/ml	WB	sc-8030	Santa Cruz
anti-H3K27ac	rabbit	1:1,000	IF	D5E4	Cell Signalling Technology
anti-H3K9ac	rabbit	1:200	IF	39917	Active Motif
anti-H4K8ac	rabbit	1:200	IF	ab15823	Abcam
anti-HP1a	mouse	1:500	WB	MAB3584	Active Motif
anti-lamin A/C	rabbit	1:2000	WB	-	Gift from Brian Burke

anti-lamin B	mouse	1:10	WB	61047C	Progen Biotechnik GmbH
anti-MBD2	rabbit	1:100/1:1000	IF/WB	ab188474	Abcam
anti-MBD2	rabbit	1:100/1:1000	IF/WB	RA-18	Merck
anti-MeCP2	rat	Undiluted TCSN	WB	4H7	(44)
anti-RFP	rat	1:500	WB	Clone 5F8	(79)
GFP binder	nanobody	1 mg/mL	coIP	-	(42)
anti-mouse IgG Cy5	donkey	1:500	WB/IF	JIM-715-175-150	Jackson ImmunoResearch Europe Ltd.
anti-rabbit IgG Cy5	donkey	1:1000 (WB) 1:400 (IF)	WB/IF	JIM-715-175-152	Jackson ImmunoResearch Europe Ltd.
anti-rat IgG Cy3	donkey	1:1000	WB	JIM-712-165-153	Jackson ImmunoResearch Europe Ltd.
anti-rat IgG HRP	sheep	1:1000	WB	A9037	Sigma-Aldrich, St Louis, MO, USA

WB: western blot; IF: immunofluorescence staining; TCSN: tissue culture supernatant; coIP: co-immunoprecipitation; HRP: horseradish peroxidase.

Table S6: Mass spectrometry-related instruments and software

Application	Instrument/Software	Description	Vendor
Native mass spectrometry	Sutter P-97 needle puller	Produces glass needles for nESI	Sutter Instrument
	Synapt XS ion mobility-mass spectrometer	nESI-Q-ToF mass spectrometer	Waters Corporation
	MassLynx V4.2	Software for Synapt management and raw data processing	Waters Corporation
LC-MS/MS	Easy nano LC 1200	Liquid chromatography system	Thermo Fisher Scientific
	Orbitrap Exploris 480	Orbitrap Mass Spectrometer	Thermo Fisher Scientific
	DIA-NN 1.7.17 beta 12	Software for DIA proteomics raw data processing	(80)
	Perseus (version 1.6.0.9)	Software for proteomics data analysis	(29)

nESI: nanoelectrospray ionization ; Q-ToF: quadrupole time-of-flight; LC-MS/MS: liquid chromatography with tandem mass spectrometry; DIA:data-independent acquisition.

Table S7: Public servers and datasets

Name	Description	links	Reference
DisProt	known intrinsically disordered proteins	https://www.disprot.org/	(30)
PhaSepDB	reported phase separations	http://db.phasep.pro/	(31)
LLPSDB	reported phase separations	http://www.bio-comp.org.cn/LLPSDB/home.html	(32)
PhaSePro	reported phase separations	https://phasepro.elte.hu/	(33)
DrLLPS	phase separation prediction tools	https://llps.biocuckoo.cn/	(24)
PSAP	phase separation prediction tools	https://github.com/vanheeringen-lab/psap	(23)
PhaSePred	phase separation prediction tools	http://predict.phasep.pro/	(22)

PSPHunter	tools to predict phase-separating proteins, key residues, or mutation effect	http://psphunter.stemcellding.org/index.php	(36)
UniProt	free resource of protein sequence and functional information	https://www.uniprot.org/	(34)
AlphaFold server	highly accurate biomolecular structure prediction tools	https://alphafoldserver.com/	(35)
Half-FRAP	a quantitative approach to assess if molecules in a structure of interest undergo liquid-liquid phase separation or polymer-polymer phase separation	https://colab.research.google.com/github/dymochro/MOCHA/blob/main/MOCHA_ColabNotebook.ipynb	(46)
InterPro	InterPro provides functional analysis of proteins by classifying them into families and predicting domains and important sites.	https://www.ebi.ac.uk/interpro/	(81)
PaxDb	PaxDb is a comprehensive absolute protein abundance database, which contains whole genome protein abundance information across organisms and tissues.	https://beta.pax-db.org/	(82)
